# Neighbourhood level real-time forecasting of dengue cases in tropical urban Singapore

**DOI:** 10.1186/s12916-018-1108-5

**Published:** 2018-08-06

**Authors:** Yirong Chen, Janet Hui Yi Ong, Jayanthi Rajarethinam, Grace Yap, Lee Ching Ng, Alex R. Cook

**Affiliations:** 1 Saw Swee Hock School of Public Health, National University of Singapore and National University Health System, 12 Science Drive 2, Singapore, 117549 Singapore; 2 Environmental Health Institute, 11 Biopolis Way, Singapore, 138667 Singapore

**Keywords:** Spatio-temporal prediction, Dengue forecast, LASSO, Control and prevention

## Abstract

**Background:**

Dengue, a vector-borne infectious disease caused by the dengue virus, has spread through tropical and subtropical regions of the world. All four serotypes of dengue viruses are endemic in the equatorial city state of Singapore, and frequent localised outbreaks occur, sometimes leading to national epidemics. Vector control remains the primary and most effective measure for dengue control and prevention. The objective of this study is to develop a novel framework for producing a spatio-temporal dengue forecast at a neighbourhood level spatial resolution that can be routinely used by Singapore’s government agencies for planning of vector control for best efficiency.

**Methods:**

The forecasting algorithm uses a mixture of purely spatial, purely temporal and spatio-temporal data to derive dynamic risk maps for dengue transmission. LASSO-based regression was used for the prediction models and separate sub-models were constructed for each forecast window. Data were divided into training and testing sets for out-of-sample validation. Neighbourhoods were categorised as high or low risk based on the forecast number of cases within the cell. The predictive accuracy of the categorisation was measured.

**Results:**

Close concordance between the projections and the eventual incidence of dengue were observed. The average Matthew’s correlation coefficient for a classification of the upper risk decile (operational capacity) is similar to the predictive performance at the optimal 30% cut-off. The quality of the spatial predictive algorithm as a classifier shows areas under the curve at all forecast windows being above 0.75 and above 0.80 within the next month.

**Conclusions:**

Spatially resolved forecasts of geographically structured diseases like dengue can be obtained at a neighbourhood level in highly urban environments at a precision that is suitable for guiding control efforts. The same method can be adapted to other urban and even rural areas, with appropriate adjustment to the grid size and shape.

**Electronic supplementary material:**

The online version of this article (10.1186/s12916-018-1108-5) contains supplementary material, which is available to authorized users.

## Background

Dengue, a vector-borne infectious disease caused by the dengue virus (DENV, four serotypes DENV1–4), has spread through tropical and subtropical regions of the world in recent decades [1]. It is transmitted by the *Aedes* mosquitoes, and in urban areas, primarily by the anthropophilic *Aedes aegypti*. The total number of dengue infections globally has been estimated to be 390 million per year [2], of which 96 million manifest clinically, the majority of which (70%) are found in Asia. It has been estimated that 3.97 billion people from 128 countries are at risk of dengue infection [3], and as urbanisation continues across much of Asia [4], the incidence is liable to grow [5]. Dengue fever usually leads to self-limiting symptoms including fever, headaches, pain behind the eyes, nausea, vomiting, swollen glands, rash, and joint, bone, or muscle pains [6]. However, when dengue fever develops into severe dengue, then plasma leakage, severe bleeding, severe organ impairment, and even death may occur [7], making dengue control an important public health problem.

In the equatorial city state of Singapore, since the 1990s there has been a dramatic increase in the number of notified dengue cases, and all four serotypes are endemic [8]. Singapore’s favourable climatic condition (average monthly temperature varying from 26 to 28 °C), its highly urbanised environment and its being a hub for international travel and transition [9] make it ideal for the breeding of *Aedes* mosquitoes and transmission of dengue. Since 2013, a dengue incidence of more than 150 per 100,000 population has been reported [10] and this has been related to a sizable disease burden to Singapore [11]. Although a new dengue vaccine, Dengvaxia® (CYD-TDV), first licensed in Mexico in 2015 [12], has been approved by the Health Science Authority in Singapore for persons aged 12 to 45, and has been available commercially since 2017, the vaccine is primarily effective against DENV3 and DENV4 but less so against DENV1 and DENV2 [13], which are the predominant serotypes in Singapore [14]. The vaccine is more effective for individuals with a prior exposure to dengue virus [12], but increases the risk of severity in subsequent infection for immune naïve individuals [15]. It is, thus, not recommended for Singapore where endemicity and seroprevalence are low [16, 17].

At present, vector control remains the primary and most effective measure for dengue control and prevention [18]. The National Environment Agency (NEA) of Singapore deploys officers to inspect premises, eliminate potential breeding grounds and outreach to remind residents to remove sources of stagnant water. Such resource-intensive vector control measures could be optimised by targeting areas with a greater risk of transmission.

As well as providing an indication of where dengue transmission is ongoing, incident case data also foreshadow where future outbreaks are most likely, and hence provide a guide to which areas could be prioritised for preventive efforts. To do so requires quantifying the likely number of cases in different areal units, which can be addressed through short-term forecasting.

In the literature, various models have been proposed for the prediction of dengue cases. Machine learning methods (including the support vector regression algorithm, gradient boosted regression tree algorithm, and regression or auto-regression models) have been used at national [19], sub-national [20] and urban levels [21], using incidence and climatic variables, including temperature, relative humidity, rainfall and solar radiation. Examples from Singapore [22–24] have provided forecasts at a national level, with the Environment Health Institute in Singapore currently relying on least absolute shrinkage and selection operator (LASSO) based models, incorporating recent case data, meteorological data, vector surveillance data and population-based national statistics, to derive up to 3-month national forecasts to guide vector control [24]. In the past 5 years, extensive work has been done in many dengue-affected areas in the world on dengue forecasting, including Thailand, Indonesia, Ecuador and Pakistan [25–29], to create early warnings of potential dengue outbreaks. In addition to the conventionally used meteorological or disease epidemiological information as predictors [23, 30, 31], recent forecast models have begun to incorporate human mobility information [32, 33], land use [34], frequency of social media mentions and appearances on online search engines [35, 36], and spatial dynamics [37–39] to provide additional information for accurate predictions.

Even within a small city state such as Singapore, spatial variations in risk may be profound, reflecting differences in urban density, the presence of natural areas (such as rainforest and reservoirs) and differential age profiles of different housing estates, and as such, a finer resolution forecast, if one were available, would potentially allow better targeting of the response. The objective of this study is, therefore, to develop a new approach for spatio-temporal dengue forecasting at a finer spatial resolution that can be routinely used by Singapore’s government agencies for planning of vector control for best efficiency, and which may potentially be adapted to other settings.

## Methods

### Modelling objectives

Our objective is to develop a suite of models, each of which will make a forecast for one specified time window, based on the data available at the time the forecast is made. Each model will predict for each neighbourhood the number of cases within a 1-week interval, which will then be used to rank neighbourhoods according to projected risk. This ranking can then be used to identify those areas to be prioritised for interventions, subject to resource availability. Accuracy will be assessed by correlating observed and actual numbers of cases and calculating the receiver operating characteristics when neighbourhoods are classified as high or low risk.

### Source of data

The forecasting algorithm uses a mixture of purely spatial, purely temporal and spatio-temporal data to derive dynamic risk maps for dengue transmission.

#### Spatio-temporal

The Ministry of Health, Singapore, continuously monitors the incidence of dengue through mandatory notification of virologically confirmed or laboratory-confirmed cases. The residential address and date of onset of each case in Singapore are recorded. We aggregated individual-level data into weekly number of cases in 315 spatial units of size 1 km × 1 km (henceforth, *neighbourhoods*), from 2010 to 2016, spanning the major residential areas of the country.

The movement patterns of mobile subscribers were derived by analysing their cell phones’ network activities among subscribers of Starhub Ltd, one of the three major mobile telephone companies (telcos) in Singapore. These data were aggregated and used to determine the connectivity between different neighbourhoods, which was subsequently used to derive a variable we called the *connectivity-weighted transmission potential*, which captures the future risk to a neighbourhood from other neighbourhoods with current dengue cases, based on the amount of movement from one neighbourhood to the other. A detailed description of these data is provided in Additional file 1.

Building age was obtained from the Housing Development Board and the Urban Redevelopment Authority and averaged over all buildings within a neighbourhood. Previous studies have shown that the quality of buildings can impact the presence of potential breeding habitats [40], thus increasing the risk of dengue transmission. Because building practices have evolved over time and newer buildings are designed to reduce vector breeding sites, building age is a plausible risk factor for transmission, and as preliminary analyses showed a high association with both *Aedes* mosquito and dengue incidence, this was used as a predictor in the model.

Meteorological data are incorporated to account for the important role that climate has in the mosquito life cycle. Despite Singapore’s small size, there are some systematic differences in climate across the country [41], and to accommodate that, meteorological data were estimated for each neighbourhood using weekly mean, maximum and minimum temperature, and average relative humidity from the nearest (of 21) weather stations across the island managed by the Meteorological Services Singapore.

#### Temporal

Other than weekly incidence in the cells, individual-level dengue incidence data were aggregated into weekly national cases as a proxy for the general epidemic level.

#### Spatial

The vegetation index refers to the Normalised Difference Vegetation Index (NDVI), which is an index of plant viridescence or photosynthetic activity. NDVI is based on the observation that different surfaces reflect different types of light differently. NDVI data were obtained from the Centre for Remote Imaging, Sensing and Processing in the National University of Singapore from a processed satellite image. Travel history data derived from trips made using EZLink cards (a card to pay for public transport fares in Singapore) were used to measure how connected each neighbourhood is to other parts of the country by public transport. These were processed and aggregated by the provider, prior to analysis, which derived a connectivity ranking based on the number of trips in and out of each cell (as described in the Additional file 1). The cells were ranked by percentile to form the connectivity ranking. In contrast to the telco data, this data source captures short transits through neighbourhoods.

The Institutional Review Board of the National University of Singapore provided the ethical approval for this study.

### Statistical analysis

LASSO regression was used for the prediction models [42]. In contrast to standard linear regression in which parameters are estimated by minimising the sum of squares of residuals, LASSO regression imposes an extra constraint that the sum of the absolute value of the regression coefficients be less than a fixed value, which is selected for optimal out-of-sample predictive performance. This algorithm shrinks coefficients towards zero, with some becoming exactly zero, and hence, the covariates associated with these coefficients are not associated with the outcome variable in the model. Compared to a simple regression, which estimates coefficients for a pre-specified set of predictors, a LASSO regression allows all covariates, at multiple lags, to be included as potential predictors, despite the usual concerns about the size of the variable space or the presence of collinearities. The optimal balance between model accuracy and complexity is obtained by varying the constraint and optimising out-of-sample predictive accuracy over the data not used in the model building process, which is inherently well suited to the problem of forecasting, as described in earlier non-spatial work [24, 43].

Separate LASSO sub-models were constructed for each forecast window, which were defined as the number of weeks ahead the sub-model is predicting. All 315 (approximate) squares of size 1 km × 1 km covering residential areas of Singapore were included in each sub-model. For each sub-model, information for all 315 grid neighbourhoods at all time points in the training set were included. Each candidate predictor appeared several times in each sub-model, at different historical lags. To allow for contagion and typical epidemic duration, we used past incidence of up to 8 weeks. To accommodate non-linearities, we also used past incidence squared, cubic, and square root, up to 8 weeks in the past. Polynomials are commonly used to approximate any non-linearity in the relationship between the covariate and outcome, and thus, we allow (but do not force) polynomial terms to account for potential non-linearities between future number of cases and autoregressive terms. In addition, the total number of cases in nearby areas were included at up to 8 weeks lag. Two tiers of nearby areas were used: within 1 km radius and within a ring from 1 km to 2 km from the centroid of the neighbourhood of interest. These are depicted in Additional file 2: Figure S1. Climatic variables (average, minimum and maximum temperature, and humidity) of up to 5 weeks’ lag were included. Cells were included in the analysis if the centroid falls within a residential area of Singapore; some cells near the boundary are truncated to the part on the main island, Pulau Ujong.

For each forecast window (from *k* = 1 to 12 weeks), a separate LASSO sub-model was developed, which used data available at the time of the forecast only. Each LASSO sub-model is as follows:

$$ {y}_{t+k,i}={\alpha}_k+{\sum}_{l=0}^7{\beta}_{k_1,l}{y}_{t-l,i}+{\sum}_{l=0}^7{\beta}_{k_2,l}{y}_{t-l,i}^2+{\sum}_{l=0}^7{\beta}_{k_3,l}{y}_{t-l,i}^3+{\sum}_{l=0}^7{\beta}_{k_4,l}\sqrt{y_{t-l,i}}+{\sum}_{r=1}^2{\sum}_{l=0}^7{\varphi}_{k_r,l}{\mathrm{n}}_{t-l,i,r}+{\sum}_{c=1}^4{\sum}_{l=0}^4{\gamma}_{k_c,l}{W}_{t-l,i,c}+{\lambda}_k{T}_{t,\kern0.5em i}+{\theta}_k{A}_{t,\kern0.5em i}+{\delta}_k{N}_t+{\upomega}_k{V}_i++{\uprho}_k{U}_i+{\varepsilon}_k, $$

where *y*_*t*, *i*_ is the number of cases (natural log-transformed, with 1 added to avoid logging 0) in neighbourhood *i* in week *t*. The terms ${y}_{t,i}^2$, ${y}_{t,i}^3$ and $\sqrt{y_{t,i}}$ are the square, cubic and square root of the number of cases. Similarly, *n*_*t*, *i*, 1_ and *n*_*t*, *i*, 2_ are the total number of cases (similarly, natural log-transformed, with 1 added to avoid logging 0) from all neighbourhoods whose centroids are within 1 km radius and within a ring from 1 km to 2 km from the centroid of neighbourhood *i*, in week *t*, respectively. *W*_*t*, *i*, *c*_ represents the climatic variable (average, minimum and maximum temperature, and average relative humidity) at time *t* in neighbourhood *i*. *T*_*t*, *i*_ measures the number of cases moving into neighbourhood *i* in week *t*, derived from a one-time telco dataset on the movement of users. *A*_*t*,*i*_ measures average building age in neighbourhood *i* in week *t*. *N*_*t*_ is the national total number of cases (natural log-transformed, with 1 added) in week *t*. *V*_*i*_ and *U*_*i*_ measure the vegetation and connectivity index of neighbourhood *i*. Detailed information on the type of each set of variables are documented in Additional file 3: Table S1. Covariates in the LASSO regression were *z*-scored prior to estimation and the coefficients were rescaled afterwards.

Parameter estimation was subject to the LASSO constraint: ${\sum}_{j=1}^4{\sum}_{l=0}^7\left|{\beta}_{k_j,l}\right|+{\sum}_{r=1}^2{\sum}_{l=0}^7\left|{\varphi}_{k_r,l}\right|+{\sum}_{c=1}^4{\sum}_{l=0}^4\left|{\gamma}_{k_c,l}\right|+\left|{\lambda}_k\right|+\left|{\uptheta}_k\right|+\left|{\delta}_k\right|+\left|{\omega}_k\right|+\left|{\rho}_k\right|\le p$. Ten-fold cross validation was performed and the constraint term that optimised the out-of-sample performance was chosen as the optimal *p* for the forecast model.

As the models were built separately for each forecast window, the variables included in the final forecast model and their lags and parameter magnitude and sign may differ substantially.

LASSO models were built using all the data from the training dataset, which comprised information from 2010 to 2015. Out-of-sample validation was performed on the testing dataset consisting of data from 2016.

### Effect size

The effect size of each predictor at different time lags and for different forecast windows and the corresponding 95% confidence intervals were derived by taking 1000 bootstrap samples and fitting LASSO models to them. We used a standard bootstrap algorithm to derive 95% confidence intervals from the lower and upper 2.5 percentiles of the bootstrap sampling distribution of the LASSO estimates. The ranges and distributions of all predictor values were derived based on the training set and the effect size obtained by multiplying the LASSO coefficient and values within the range.

### Forecast

As well as the forecast number of cases per neighbourhood, we categorised neighbourhoods as being low or high risk, as follows. The predicted number of cases for each neighbourhood was derived using information only up to when the predictions were made. Model parameters were derived from model fitting using only the training dataset. At each forecast time point, neighbourhoods were ordered by the predicted number of cases and categorised as high risk if they were in the upper decile (i.e. top 32 neighbourhoods out of 315 residential areas) for that time point. The choice of dichotomising at 10% was taken considering the operating capacity of the NEA for vector control. Predicted cases during the validation period (2016) constitute a genuine out-of-sample forecast. During the training period (2010–2015), the full time span was used to estimate parameters, but only covariates available at the time of the forecast were used to make the forecast. As such, predictive accuracy may be slightly overstated for the training period.

### Accuracy

In the model building, predictive accuracy was measured using the root-mean-square error. Subsequently, we assessed the predictive accuracy by evaluating the accuracy of their categorisation of high-risk areas for the validation dataset. For each forecast window, a receiver operating characteristic (ROC) curve —frequently used to evaluate classifiers’ performance—was derived [44]. Predictions and classifications at all 40 prediction time points were aggregated to derive one ROC curve for each forecast window. Given the actual classification of high- and low-risk neighbourhoods based on observed actual incidences (i.e. the 10% of neighbourhoods with the greatest number of cases were classified as high risk) and our forecast models, the ROC curve demonstrates relative trade-offs between true positives and false positives. The area under the ROC curve (AUC), a commonly used measurement to summarise the two-dimensional ROC performance as a single value between 0 and 1 [45], was derived for each forecast window. ROC, AUC and their respective confidence intervals were obtained using 50 bootstrap samples. A baseline level AUC was also derived using the temporal average of the number of cases from all previous years as the prediction for all 40 prediction time points, and we computed the AUC by comparing this “prediction” with the actual observed distribution of cases.

To assess the robustness of the findings to the choice of the 10% cut-off we currently adopted for the categorisation, an average Matthew’s correlation coefficient was calculated for each forecast window at 14 different cut-off points (1%, 3%, 5%, 10%, 15%, 20%, 25%, 30%, 40%, 50%, 60%, 70%, 80% and 90%). This measures the correlation coefficient between the observed and predicted binary classification, and thus the quality of binary classifications [46], and takes a value from − 1 to 1 with 1 indicating perfect agreement, 0 indicating no better than random and − 1 indicating total disagreement. Matthew’s correlation coefficient was computed for each forecast window at all prediction time points and averaged over time to derive an average coefficient for each forecast window.

All statistical analysis were performed using statistical software R [47].

## Results

**Fig. 1:**
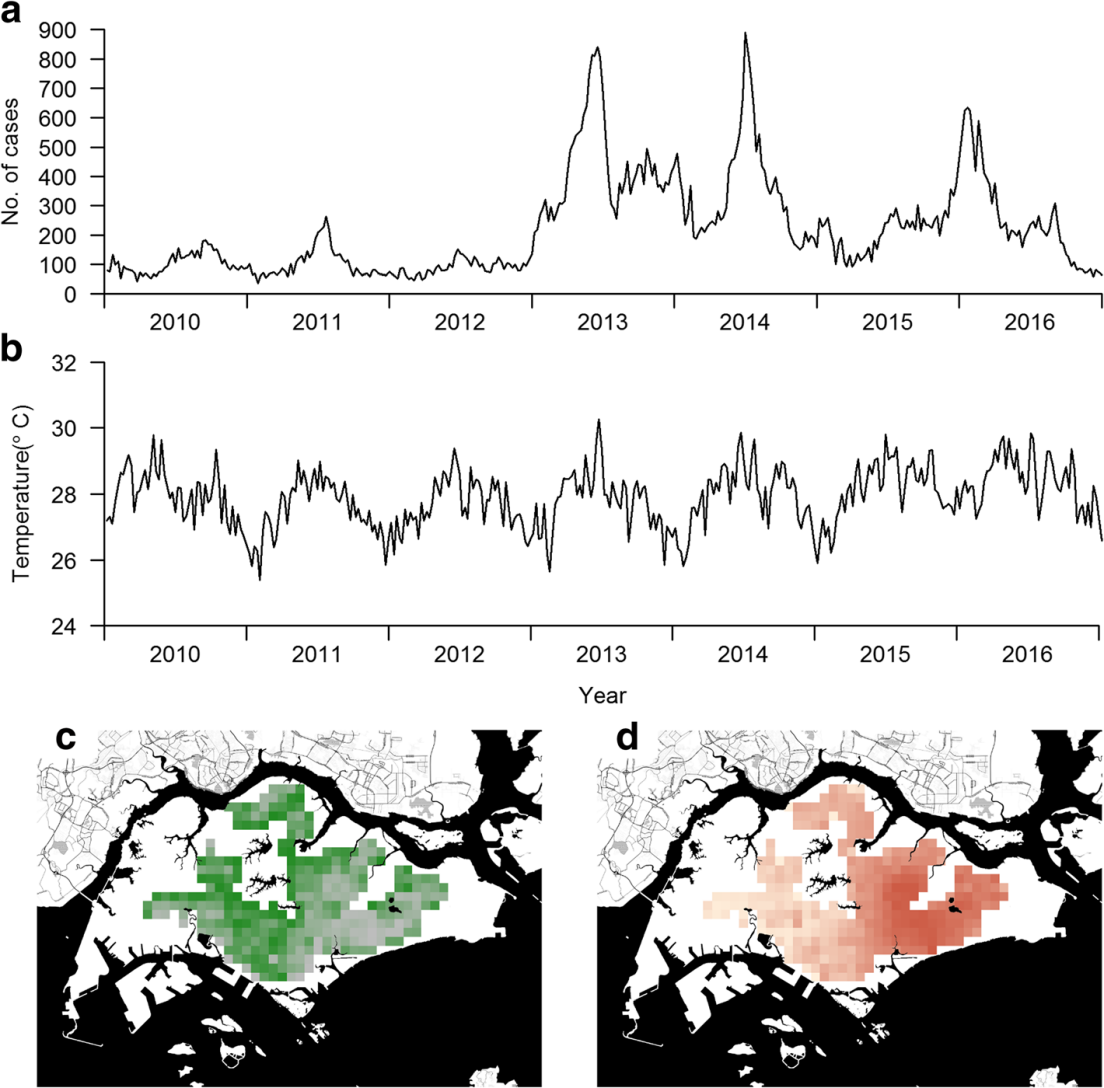
Time series and spatial density of selected predictors in the LASSO model. **a** Time series of weekly national number of cases from 2010 to 2016. **b** Time series of average temperature for one arbitrarily selected residential neighbourhood from 2010 to 2016. **c, d** Density of vegetation and movement for one arbitrarily selected time point for all 315 residential neighbourhoods

Selected independent variables in the prediction model are presented in Fig. 1. A mix of spatial and temporal variables are shown (other independent variables are presented in Additional file 4: Figure S2, Additional file 5: Figure S3, Additional file 6: Figure S4, Additional file 7: Figure S5, Additional file 8: Figure S6, Additional file 9: Figure S7, Additional file 10: Figure S8, Additional file 11: Figure S9 and Additional file 12: Figure S10). There are no strong annual cycles in either case counts (Fig. 1a) or climatic variables (Fig. 1b, Additional file 7: Figure S5, Additional file 8: Figure S6, Additional file 9: Figure S7 and Additional file 10: Figure S8). The geographic distribution of greenery is shown in Fig. 1c, while case movement data for a random week derived from the telco information on movement of the general population is shown similarly on a heat map in Fig. 1d.

**Fig. 2:**
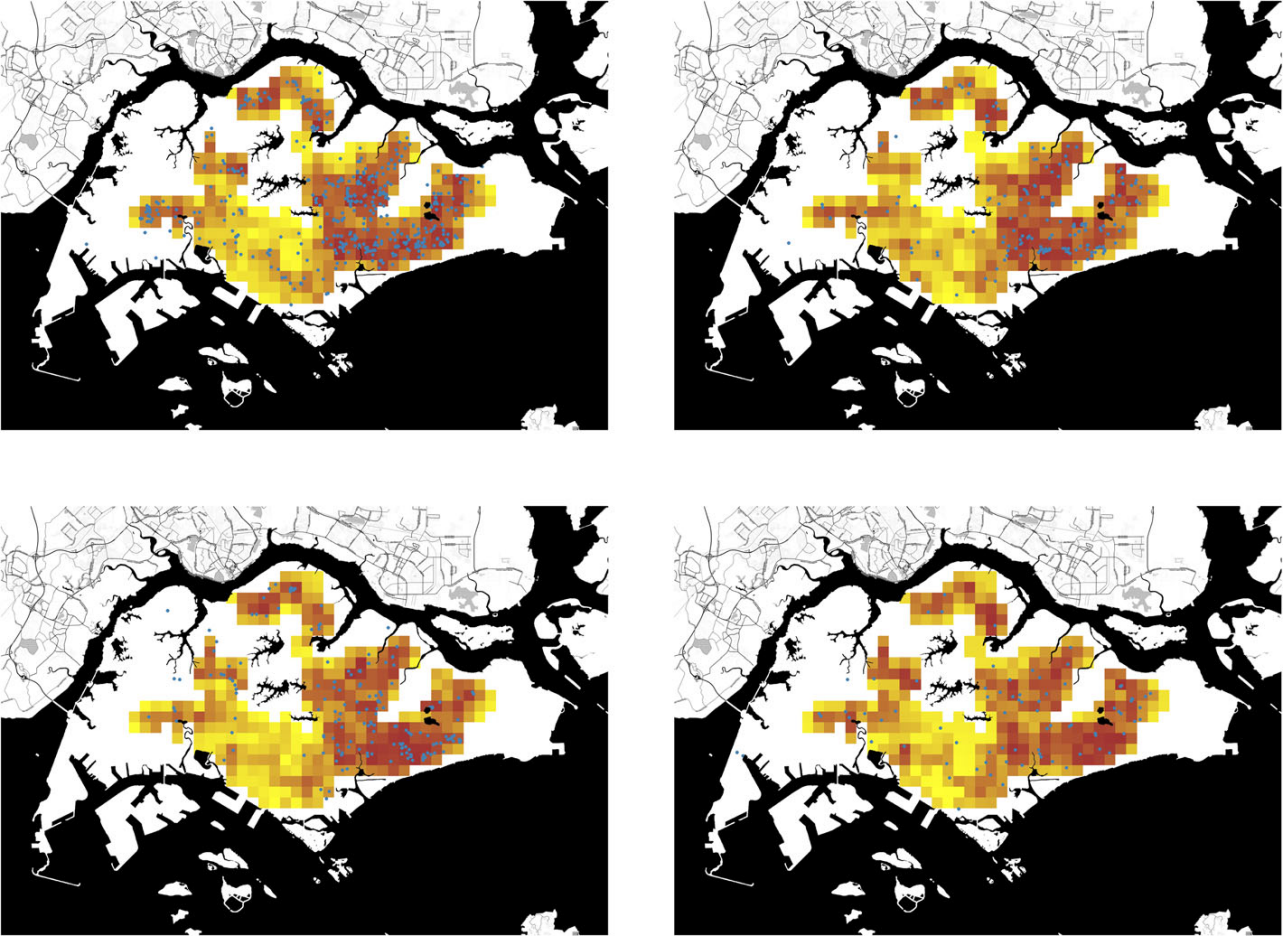
Actual distribution of cases (dark blue dots) and 4-week ahead forecasts of density at four time points (epidemiological weeks 1, 14, 27 and 40 for 2016). Yellow indicates neighbourhoods with relatively fewer predicted cases and dark red indicates those with relatively more predicted cases

**Fig. 3:**
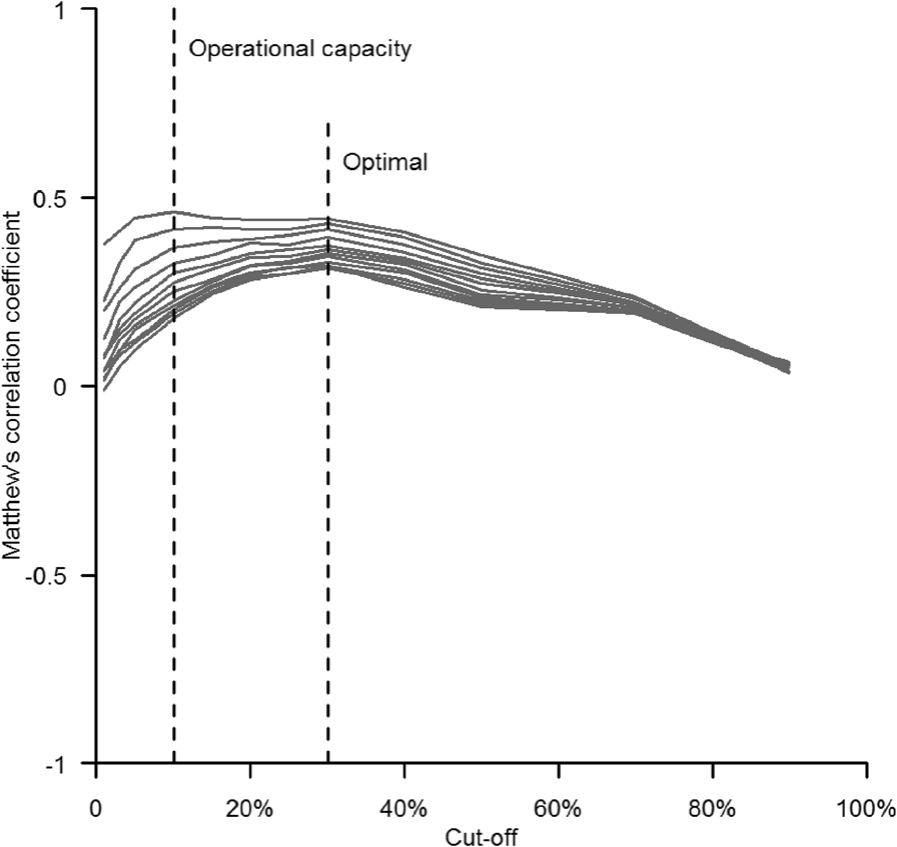
Average Mathew’s correlation coefficient for all 12 forecast windows at 14 different cut-offs (1%, 3%, 5%, 10%, 15%, 20%, 25%, 30%, 40%, 50%, 60%, 70%, 80% and 90%). Cut-off are set at different levels so that different percentages of the neighbourhoods are classified as higher risk areas


Figure 2 shows the forecast and actual distribution of dengue incidence at four distinct time points (epidemiological weeks 1, 14, 27 and 40 for 2016) for 4-week ahead forecasts (predictions at other time points are presented in Additional file 13: Video S1, Additional file 14: Video S2, Additional file 15: Video S3, Additional file 16: Video S4, Additional file 17: Video S5, Additional file 18: Video S6, Additional file 19: Video S7, Additional file 20: Video S8, Additional file 21: Video S9, Additional file 22: Video S10, Additional file 23: Video S11 and Additional file 24: Video S12 for forecast windows 1 to 12). These demonstrate the close concordance between the projections and the eventual incidence. The average Matthew’s correlation coefficient for all 12 forecast windows at 14 different risk classification cut-offs are shown in Fig. 3 (and tabulated in Additional file 25: Table S2). For most of the forecast windows, a classification of the upper risk decile—the operational capacity—as high risk had similar predictive performance as the optimal (30%).

**Additional file 13**: **Video S1**. Video of 1-week ahead forecast and actual distribution of dengue incidence in 2016. (MP4 1177 kb)



**Additional file 14: Video S2**. Video of 2-week ahead forecast and actual distribution of dengue incidence in 2016. (MP4 1159 kb)



**Additional file 15: Video S3**. Video of 3-week ahead forecast and actual distribution of dengue incidence in 2016. (MP4 1148 kb)



**Additional file 16: Video S4**. Video of 4-week ahead forecast and actual distribution of dengue incidence in 2016. (MP4 1125 kb)



**Additional file 17: Video S5**. Video of 5-week ahead forecast and actual distribution of dengue incidence in 2016. (MP4 1107 kb)



**Additional file 18: Video S6**. Video of 6-week ahead forecast and actual distribution of dengue incidence in 2016. (MP4 1097 kb)



**Additional file 19: Video S7**. Video of 7-week ahead forecast and actual distribution of dengue incidence in 2016. (MP4 1071 kb)



**Additional file 20: Video S8**. Video of 8-week ahead forecast and actual distribution of dengue incidence in 2016. (MP4 1057 kb)



**Additional file 21: Video S9**. Video of 9-week ahead forecast and actual distribution of dengue incidence in 2016. (MP4 1037 kb)



**Additional file 22: Video S10**. Video of 10-week ahead forecast and actual distribution of dengue incidence in 2016. (MP4 1019 kb)



**Additional file 23: Video S11**. Video of 11-week ahead forecast and actual distribution of dengue incidence in 2016. (MP4 1008 kb)



**Additional file 24: Video S12**. Video of 12-week ahead forecast and actual distribution of dengue incidence in 2016. (MP4 992 kb)


**Fig. 4:**
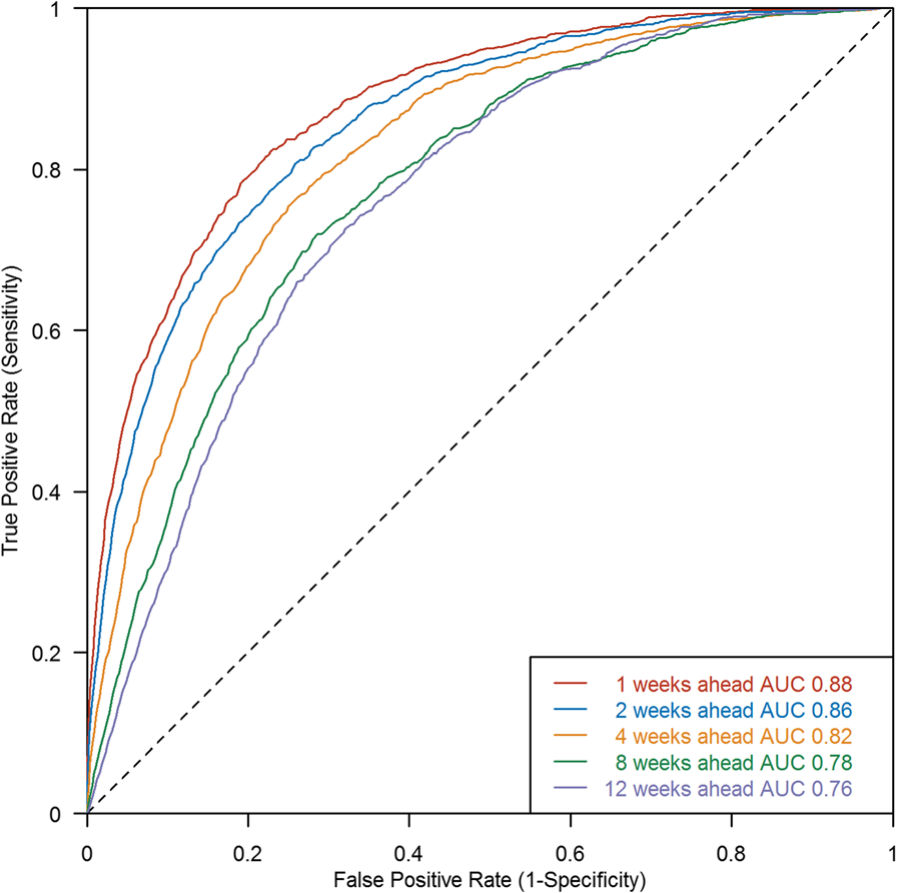
Aggregate ROC curves for forecast windows at 1, 2, 4, 8 and 12 weeks for classification of actual high- and low-risk neighbourhoods, aggregated over out-of-sample forecasts in 2016. The corresponding AUC values are marked. Because the bootstrap confidence intervals are very narrow, only average ROC curves are presented on this graph. AUC area under the ROC curve, ROC receiver operating characteristic

The quality of the spatial predictive algorithm as a classifier is measured by ROC curves and the respective AUCs. ROC curves for prediction windows at 1, 2, 4, 8 and 12 weeks are presented in Fig. 4 (bootstrap confidence intervals are very narrow and are not shown in the figure). All AUCs at forecast windows up to 12 weeks are above 0.75 and within 5 weeks, AUCs are above 0.80, indicating adequate performance in attributing neighbourhoods to be at high risk of imminent or ongoing transmission. The baseline AUC that uses the average of all past years’ cases as the prediction for the out-of-sample forecast is derived to be 0.78, which is better than guessing (i.e. the AUC is greater than 0.5) but which demonstrates that there are substantial gains in short-term predictive performance resulting from using updated data streams within our framework. Predictions for 6 weeks ahead and beyond revert to baseline risk.

**Fig. 5:**
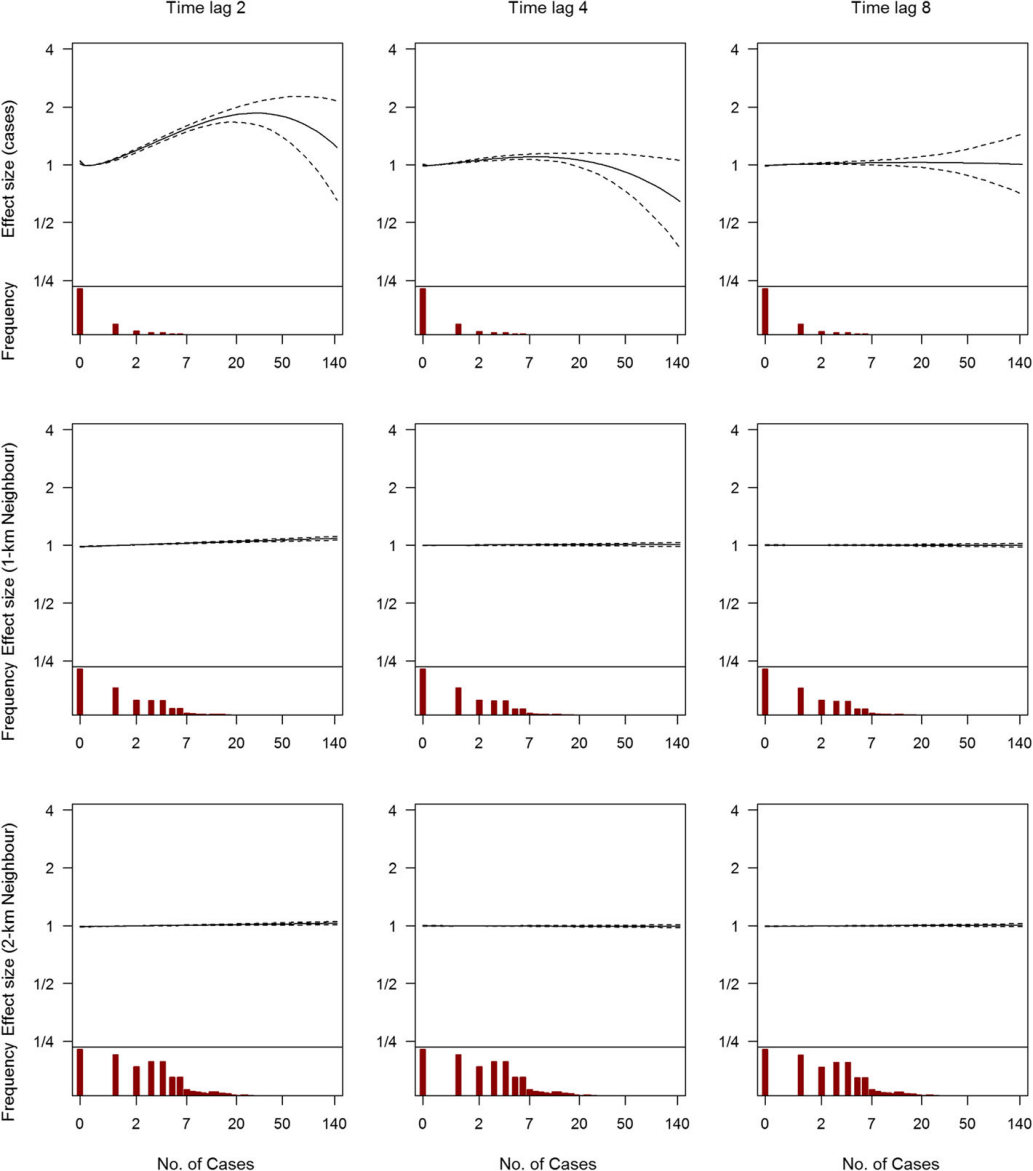
Histogram of the distribution and effect size on 1-week ahead forecast of dengue cases per neighbourhood. Recent case counts in neighbourhoods and total number of cases in the immediate vicinity are shown, at three time lags (2, 4 and 8). Histograms of the distribution are shown in the lower panes. The effects of covariates compared to the mean for that covariate are shown in the upper panes. Confidence intervals were derived using bootstrap sampling and are 95% equal tailed intervals

**Fig. 6:**
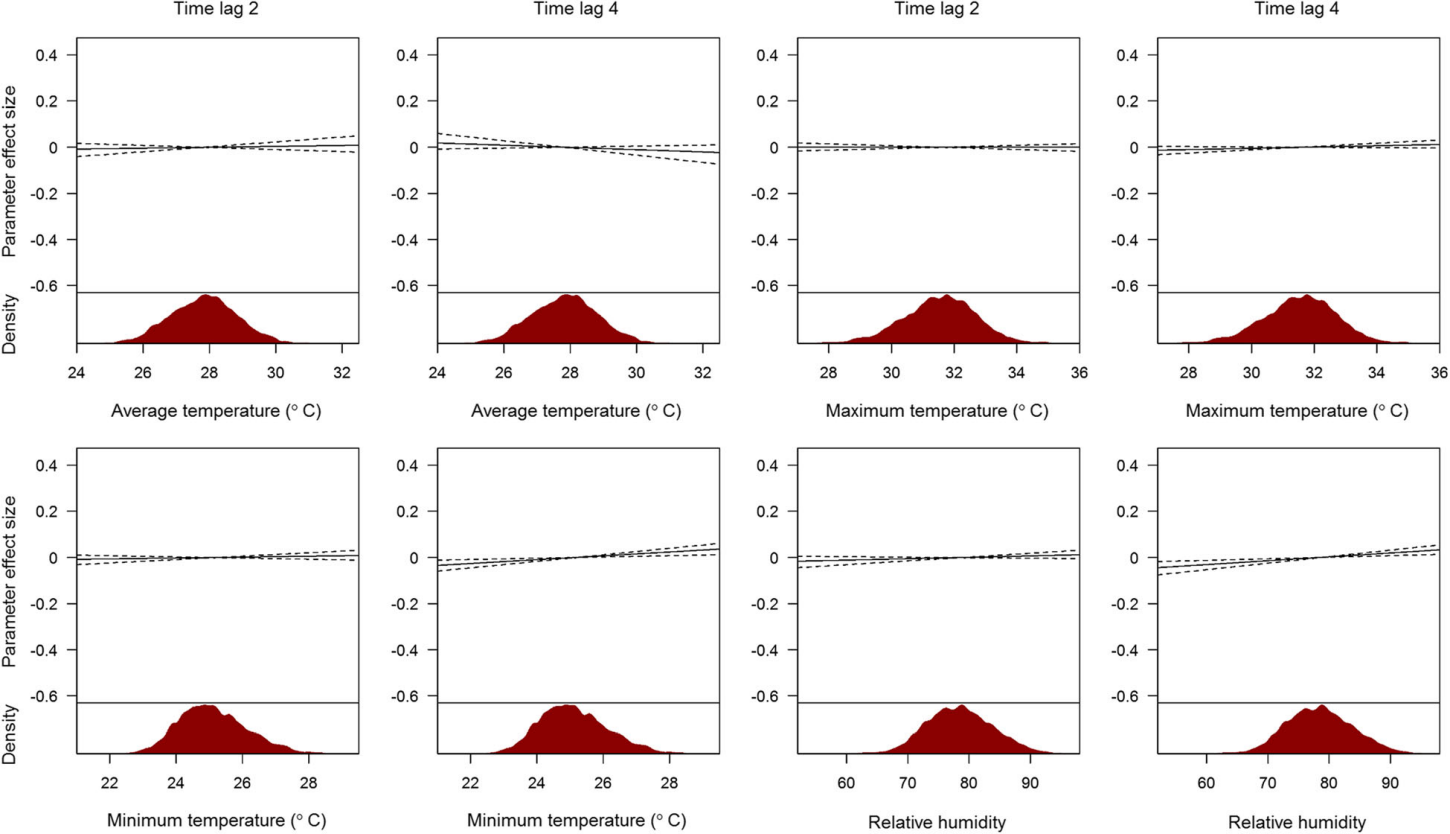
Distribution of climatic parameter and parameter effect in excess of the mean effect at two different time lags (2 and 4) for 1-week ahead forecasts. Upper panes show the effect and lower panes show the distribution of parameters. Confidence intervals were derived using bootstrap sampling

**Fig. 7:**
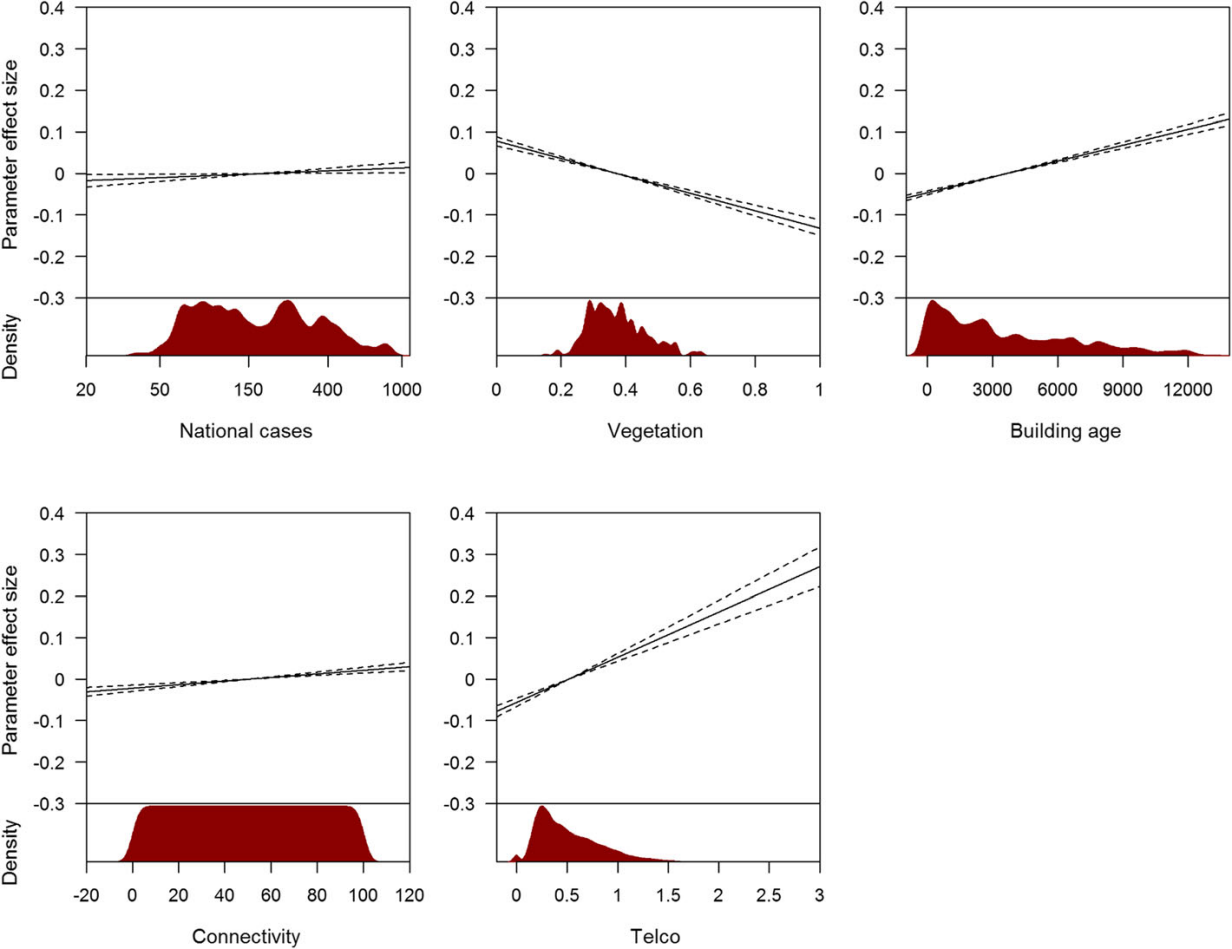
Distribution of parameters without time lags and parameter effect in excess of the mean effect. Upper panes show the effect and lower panes show the distribution of the parameters. Confidence intervals were derived using bootstrap sampling

The effect of risk factors on local dengue risk are shown in Figs. 5, 6 and 7. Figure 5 shows the effects of case counts within the neighbourhood and in proximate neighbourhoods for the 1-week ahead forecast model at three different time lags. The number of cases in a neighbourhood has a larger effect over short time lags compared to longer time lags, while the number of proximate cases has an effect size close to 0 at all lags. Although the relationship can be non-linear through the polynomial terms, the estimated effect is approximately linear. Climatic variables and their effects are shown in Fig. 6 (at time lags 2 and 4 for the 1-week ahead forecast). Maximum temperature, minimum temperature and relative humidity had a larger effect at longer time lags than the week immediately preceding the prediction, but relative to incidence, the effect is negligible. Figure 7 shows the effects of parameters without time lags. As expected, an increasing number of national weekly cases, less greenery, older buildings, greater connectivity to other areas and more incoming travellers to the area implied more cases. These parameters generally had a bigger effect than climatic variables, after adjusting for incidence and all other independent variables in the model. For each forecast window, the probability of each parameter being included in the final model, the estimated parameter coefficient and respective confidence interval are shown in Additional file 26: Tables S3 to S14 based on 1000 bootstrap samples. Incidence and neighbouring incidence at shorter lags were more likely to be included in the final model while climatic variables had a relatively smaller probability of being included and a smaller effect size.

**Fig. 8:**
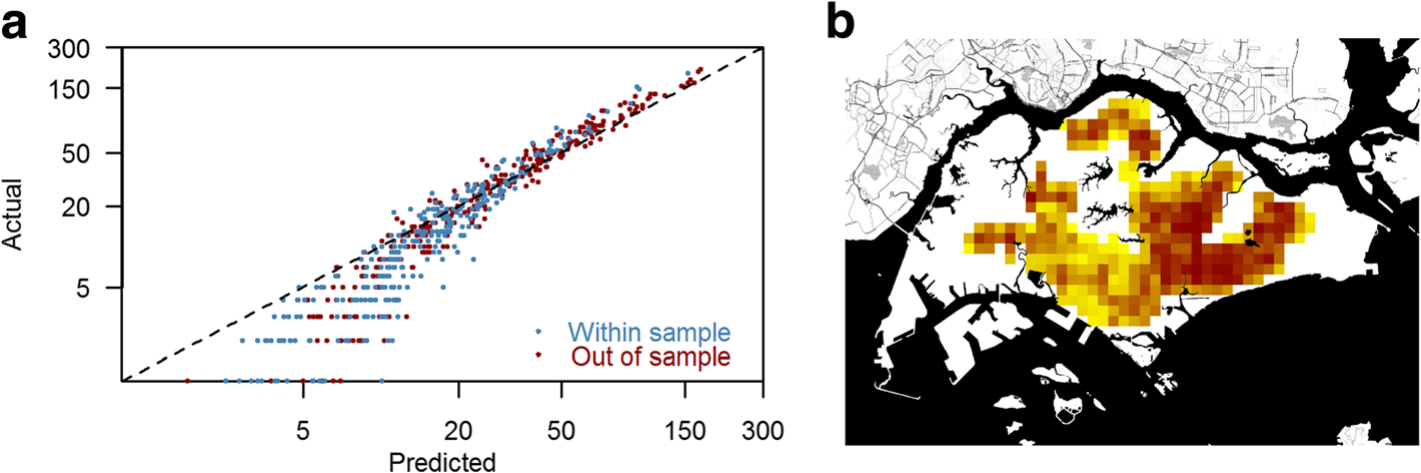
Comparisons of forecast and actual scenario for 1-week ahead forecast model. **a** Actual and predicted yearly total number of cases for all neighbourhoods for both within-sample prediction (blue dots) and out-of-sample prediction (dark red dots). **b** Average risk over all prediction points (both within-sample and out-of-sample) for the 1-week ahead forecast

An overall view of the 1-week ahead prediction model is shown in Fig. 8 (summaries for other all other forecast windows are shown in Additional file 27: Figure S11, Additional file 28: Figure S12, Additional file 29: Figure S13, Additional file 30: Figure S14, Additional file 31: Figure S15, Additional file 32: Figure S16, Additional file 33: Figure S17, Additional file 34: Figure S18, Additional file 35: Figure S19, Additional file 36: Figure S20 and Additional file 37: Figure S21). Panels Fig. 8(a) show the yearly sum of the 1-week ahead predicted number of cases and actual observed number of cases in all neighbourhoods. The relative sizes of the discrepancies were generally larger for smaller numbers, where accuracy may be less important, but the majority of predictions were accurate. Panels Fig. 8(b) show the average risk over all prediction points for the 1-week ahead forecast. Neighbourhoods in the east of Singapore had a higher risk than the other regions.

## Discussion

In Singapore, the average annual economic impact of dengue has been estimated to be around US$100 million, of which 42–59% is attributable to the cost of control [11]. Routine surveillance identifies residential and workplace addresses for all notified cases, which leads to dengue clusters being identified, namely localities with putatively active transmission where NEA’s vector control intervention is targeted [48]. A cluster is formed when two or more cases have onset within 14 days and are located within 150 m of each other based on the addresses as well as movement history. Three alert levels, depending on the number of cases in the cluster, lead to efforts to mobilise the community to check their premises for mosquito breeding, and guide the extent of NEA’s vector control intervention. However, these alert levels are based on current or recent infections, rather than the areas most likely to see further transmission. Being able to focus control on where new cases are most likely to arise, rather than where they are currently, could allow preemptive mitigation and potentially yield greater efficiencies and reduce costs accordingly.

Thus, in this study we developed a novel method to forecast spatial risk within an urban environment at a neighbourhood resolution up to 3 months in advance, using a LASSO-based prediction model. The method gave rather accurate forecasts (AUCs > 0.8 within the next month), with a high correlation with the subsequent incidence data. However, for longer forecast windows, the risk reverted to a baseline risk profile for the neighbourhood. By implementing it as part of our standing vector control programme, the spatio-temporal prediction model can potentially change the current dengue control paradigm into a dengue prevention approach by forecasting dengue risk at a finer resolution in the urbanised environments in which the dengue vectors proliferate. This would allow targeted public health control measures that would use resources most efficiently. The system was robust to changes in the baseline incidence over time (illustrated in Fig. 1a), as demonstrated in the high correlation between observed and predicted incidence (Fig. 8a). As such, secular changes in the detection rates due to better diagnosis or in incidence due to changes in immunity or dominant serotypes may not matter unless the change is large.

This approach can readily be automated to run on routinely collected notification data, but the accuracy of the prediction is dependent on the timeliness at which notification data become available and the accuracy of such data. The approach does not require that all infections be notified or confirmed by a lab—the low rate of symptomatic dengue presentation is well known [49]—as long as the rate remains relatively stable over space and time. The training dataset used in this modelling framework, however, may need to be updated regularly. In the current approach, the performance for 2016 (the data for which were not used in training) was good (AUC above 0.75 for all forecast windows), and so we recommend retraining the algorithm every year.

Through regular evaluation of all the parameter effect sizes, variables with a constant minimal effect in the forecast model may be eliminated, reducing the cost of obtaining them. Other potential parameters may be added to the model in a similar way. The frequent modification of the model to allow additional data streams to be incorporated will ensure the model continues to enjoy high predictive performance.

We expect that the same method can be adapted to other urban and even rural areas, though in the latter, the grid size determining neighbourhoods may need to be adjusted. We used a regular grid, but the framework lends itself to other tessellations, for instance, administrative boundaries. We anticipate that such regional or neighbourhood-level forecasts will have improved accuracy and utility than predictions of aggregate national-level data streams.

There are several limitations of the approach outlined herein. The forecast is phenomenological rather than mechanistic, and as such may break down in the presence of changes to the underlying epidemic process and changes to interventions. A previous non-spatial forecast (described in Ref. [24]) struggled to reproduce the magnitude of the record-breaking outbreak of 2013, for instance, although it was able to herald the timing of the outbreak in advance. Fundamental changes, such as vaccination or the introduction of a new serotype to the population, may require the retraining of the algorithm if the accuracy is not to be deleteriously affected. Further mechanistic modelling could be valuable in providing additional insight into the spatial structure of dengue transmission in Singapore, if challenges about non-notified infections and the paucity of data on historic exposures to each serotype could be overcome. The multiple lags and forecast windows allows highly predictive combinations of variables to be selected, but have the effect of obscuring relationships, and as a result, the approach is not suitable for identifying why particular neighbourhoods are predicted to be at risk of future or imminent transmission. The most important limitation to the work is its high reliance on a rich dataset of georeferenced case identifications being available in near real time. This is possible in Singapore’s comprehensive case notification system but may be less feasible in jurisdictions that do not enjoy Singapore’s small size and the clear demarcation of the city population. The effectiveness of vector control measures based on the forecast is not evaluated in the current model, and to predict the impact would require additional data streams that capture the details of the ongoing vector control efforts. This would be an avenue for further work.

## Conclusions

In conclusion, this report demonstrates that spatially resolved forecasts of geographically structured diseases like dengue can be obtained at a neighbourhood-level in highly urban environments at a precision that is suitable for guiding control efforts.

## Additional files

Additional file 1: Supplementary information. (DOCX 19 kb)

Additional file 2: **Figure S1.** Demonstration of the two tiers of neighbouring cells in the study. (PNG 338 kb)

Additional file 3: **Table S1.** Groups of variables and their respective types that are included in the LASSO model. (DOCX 11 kb)

Additional file 4: **Figure S2.**Temporal and spatial average of the weekly number of cases in all grid cells from 2010 to 2016. (PNG 200 kb)

Additional file 5: **Figure S3.** Temporal and spatial average of the sum of the weekly number of cases in all first-tier neighbouring cells (within 1 km) from 2010 to 2016. (PNG 199 kb)

Additional file 6: **Figure S4.** Temporal and spatial average of the sum of the weekly number of cases in all second-tier neighbouring cells (between 1 km and 2 km) from 2010 to 2016. (PNG 198 kb)

Additional file 7: **Figure S5.** Temporal and spatial average of the average temperature in all grid cells from 2010 to 2016. (PNG 202 kb)

Additional file 8: **Figure S6.** Temporal and spatial average of the maximum temperature in all grid cells from 2010 to 2016. (PNG 200 kb)

Additional file 9: **Figure S7.** Temporal and spatial average of the minimum temperature in all grid cells from 2010 to 2016. (PNG 201 kb)

Additional file 10: **Figure S8.** Temporal and spatial average of the average humidity in all grid cells from 2010 to 2016. (PNG 201 kb)

Additional file 11: **Figure S9.** Temporal and spatial average of the movement of incoming incidences in all grid cells from 2010 to 2016. (PNG 198 kb)

Additional file 12: **Figure S10.** Temporal and spatial average of the average building age in all grid cells from 2010 to 2016. (PNG 197 kb)

Additional file 25: **Table S2.** Average Matthew’s correlation coefficient for all 12 forecast windows at 14 different cut-offs. Cut-off are set at different levels so that different percentages of the cells are classified as higher risk areas. (DOCX 12 kb)

Additional file 26: **Table S3.** Summary of parameters included in LASSO forecast model for the 1-week ahead forecast. **Table S4.** Summary of parameters included in LASSO forecast model for the 2-week ahead forecast. **Table S5.** Summary of parameters included in LASSO forecast model for the 3-week ahead forecast. **Table S6.** Summary of parameters included in LASSO forecast model for the 4-week ahead forecast. **Table S7.** Summary of parameters included in LASSO forecast model for the 5-week ahead forecast. **Table S8.** Summary of parameters included in LASSO forecast model for the 6-week ahead forecast. **Table S9.** Summary of parameters included in LASSO forecast model for the 7-week ahead forecast. **Table S10.** Summary of parameters included in LASSO forecast model for the 8-week ahead forecast. **Table S11.** Summary of parameters included in LASSO forecast model for the 9-week ahead forecast. **Table S12.** Summary of parameters included in LASSO forecast model for the 10-week ahead forecast. **Table S13.** Summary of parameters included in LASSO forecast model for the 11-week ahead forecast. **Table S14.** Summary of parameters included in LASSO forecast model for the 12-week ahead forecast. (DOCX 37 kb)

Additional file 27: **Figure S11.** Comparisons of forecast and actual scenario for the 2-week ahead forecast model. **a** Actual and predicted yearly total number of cases for all neighbourhoods for both within-sample prediction (blue dots) and out-of-sample prediction (dark red dots). **b** Average risk over all prediction points (both within-sample and out-of-sample) for the 1-week ahead forecast. (PNG 189 kb)

Additional file 28: **Figure S12.** Comparisons of forecast and actual scenario for the 3-week ahead forecast model. **a** Actual and predicted yearly total number of cases for all neighbourhoods for both within-sample prediction (blue dots) and out-of-sample prediction (dark red dots). **b** Average risk over all prediction points (both within-sample and out-of-sample) for the 1-week ahead forecast. (PNG 189 kb)

Additional file 29: **Figure S13.** Comparisons of forecast and actual scenario for the 4-week ahead forecast model. **a** Actual and predicted yearly total number of cases for all neighbourhoods for both within-sample prediction (blue dots) and out-of-sample prediction (dark red dots). **b** Average risk over all prediction points (both within-sample and out-of-sample) for the 1-week ahead forecast. (PNG 189 kb)

Additional file 30: **Figure S14.** Comparisons of forecast and actual scenario for the 5-week ahead forecast model. **a** Actual and predicted yearly total number of cases for all neighbourhoods for both within-sample prediction (blue dots) and out-of-sample prediction (dark red dots). **b** Average risk over all prediction points (both within-sample and out-of-sample) for the 1-week ahead forecast. (PNG 190 kb)

Additional file 31: **Figure S15.** Comparisons of forecast and actual scenario for the 6-week ahead forecast model. **a** Actual and predicted yearly total number of cases for all neighbourhoods for both within-sample prediction (blue dots) and out-of-sample prediction (dark red dots). **b** Average risk over all prediction points (both within-sample and out-of-sample) for the 1-week ahead forecast. (PNG 190 kb)

Additional file 32: **Figure S16.** Comparisons of forecast and actual scenario for the 7-week ahead forecast model. **a** Actual and predicted yearly total number of cases for all neighbourhoods for both within-sample prediction (blue dots) and out-of-sample prediction (dark red dots). **b** Average risk over all prediction points (both within-sample and out-of-sample) for the 1-week ahead forecast. (PNG 190 kb)

Additional file 33: **Figure S17.** Comparisons of forecast and actual scenario for the 8-week ahead forecast model. **a** Actual and predicted yearly total number of cases for all neighbourhoods for both within-sample prediction (blue dots) and out-of-sample prediction (dark red dots). **b** Average risk over all prediction points (both within-sample and out-of-sample) for the 1-week ahead forecast. (PNG 190 kb)

Additional file 34: **Figure S18.** Comparisons of forecast and actual scenario for the 9-week ahead forecast model. **a** Actual and predicted yearly total number of cases for all neighbourhoods for both within-sample prediction (blue dots) and out-of-sample prediction (dark red dots). **b** Average risk over all prediction points (both within-sample and out-of-sample) for the 1-week ahead forecast. (PNG 190 kb)

Additional file 35: **Figure S19.** Comparisons of forecast and actual scenario for the 10-week ahead forecast model. **a** Actual and predicted yearly total number of cases for all neighbourhoods for both within-sample prediction (blue dots) and out-of-sample prediction (dark red dots). **b** Average risk over all prediction points (both within-sample and out-of-sample) for the 1-week ahead forecast. (PNG 190 kb)

Additional file 36: **Figure S20.** Comparisons of forecast and actual scenario for the 11-week ahead forecast model. **a** Actual and predicted yearly total number of cases for all neighbourhoods for both within-sample prediction (blue dots) and out-of-sample prediction (dark red dots). **b** Average risk over all prediction points (both within-sample and out-of-sample) for the 1-week ahead forecast. (PNG 190 kb)

Additional file 37: **Figure S21.** Comparisons of forecast and actual scenario for the 12-week ahead forecast model. **a** Actual and predicted yearly total number of cases for all neighbourhoods for both within-sample prediction (blue dots) and out-of-sample prediction (dark red dots). **b** Average risk over all prediction points (both within-sample and out-of-sample) for the 1-week ahead forecast. (PNG 190 kb)

## Abbreviations

AUC: Area under the ROC curve
DENV: Dengue virus
LASSO: Least absolute shrinkage and selection operator
NDVI: Normalised difference vegetation index
NEA: National Environment Agency
ROC: Receiver operating characteristic
Telco: Mobile telephone company
