# Supplementary material for: Neighbourhood level real-time forecasting of dengue cases in tropical urban Singapore
Source: BMC Med. 2018 Aug 6;16:129. doi: 10.1186/s12916-018-1108-5 (PMC6091171; doi:10.1186/s12916-018-1108-5)
Supplement: Supplementary file 13 — Supplementary information. (DOCX 19 kb) [file 12916_2018_1108_MOESM1_ESM.docx]

**Supplementary Information**

A detailed description of data related to movement data used in the study is listed in this supplementary information.

**Mobile telephony**

Movement patterns of mobile subscribers were derived by analysing their cell phones’ network activities among subscribers of Starhub Ltd, one of the three major mobile telephone companies (telcos) in Singapore. The mobile data comprises all the post-paid subscribers of StarHub Ltd, which constitutes approximately 30% coverage of total mobile subscribers in Singapore. GRID360 – a geo-location product developed by StarHub Ltd to anonymize and aggregate island-wide geolocation-related data onto a regular grid of size 165m for business purposes – was deployed by StarHub to detect dwelling points of individual subscribers and subsequently their origin and destination of travel by analysing their cell phones’ network activities. The GRID360 origin-destination engine detects a dwelling point (origin or destination) when the subscriber has stayed in a neighbourhood for 30 minutes or longer. The location data are generated when the following occur:

1. Whenever there are activities on the phone which engage a service of the telco, such as usage of mobile data, including of applications running in the background, calls or short message services.
2. Whenever the phone moves into another location area code.
3. Periodically, there is a location update (roughly once every 2 to 3 hours) if there is no activity or movement to another location area code.

These location-time data are then aggregated to yield the total number of subscribers traveling to and from each pair of grids. To this end, whenever an individual moved from a location in neighbourhood $i$ to neighbourhood $j$, spending longer than 30 minutes there, this was counted as a trip between the two neighbourhoods. The time threshold of 30 minutes was chosen to ensure that the individual was not just passing through the neighbourhood en route to another. From the set of such trips, we constructed an origin-destination connectivity matrix,

$\left[ \begin{matrix} n_{11} & \cdots& n_{1N} \\ \vdots& \ddots& \vdots\\ n_{N1} & \cdots& n_{NN} \end{matrix} \right]$ ,

where $n_{ij}$ denotes the total number of subscribers traveling from grid $j$ to grid $i$.

We then derived a variable, *connectivity-weighted transmission potential*, by weighting the cases with the telco data using the formula:

$$x_{it}=\frac{\sum_{j=1}^{N} n_{ij}C_{jt}}{\sum_{j=1}^{N} n_{ij}}$$

where $x_{it}$ is the weighted number of dengue cases from all other grid cells, weighted by their connectivity, for grid $i$ at time $t$; and $C_{jt}$ is the total number of dengue cases in grid $j$ at time $t$. Note that although the connectivity measurement, $n_{ij}$, is spatial but non-temporal, the derived variable, *connectivity-weighted transmission potential*, is spatio-temporal through its relationship with the number of cases per cell at each time point.

**Public transport (EZlink) cards**

Aggregate movement patterns were extracted from a database of public transport card utilisations by the Future Cities Laboratory. The original data contain the time and location at which passengers on the bus, train or monorail systems enter and leave the network, together with the route used. Locations were identified with the grid cell containing the station or bus stop, and the “connectivity” of each cell was determined by summing the number of trips originating from, or terminating at, each cell over a one-month period. Cells were then percentile ranked from most (100) to least (0) connected. The ranked connectivity differs from the mobile telephony data by capturing transits (for instance, from one bus to another, or from the train to a bus) rather than sojourns (due to for instance spending prolonged time in an office building) and thus potentially captures an alternative facet of mobility.
