# Supplementary figures and images for: Neighbourhood level real-time forecasting of dengue cases in tropical urban Singapore
Source: BMC Med. 2018 Aug 6;16:129. doi: 10.1186/s12916-018-1108-5 (PMC6091171; doi:10.1186/s12916-018-1108-5)

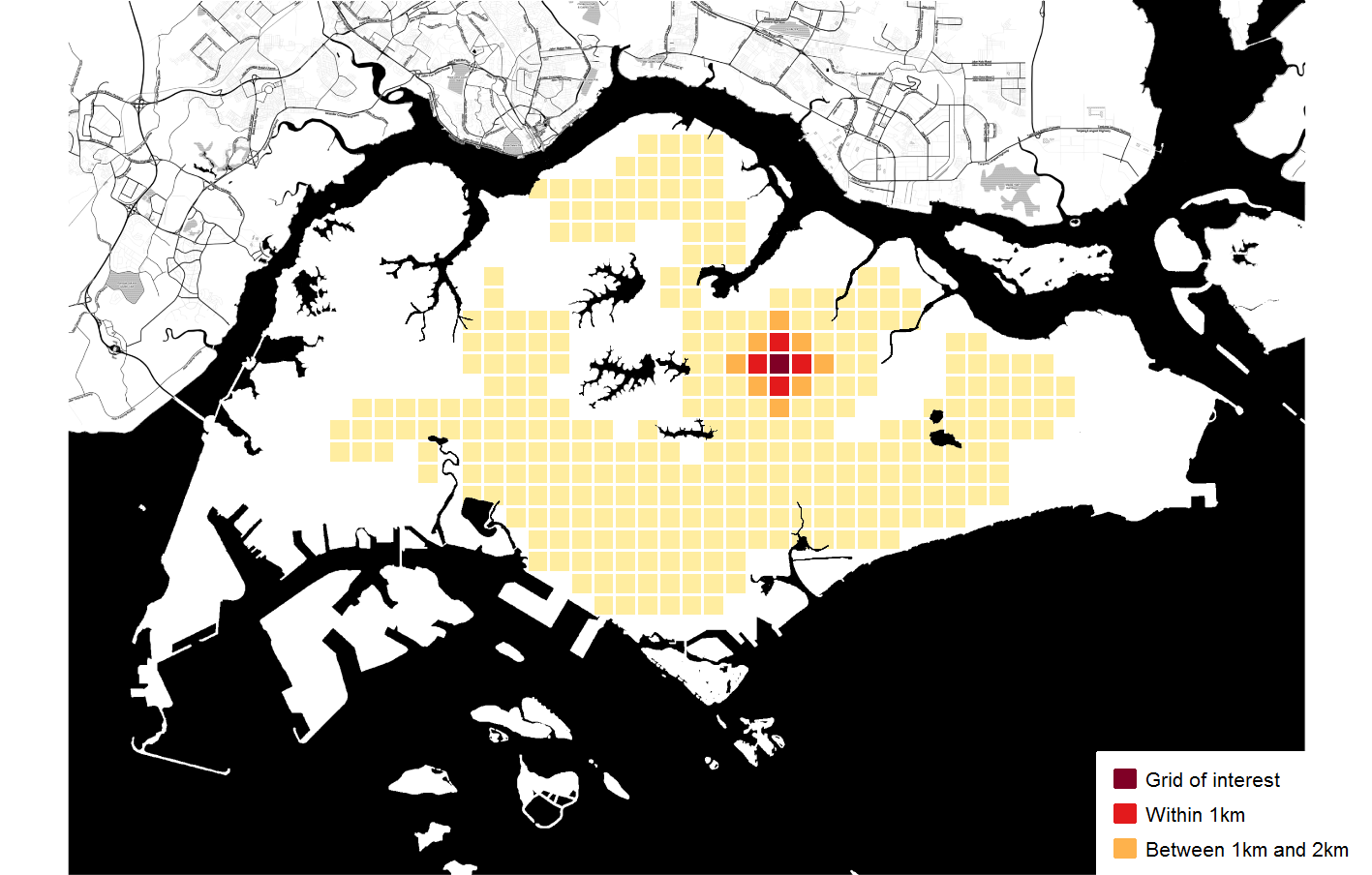

Supplement: Supplementary file 14 — Figure S1. Demonstration of the two tiers of neighbouring cells in the study. (PNG 338 kb) [file 12916_2018_1108_MOESM2_ESM.png]

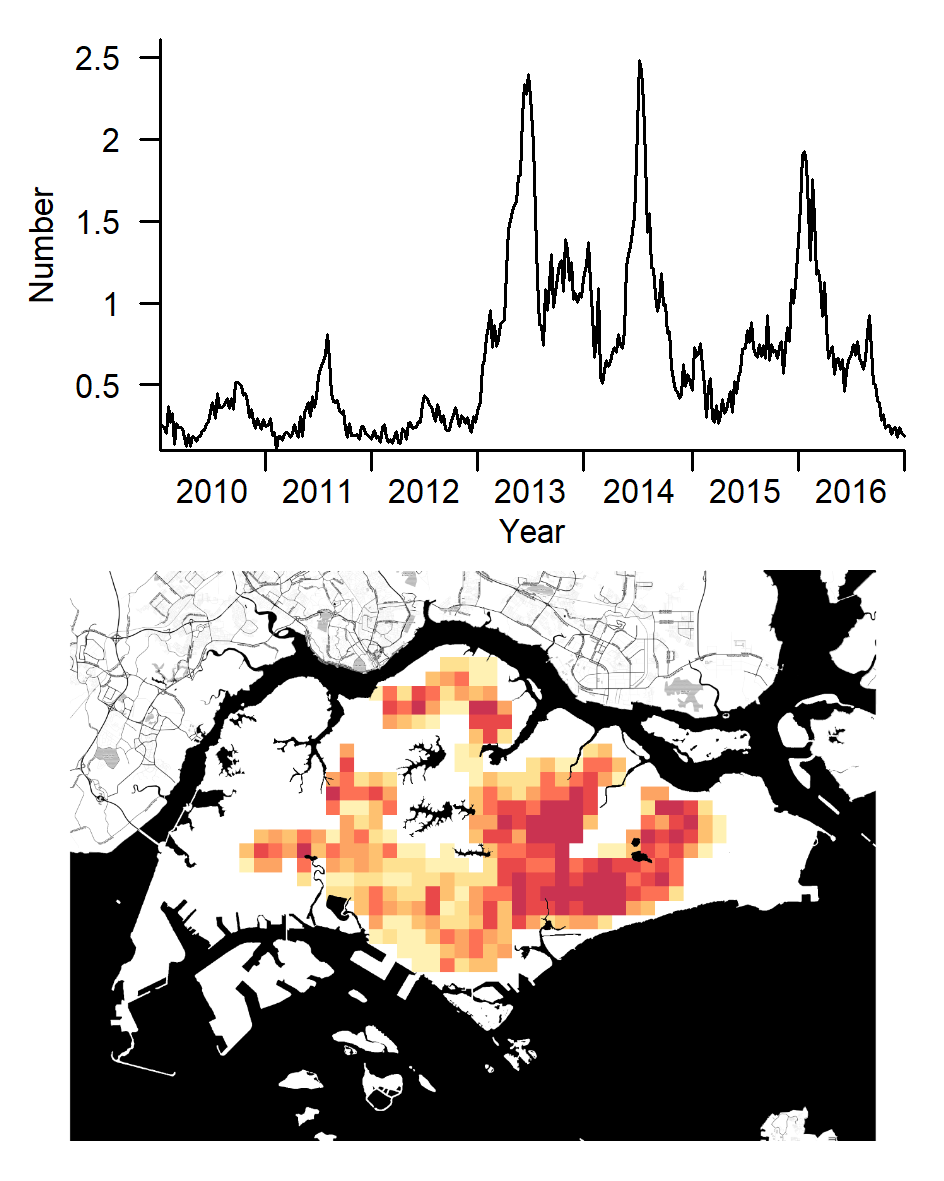

Supplement: Supplementary file 16 — Figure S2.Temporal and spatial average of the weekly number of cases in all grid cells from 2010 to 2016. (PNG 200 kb) [file 12916_2018_1108_MOESM4_ESM.png]

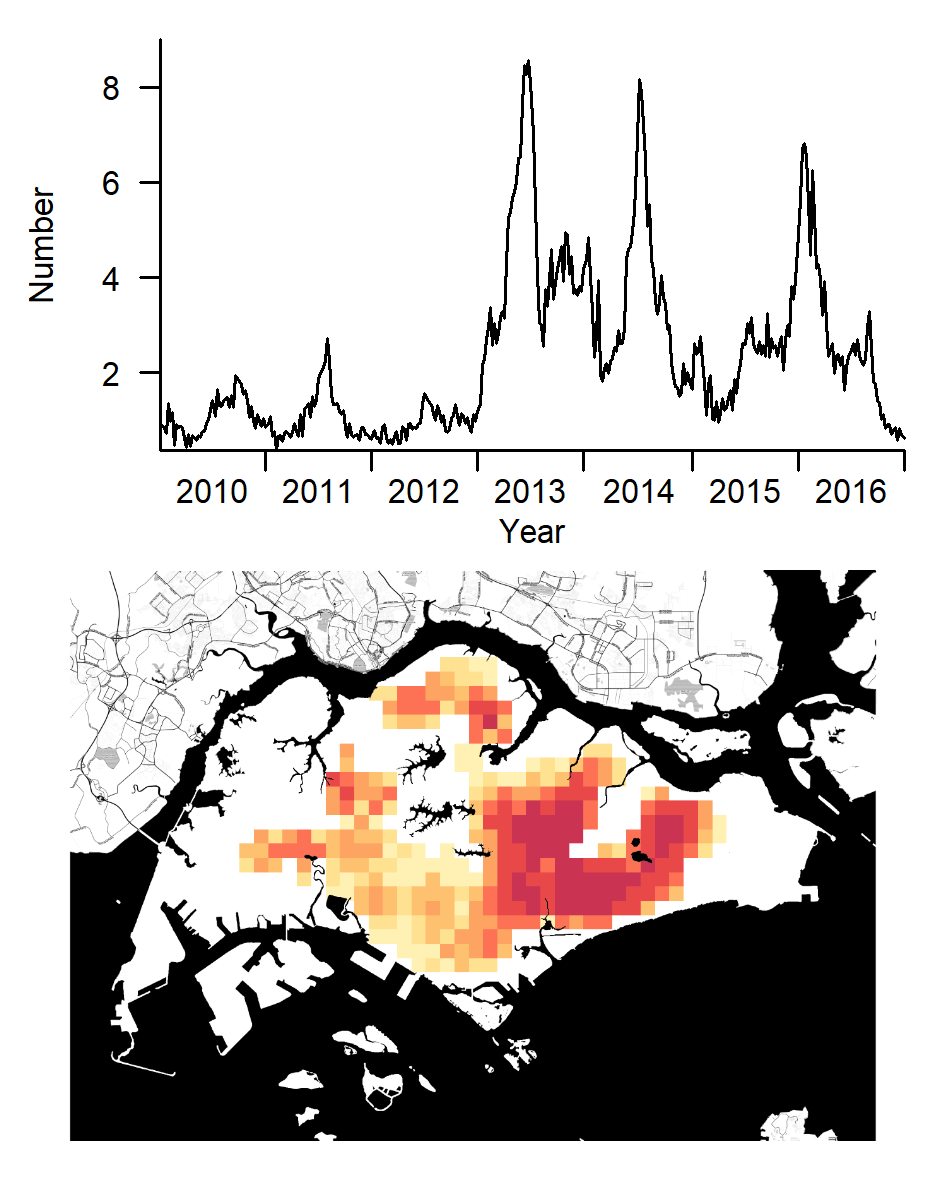

Supplement: Supplementary file 17 — Figure S3. Temporal and spatial average of the sum of the weekly number of cases in all first-tier neighbouring cells (within 1 km) from 2010 to 2016. (PNG 199 kb) [file 12916_2018_1108_MOESM5_ESM.png]

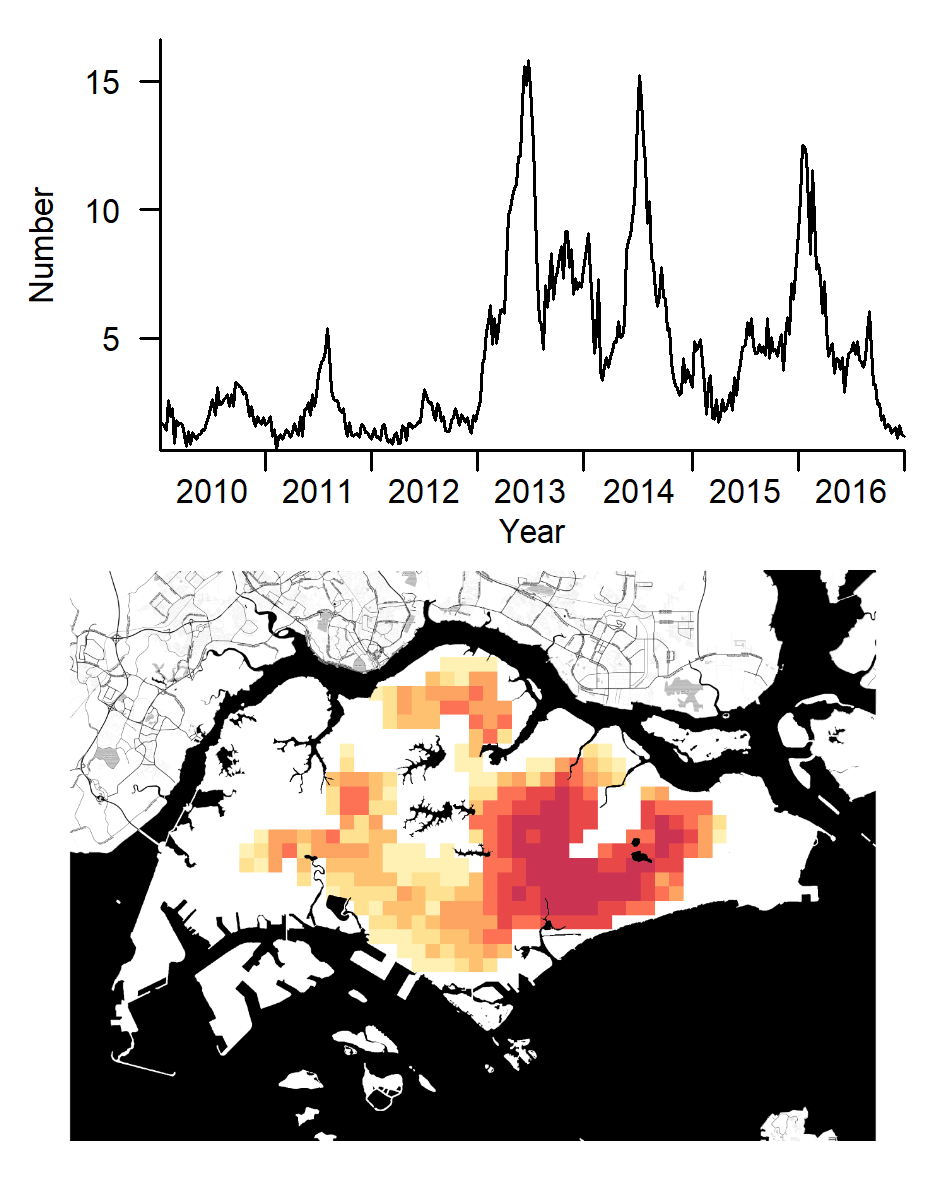

Supplement: Supplementary file 18 — Figure S4. Temporal and spatial average of the sum of the weekly number of cases in all second-tier neighbouring cells (between 1 km and 2 km) from 2010 to 2016. (PNG 198 kb) [file 12916_2018_1108_MOESM6_ESM.png]

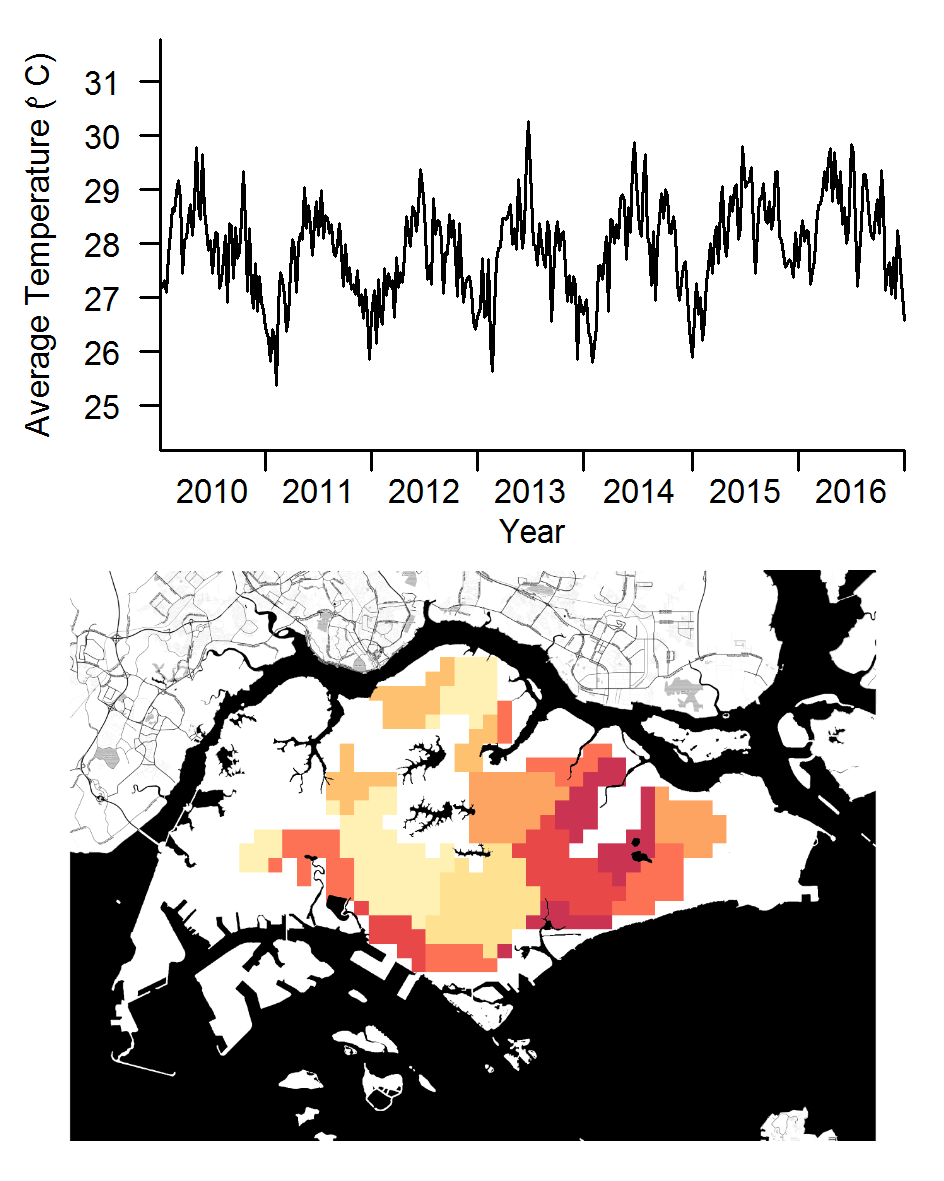

Supplement: Supplementary file 19 — Figure S5. Temporal and spatial average of the average temperature in all grid cells from 2010 to 2016. (PNG 202 kb) [file 12916_2018_1108_MOESM7_ESM.png]

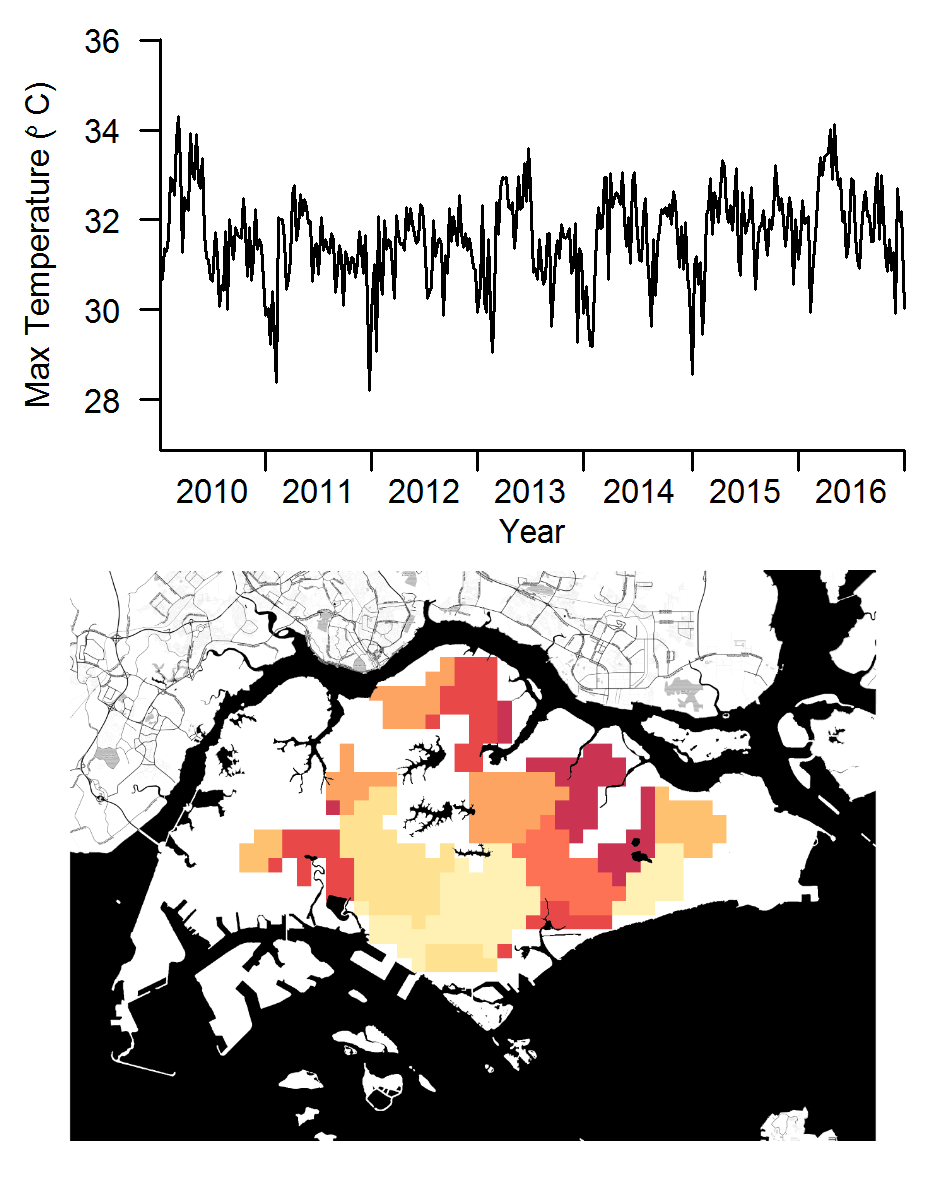

Supplement: Supplementary file 20 — Figure S6. Temporal and spatial average of the maximum temperature in all grid cells from 2010 to 2016. (PNG 200 kb) [file 12916_2018_1108_MOESM8_ESM.png]

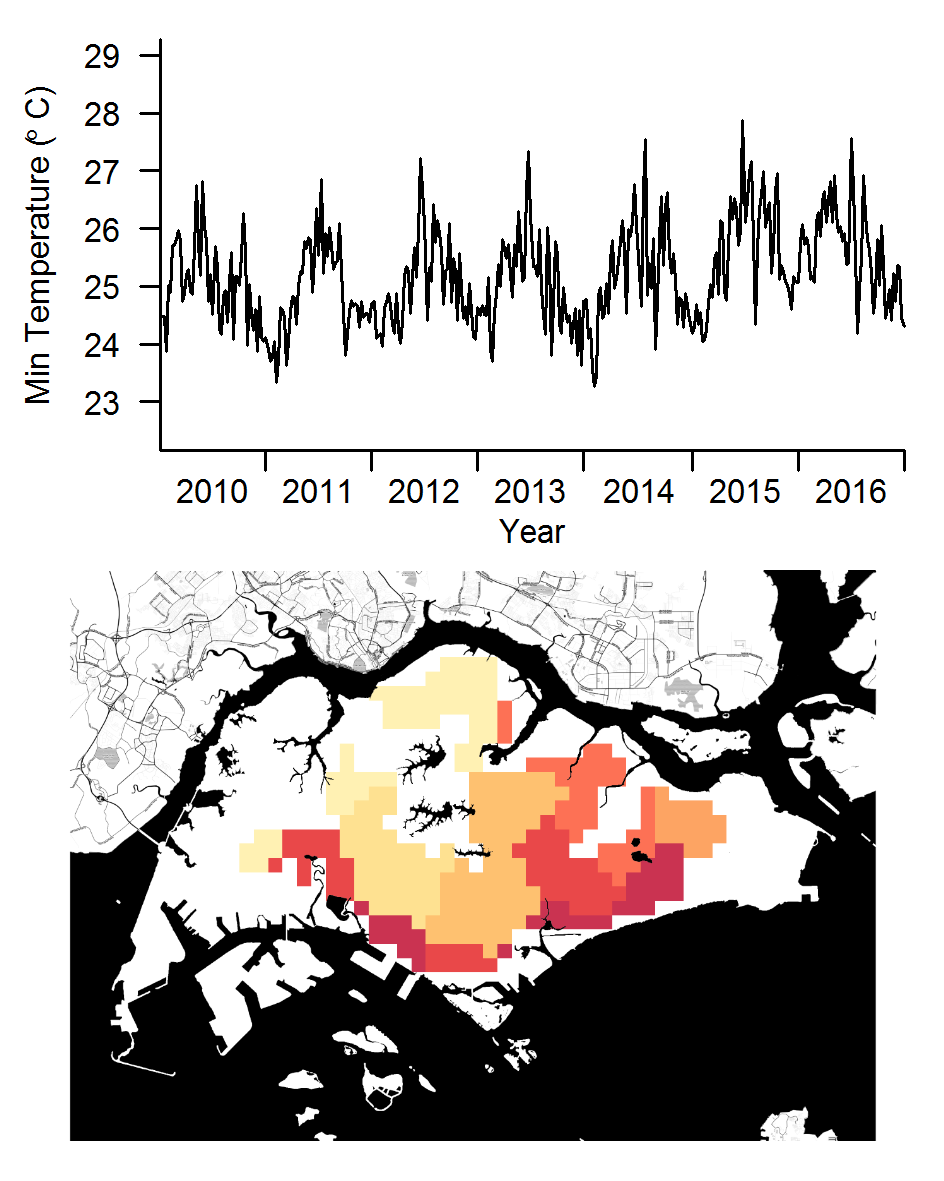

Supplement: Supplementary file 21 — Figure S7. Temporal and spatial average of the minimum temperature in all grid cells from 2010 to 2016. (PNG 201 kb) [file 12916_2018_1108_MOESM9_ESM.png]

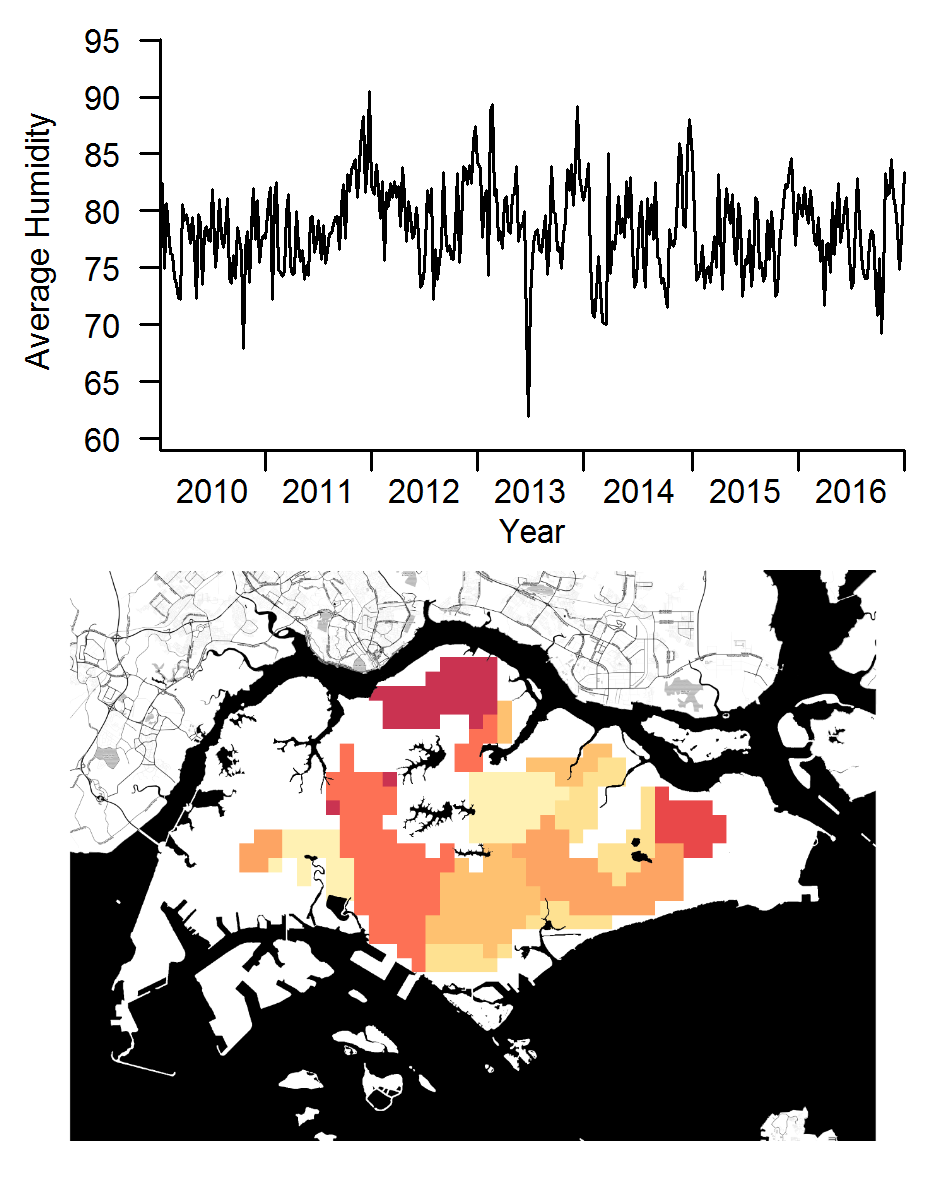

Supplement: Supplementary file 22 — Figure S8. Temporal and spatial average of the average humidity in all grid cells from 2010 to 2016. (PNG 201 kb) [file 12916_2018_1108_MOESM10_ESM.png]

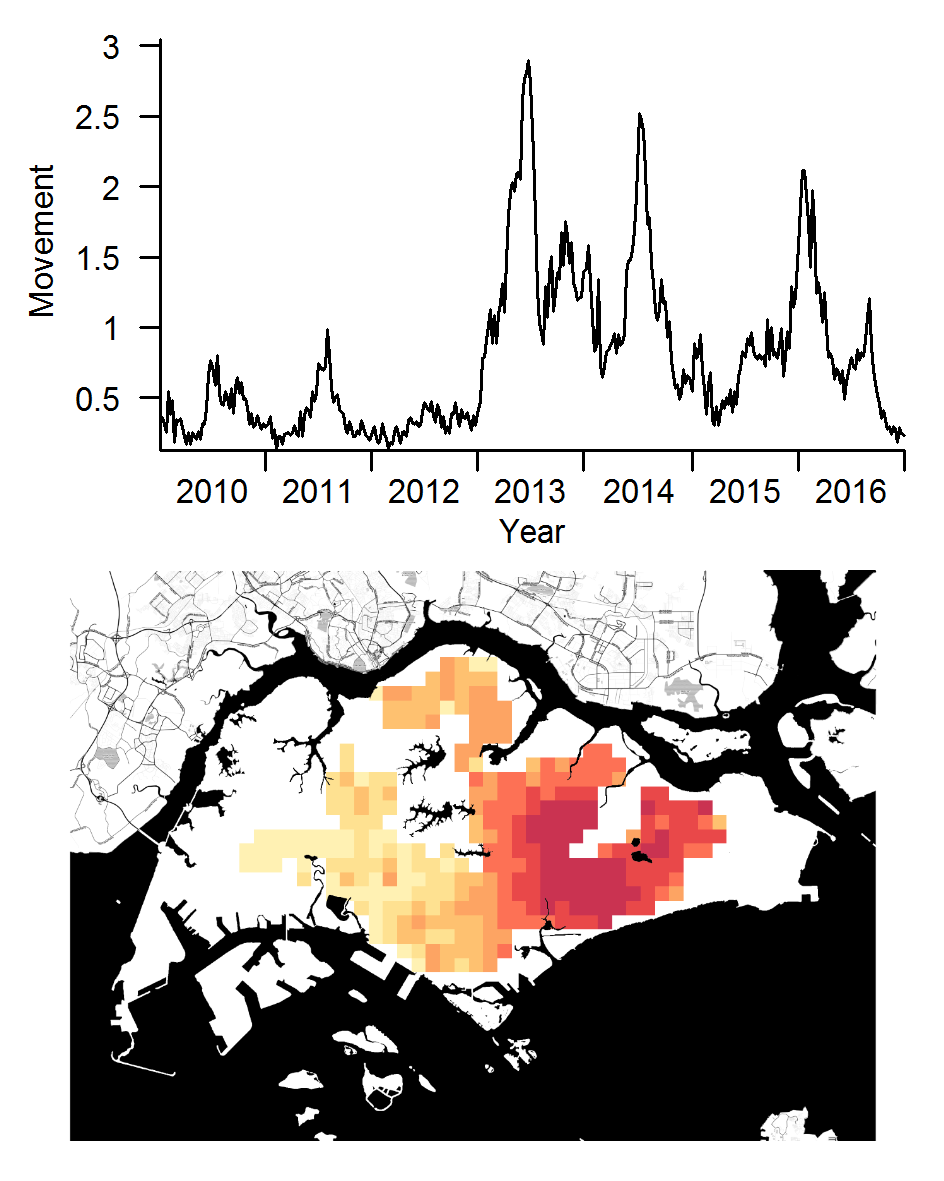

Supplement: Supplementary file 23 — Figure S9. Temporal and spatial average of the movement of incoming incidences in all grid cells from 2010 to 2016. (PNG 198 kb) [file 12916_2018_1108_MOESM11_ESM.png]

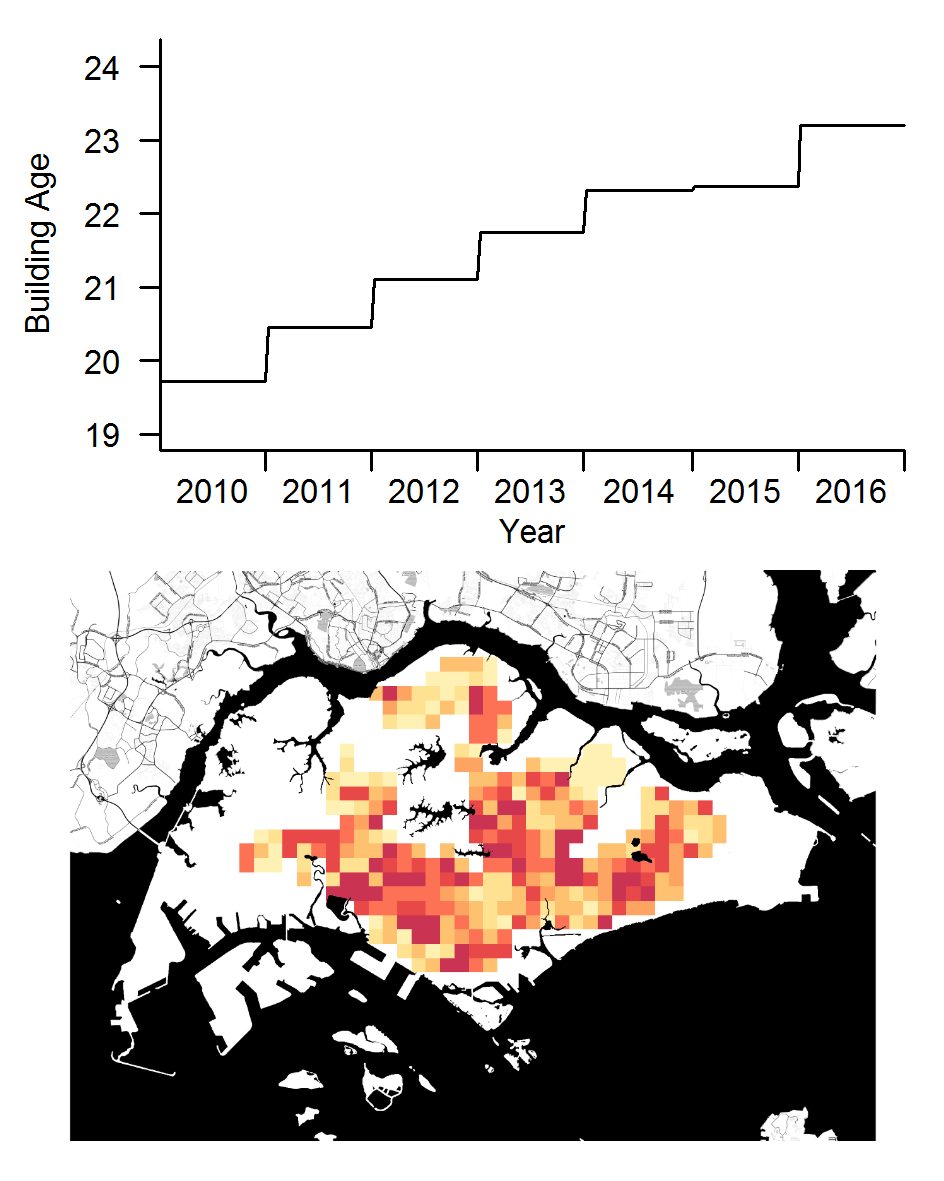

Supplement: Supplementary file 24 — Figure S10. Temporal and spatial average of the average building age in all grid cells from 2010 to 2016. (PNG 197 kb) [file 12916_2018_1108_MOESM12_ESM.png]

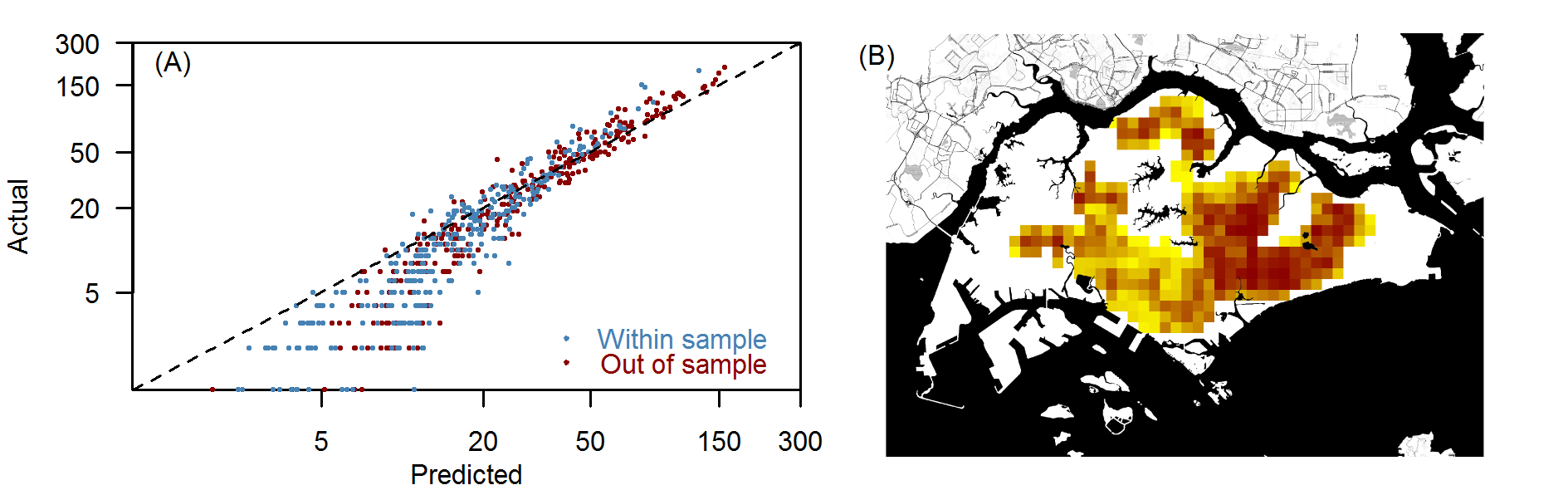

Supplement: Supplementary file 27 — Figure S11. Comparisons of forecast and actual scenario for the 2-week ahead forecast model. a Actual and predicted yearly total number of cases for all neighbourhoods for both within-sample prediction (blue dots) and out-of-sample prediction (dark red dots). b Average risk over all prediction points (both within-sample and out-of-sample) for the 1-week ahead forecast. (PNG 189 kb) [file 12916_2018_1108_MOESM27_ESM.png]

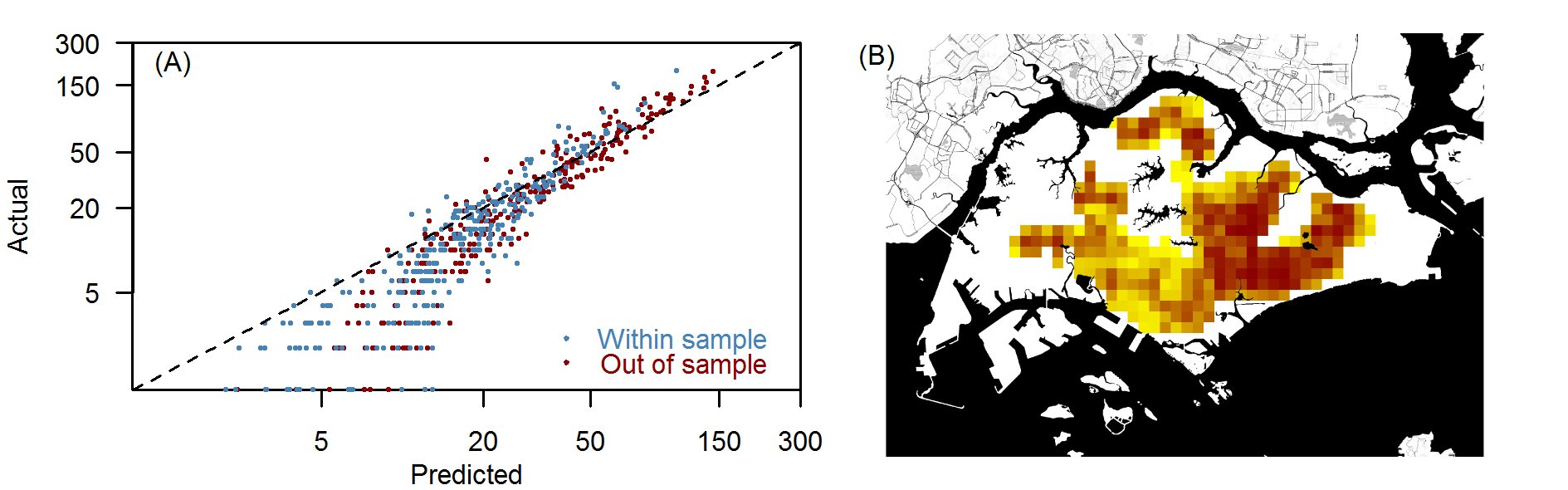

Supplement: Supplementary file 28 — Figure S12. Comparisons of forecast and actual scenario for the 3-week ahead forecast model. a Actual and predicted yearly total number of cases for all neighbourhoods for both within-sample prediction (blue dots) and out-of-sample prediction (dark red dots). b Average risk over all prediction points (both within-sample and out-of-sample) for the 1-week ahead forecast. (PNG 189 kb) [file 12916_2018_1108_MOESM28_ESM.png]

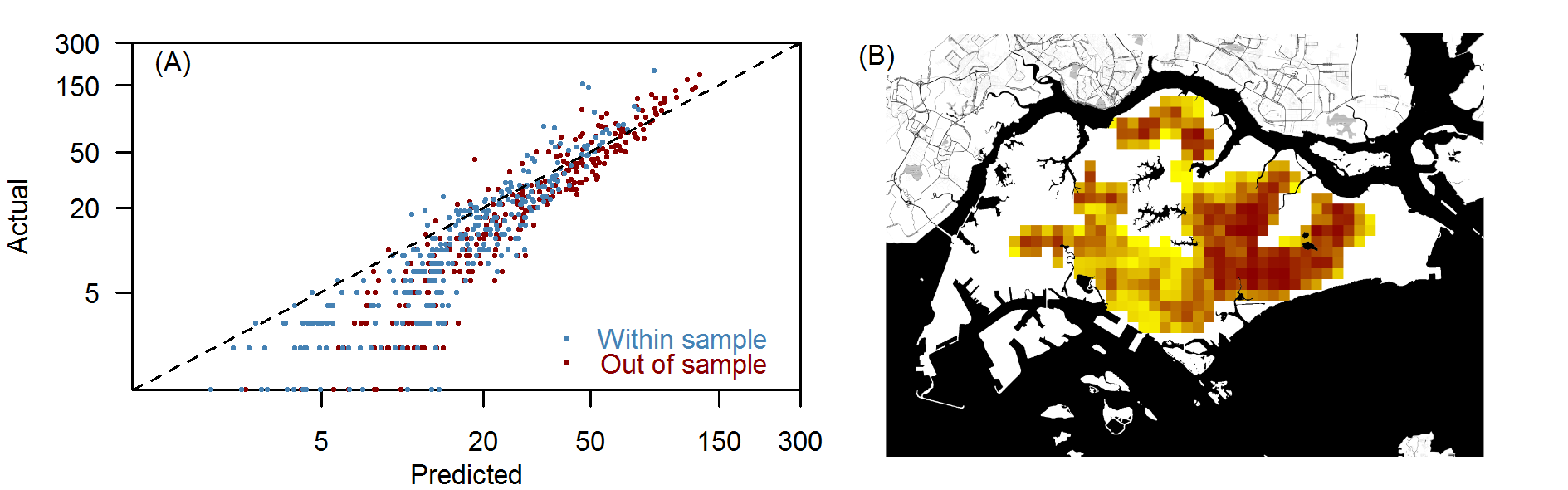

Supplement: Supplementary file 29 — Figure S13. Comparisons of forecast and actual scenario for the 4-week ahead forecast model. a Actual and predicted yearly total number of cases for all neighbourhoods for both within-sample prediction (blue dots) and out-of-sample prediction (dark red dots). b Average risk over all prediction points (both within-sample and out-of-sample) for the 1-week ahead forecast. (PNG 189 kb) [file 12916_2018_1108_MOESM29_ESM.png]

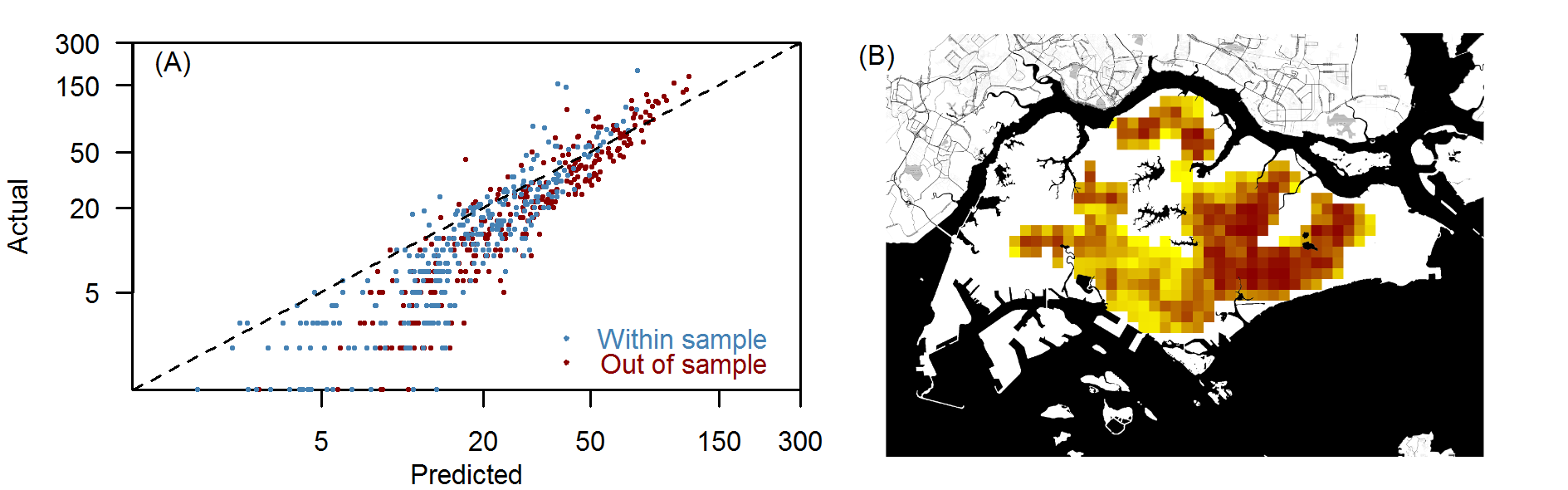

Supplement: Supplementary file 30 — Figure S14. Comparisons of forecast and actual scenario for the 5-week ahead forecast model. a Actual and predicted yearly total number of cases for all neighbourhoods for both within-sample prediction (blue dots) and out-of-sample prediction (dark red dots). b Average risk over all prediction points (both within-sample and out-of-sample) for the 1-week ahead forecast. (PNG 190 kb) [file 12916_2018_1108_MOESM30_ESM.png]

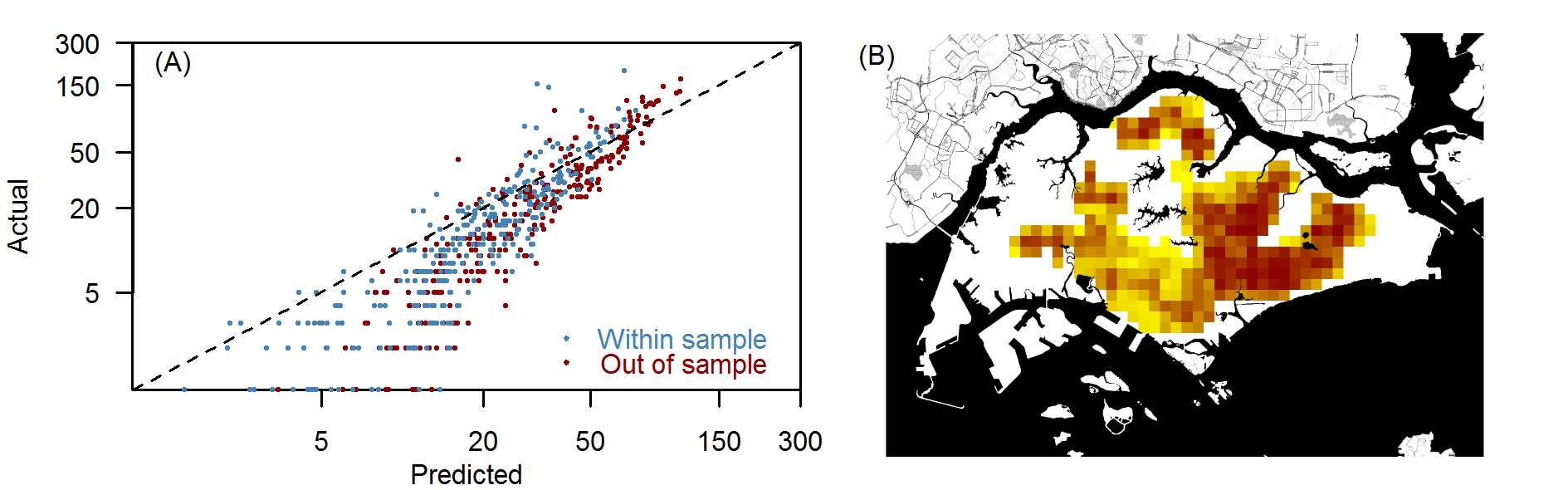

Supplement: Supplementary file 31 — Figure S15. Comparisons of forecast and actual scenario for the 6-week ahead forecast model. a Actual and predicted yearly total number of cases for all neighbourhoods for both within-sample prediction (blue dots) and out-of-sample prediction (dark red dots). b Average risk over all prediction points (both within-sample and out-of-sample) for the 1-week ahead forecast. (PNG 190 kb) [file 12916_2018_1108_MOESM31_ESM.png]

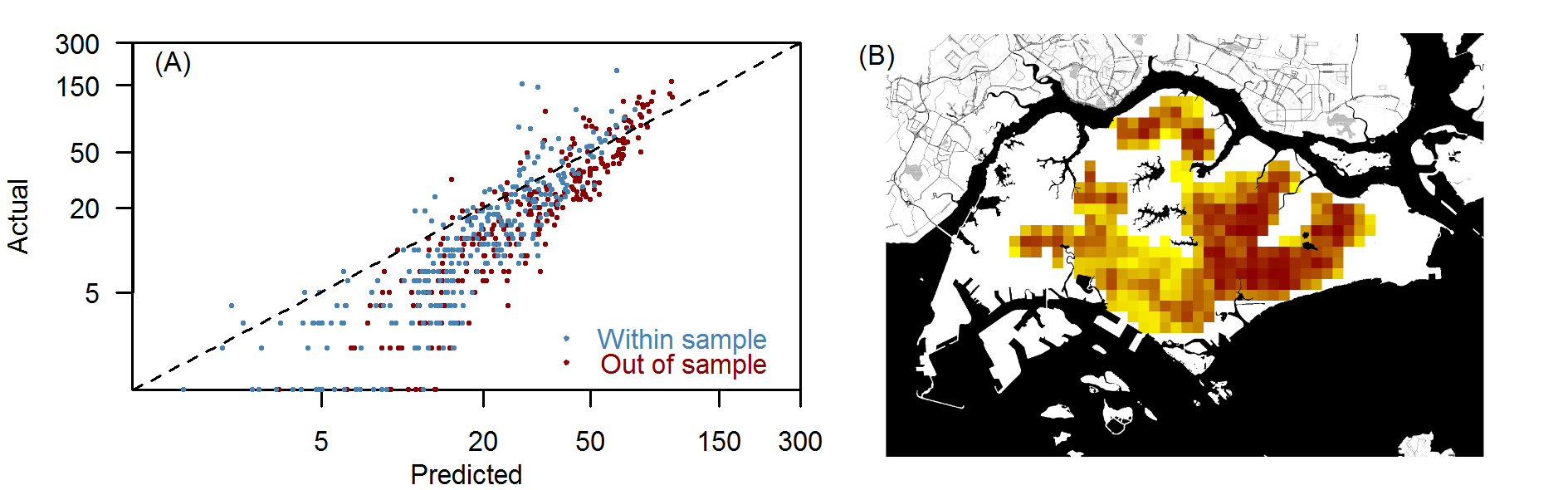

Supplement: Supplementary file 32 — Figure S16. Comparisons of forecast and actual scenario for the 7-week ahead forecast model. a Actual and predicted yearly total number of cases for all neighbourhoods for both within-sample prediction (blue dots) and out-of-sample prediction (dark red dots). b Average risk over all prediction points (both within-sample and out-of-sample) for the 1-week ahead forecast. (PNG 190 kb) [file 12916_2018_1108_MOESM32_ESM.png]

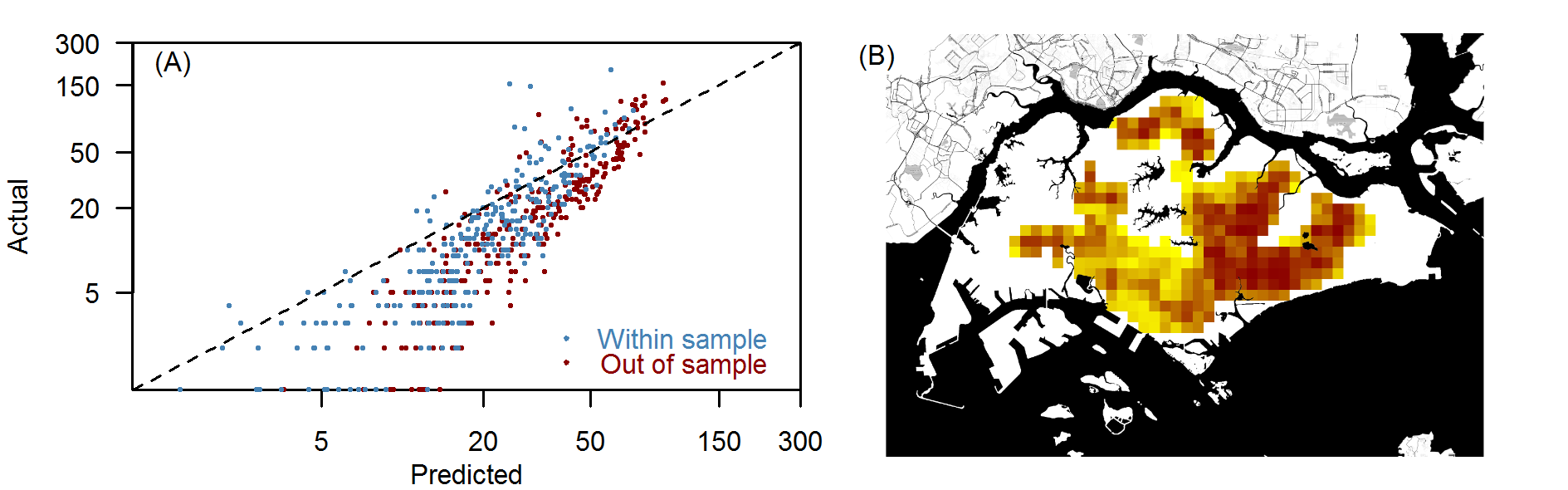

Supplement: Supplementary file 33 — Figure S17. Comparisons of forecast and actual scenario for the 8-week ahead forecast model. a Actual and predicted yearly total number of cases for all neighbourhoods for both within-sample prediction (blue dots) and out-of-sample prediction (dark red dots). b Average risk over all prediction points (both within-sample and out-of-sample) for the 1-week ahead forecast. (PNG 190 kb) [file 12916_2018_1108_MOESM33_ESM.png]

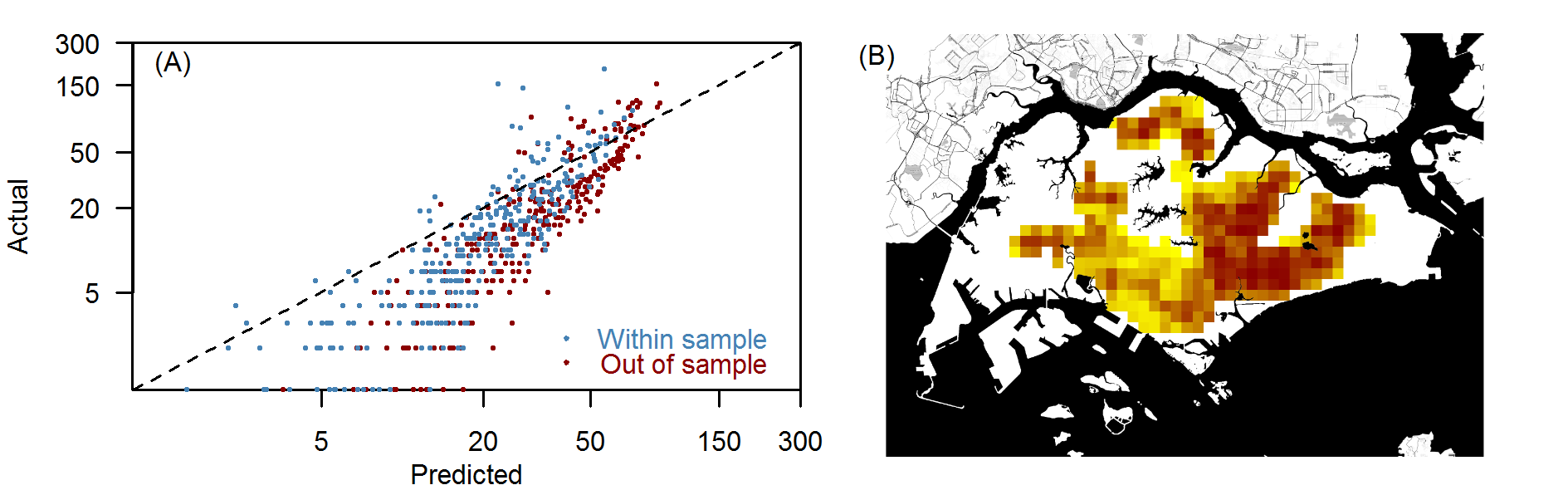

Supplement: Supplementary file 34 — Figure S18. Comparisons of forecast and actual scenario for the 9-week ahead forecast model. a Actual and predicted yearly total number of cases for all neighbourhoods for both within-sample prediction (blue dots) and out-of-sample prediction (dark red dots). b Average risk over all prediction points (both within-sample and out-of-sample) for the 1-week ahead forecast. (PNG 190 kb) [file 12916_2018_1108_MOESM34_ESM.png]

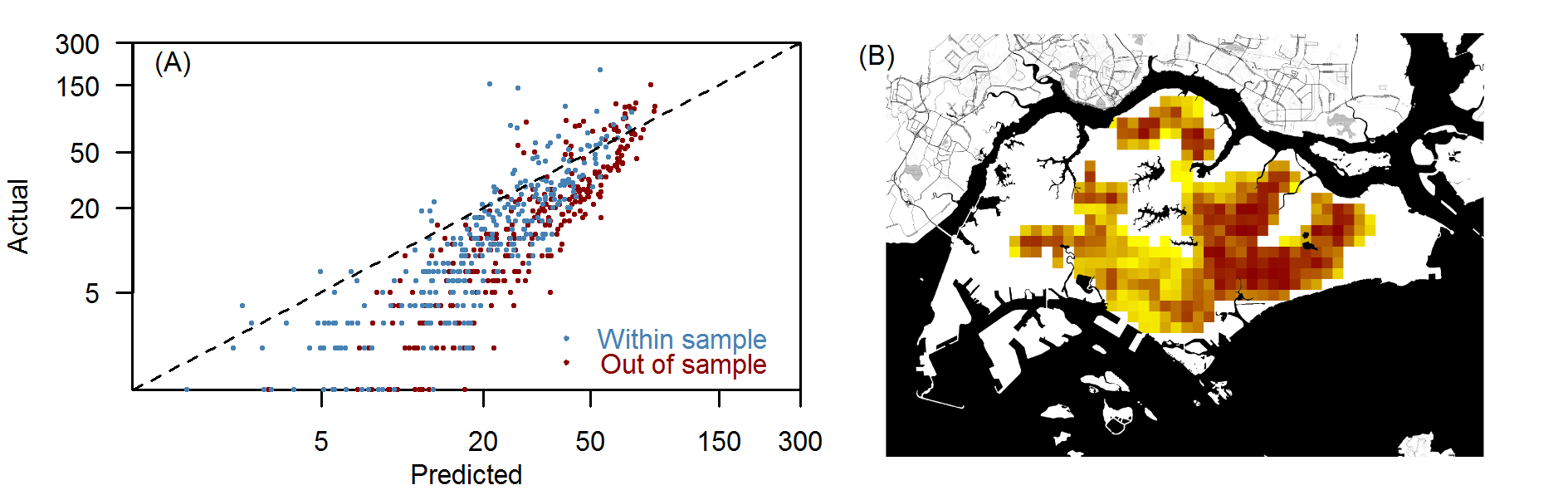

Supplement: Supplementary file 35 — Figure S19. Comparisons of forecast and actual scenario for the 10-week ahead forecast model. a Actual and predicted yearly total number of cases for all neighbourhoods for both within-sample prediction (blue dots) and out-of-sample prediction (dark red dots). b Average risk over all prediction points (both within-sample and out-of-sample) for the 1-week ahead forecast. (PNG 190 kb) [file 12916_2018_1108_MOESM35_ESM.png]

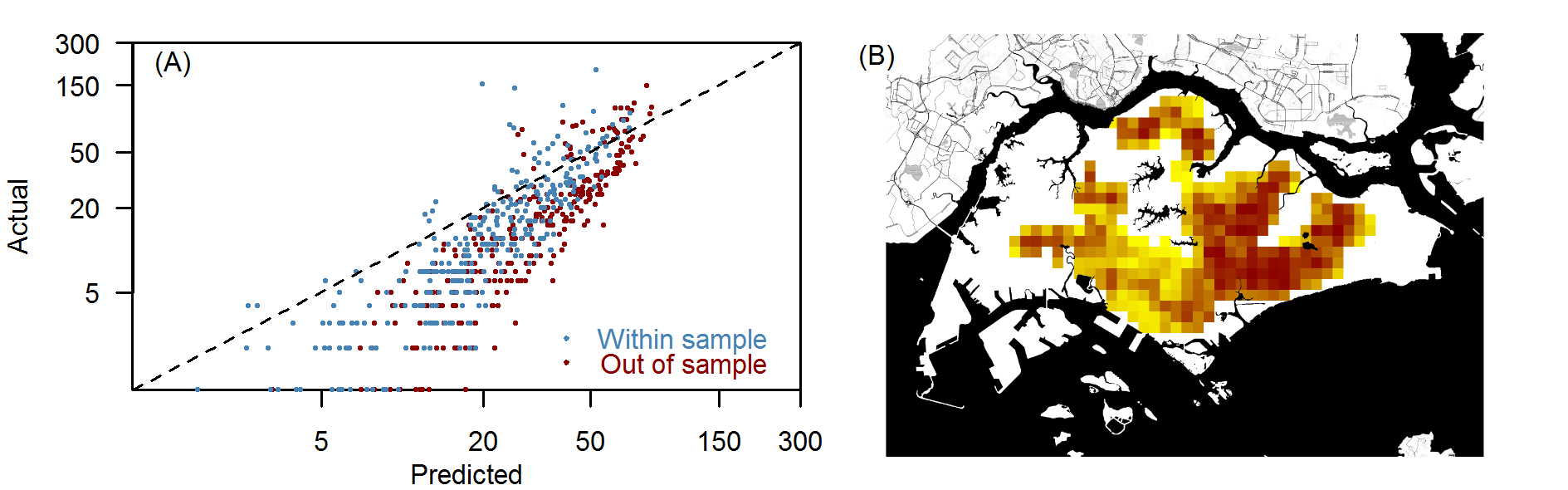

Supplement: Supplementary file 36 — Figure S20. Comparisons of forecast and actual scenario for the 11-week ahead forecast model. a Actual and predicted yearly total number of cases for all neighbourhoods for both within-sample prediction (blue dots) and out-of-sample prediction (dark red dots). b Average risk over all prediction points (both within-sample and out-of-sample) for the 1-week ahead forecast. (PNG 190 kb) [file 12916_2018_1108_MOESM36_ESM.png]

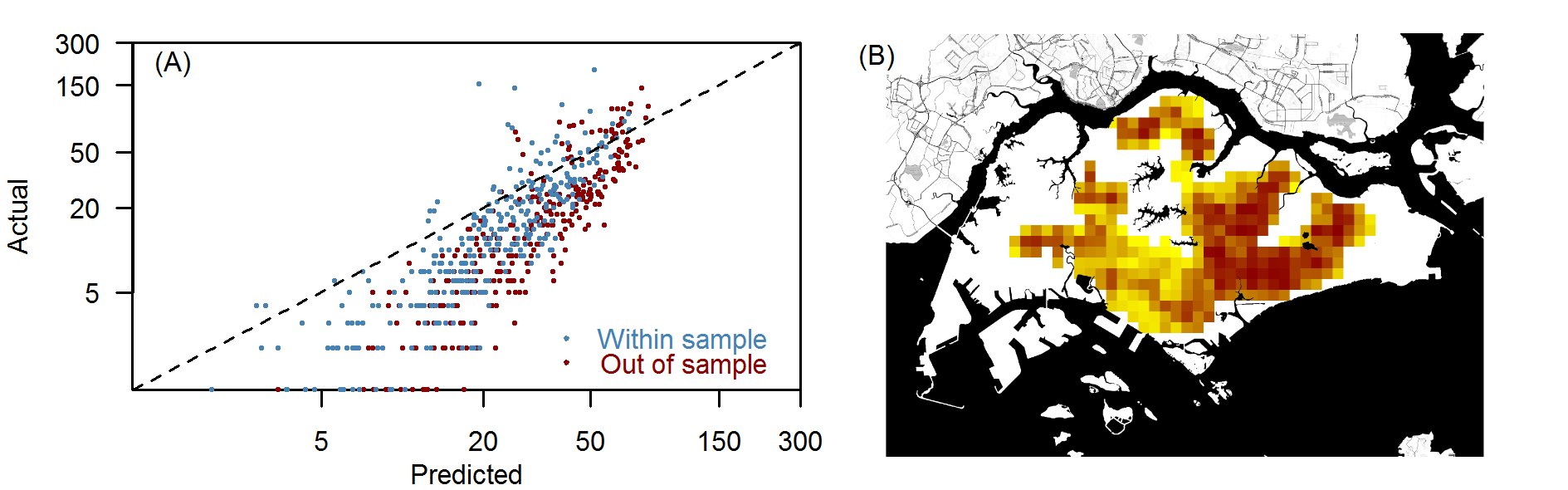

Supplement: Supplementary file 37 — Figure S21. Comparisons of forecast and actual scenario for the 12-week ahead forecast model. a Actual and predicted yearly total number of cases for all neighbourhoods for both within-sample prediction (blue dots) and out-of-sample prediction (dark red dots). b Average risk over all prediction points (both within-sample and out-of-sample) for the 1-week ahead forecast. (PNG 190 kb) [file 12916_2018_1108_MOESM37_ESM.png]
