# Supplementary material for: Neighbourhood level real-time forecasting of dengue cases in tropical urban Singapore
Source: BMC Med. 2018 Aug 6;16:129. doi: 10.1186/s12916-018-1108-5 (PMC6091171; doi:10.1186/s12916-018-1108-5)
Supplement: Supplementary file 15 — Table S1. Groups of variables and their respective types that are included in the LASSO model. (DOCX 11 kb) [file 12916_2018_1108_MOESM3_ESM.docx]

| Variable | Type of Variable |
| --- | --- |
| Past cases | Spatiotemporal |
| Past cases squared | Spatiotemporal |
| Past cases cubic | Spatiotemporal |
| Past cases square root | Spatiotemporal |
| Neighbouring cases | Spatiotemporal |
| Average, minimum, and maximum temperature | Spatiotemporal |
| Average relative humidity | Spatiotemporal |
| Movement derived from telco data | Spatiotemporal |
| Building age | Spatiotemporal |
| National cases | Temporal |
| Vegetation | Spatial |
| Connectivity | Spatial |

Supplementary Table 1. Groups of variables and their respective types that are included in the LASSO model.
