# Supplementary material for: Neighbourhood level real-time forecasting of dengue cases in tropical urban Singapore
Source: BMC Med. 2018 Aug 6;16:129. doi: 10.1186/s12916-018-1108-5 (PMC6091171; doi:10.1186/s12916-018-1108-5)
Supplement: Supplementary file 25 — Table S2. Average Matthew’s correlation coefficient for all 12 forecast windows at 14 different cut-offs. Cut-off are set at different levels so that different percentages of the cells are classified as higher risk areas. (DOCX 12 kb) [file 12916_2018_1108_MOESM25_ESM.docx]

|  | | Forecast Window (in weeks) | | | | | | | | | | | |
| --- | --- | --- | --- | --- | --- | --- | --- | --- | --- | --- | --- | --- | --- |
|  |  | 1 | 2 | 3 | 4 | 5 | 6 | 7 | 8 | 9 | 10 | 11 | 12 |
| Cutoff | 1% | 0.38 | 0.23 | 0.20 | 0.13 | 0.07 | 0.08 | 0.08 | 0.04 | 0.04 | 0.02 | 0.02 | -0.01 |
|  | 3% | 0.41 | 0.33 | 0.26 | 0.22 | 0.17 | 0.15 | 0.14 | 0.12 | 0.09 | 0.10 | 0.08 | 0.05 |
|  | 5% | 0.45 | 0.39 | 0.31 | 0.26 | 0.22 | 0.19 | 0.18 | 0.16 | 0.15 | 0.12 | 0.12 | 0.10 |
|  | 10% | 0.46 | 0.41 | 0.37 | 0.33 | 0.30 | 0.28 | 0.25 | 0.23 | 0.21 | 0.20 | 0.19 | 0.18 |
|  | 15% | 0.45 | 0.42 | 0.38 | 0.35 | 0.32 | 0.31 | 0.28 | 0.28 | 0.27 | 0.26 | 0.25 | 0.24 |
|  | 20% | 0.44 | 0.42 | 0.39 | 0.38 | 0.35 | 0.34 | 0.32 | 0.32 | 0.30 | 0.30 | 0.29 | 0.28 |
|  | 25% | 0.44 | 0.42 | 0.40 | 0.37 | 0.36 | 0.34 | 0.33 | 0.33 | 0.31 | 0.32 | 0.30 | 0.30 |
|  | 30% | 0.44 | 0.43 | 0.42 | 0.40 | 0.37 | 0.36 | 0.35 | 0.34 | 0.33 | 0.32 | 0.31 | 0.32 |
|  | 40% | 0.41 | 0.40 | 0.38 | 0.35 | 0.34 | 0.33 | 0.32 | 0.31 | 0.30 | 0.28 | 0.27 | 0.26 |
|  | 50% | 0.35 | 0.33 | 0.31 | 0.30 | 0.28 | 0.27 | 0.25 | 0.24 | 0.23 | 0.23 | 0.22 | 0.21 |
|  | 60% | 0.29 | 0.28 | 0.27 | 0.26 | 0.25 | 0.25 | 0.23 | 0.23 | 0.22 | 0.21 | 0.21 | 0.20 |
|  | 70% | 0.24 | 0.23 | 0.22 | 0.22 | 0.22 | 0.21 | 0.21 | 0.21 | 0.20 | 0.20 | 0.20 | 0.19 |
|  | 80% | 0.14 | 0.14 | 0.14 | 0.13 | 0.13 | 0.12 | 0.12 | 0.12 | 0.12 | 0.12 | 0.13 | 0.12 |
|  | 90% | 0.06 | 0.06 | 0.05 | 0.04 | 0.04 | 0.04 | 0.05 | 0.06 | 0.06 | 0.06 | 0.06 | 0.05 |

Supplementary Table 2. Average Matthew’s correlation coefficient for all 12 forecast windows at 14 different cutoffs. Cutoff are set at different levels so that different percentages of the cells are classified as higher risk areas.
