# Supplementary material for: Neighbourhood level real-time forecasting of dengue cases in tropical urban Singapore
Source: BMC Med. 2018 Aug 6;16:129. doi: 10.1186/s12916-018-1108-5 (PMC6091171; doi:10.1186/s12916-018-1108-5)
Supplement: Supplementary file 26 — Table S3. Summary of parameters included in LASSO forecast model for the 1-week ahead forecast. Table S4. Summary of parameters included in LASSO forecast model for the 2-week ahead forecast. Table S5. Summary of parameters included in LASSO forecast model for the 3-week ahead forecast. Table S6. Summary of parameters included in LASSO forecast model for the 4-week ahead forecast. Table S7. Summary of parameters included in LASSO forecast model for the 5-week ahead forecast. Table S8. Summary of parameters included in LASSO forecast model for the 6-week ahead forecast. Table S9. Summary of parameters included in LASSO forecast model for the 7-week ahead forecast. Table S10. Summary of parameters included in LASSO forecast model for the 8-week ahead forecast. Table S11. Summary of parameters included in LASSO forecast model for the 9-week ahead forecast. Table S12. Summary of parameters included in LASSO forecast model for the 10-week ahead forecast. Table S13. Summary of parameters included in LASSO forecast model for the 11-week ahead forecast. Table S14. Summary of parameters included in LASSO forecast model for the 12-week ahead forecast. (DOCX 37 kb) [file 12916_2018_1108_MOESM26_ESM.docx]

| Variable | Inclusion probability (over bootstrap samples) | Estimated beta | Bootstrap confidence interval |
| --- | --- | --- | --- |
| Cases lag 1 | 0.86 | 0.32 | (0, 0.51) |
| Cases lag 2 | 0.98 | 0.31 | (0.01, 0.38) |
| Cases lag 3 | 1 | 0.2 | (0.14, 0.3) |
| Cases lag 4 | 1 | 0.1 | (0.06, 0.16) |
| Cases lag 5 | 0.99 | 0.04 | (0.01, 0.07) |
| Cases lag 6 | 1 | 0.05 | (0.03, 0.11) |
| Cases lag 7 | 0.86 | 0.02 | (-0.01, 0.06) |
| Cases lag 8 | 0.7 | 0.01 | (-0.02, 0.04) |
| Cases square lag 1 | 0.99 | 0.05 | (-0.04, 0.18) |
| Cases square lag 2 | 1 | 0.07 | (0.03, 0.18) |
| Cases square lag 3 | 0.92 | 0.03 | (0, 0.06) |
| Cases square lag 4 | 0.59 | 0 | (0, 0.02) |
| Cases square lag 5 | 0.92 | -0.01 | (-0.03, 0) |
| Cases square lag 6 | 0.63 | 0 | (-0.02, 0.02) |
| Cases square lag 7 | 0.91 | -0.01 | (-0.03, 0) |
| Cases square lag 8 | 0.69 | 0 | (-0.01, 0.01) |
| Cases cubic lag 1 | 0.9 | 0.02 | (0, 0.04) |
| Cases cubic lag 2 | 1 | -0.02 | (-0.03, -0.01) |
| Cases cubic lag 3 | 1 | -0.02 | (-0.03, -0.01) |
| Cases cubic lag 4 | 1 | -0.01 | (-0.01, 0) |
| Cases cubic lag 5 | 0.76 | 0 | (-0.01, 0.01) |
| Cases cubic lag 6 | 0.98 | -0.01 | (-0.01, 0) |
| Cases cubic lag 7 | 0.78 | 0 | (-0.01, 0.01) |
| Cases cubic lag 8 | 0.78 | 0 | (0, 0) |
| Cases square root lag 1 | 1 | -0.24 | (-0.36, -0.02) |
| Cases square root lag 2 | 1 | -0.24 | (-0.29. -0.05) |
| Cases square root lag 3 | 1 | -0.12 | (-0.19, -0.08) |
| Cases square root lag 4 | 0.98 | -0.04 | (-0.09, 0) |
| Cases square root lag 5 | 0.67 | 0 | (-0.02, 0.04) |
| Cases square root lag 6 | 0.8 | -0.01 | (-0.06, 0.01) |
| Cases square root lag 7 | 0.8 | 0.02 | (0, 0.05) |
| Cases square root lag 8 | 0.9 | 0.02 | (0, 0.05) |
| 1-km neighbour lag 1 | 1 | 0.03 | (0.02, 0.03) |
| 1-km neighbour lag 2 | 1 | 0.02 | (0.01, 0.02) |
| 1-km neighbour lag 3 | 1 | 0.01 | (0, 0.01) |
| 1-km neighbour lag 4 | 0.91 | 0 | (0, 0.01) |
| 1-km neighbour lag 5 | 0.93 | 0 | (0, 0.01) |
| 1-km neighbour lag 6 | 0.91 | 0 | (-0.01, 0) |
| 1-km neighbour lag 7 | 0.94 | 0 | (-0.01, 0) |
| 1-km neighbour lag 8 | 0.9 | 0 | (0, 0) |
| 2-km neighbour lag 1 | 1 | 0.01 | (0.01, 0.02) |
| 2-km neighbour lag 2 | 1 | 0.01 | (0, 0.01) |
| 2-km neighbour lag 3 | 0.88 | 0 | (0, 0) |
| 2-km neighbour lag 4 | 0.9 | 0 | (-0.01, 0) |
| 2-km neighbour lag 5 | 0.99 | 0 | (-0.01, 0) |
| 2-km neighbour lag 6 | 0.87 | 0 | (0, 0) |
| 2-km neighbour lag 7 | 0.99 | 0 | (-0.01, 0) |
| 2-km neighbour lag 8 | 0.87 | 0 | (0, 0) |
| National level cases | 1 | 0.02 | (0.01, 0.02) |
| Vegetation | 1 | -0.21 | (-0.24, -0.18) |
| Building age | 0.99 | 0 | (0, 0) |
| Connectivity | 1 | 0 | (0, 0) |
| Mean temperature lag 1 | 1 | 0.01 | (0, 0.02) |
| Mean temperature lag 2 | 0.79 | 0 | (0, 0.01) |
| Mean temperature lag 3 | 0.99 | -0.01 | (-0.02, 0) |
| Mean temperature lag 4 | 0.78 | 0 | (-0.01, 0.01) |
| Mean temperature lag 5 | 0.74 | 0 | (-0.01, 0.01) |
| Max temperature lag 1 | 0.84 | 0 | (0, 0) |
| Max temperature lag 2 | 0.85 | 0 | (0, 0) |
| Max temperature lag 3 | 0.95 | 0 | (0, 0.01) |
| Max temperature lag 4 | 0.93 | 0 | (0, 0.01) |
| Max temperature lag 5 | 1 | 0 | (0, 0.01) |
| Min temperature lag 1 | 0.89 | 0 | (-0.01, 0) |
| Min temperature lag 2 | 0.83 | 0 | (0, 0.01) |
| Min temperature lag 3 | 0.86 | 0 | (-0.01, 0) |
| Min temperature lag 4 | 0.99 | 0.01 | (0, 0.01) |
| Min temperature lag 5 | 0.9 | 0 | (-0.01, 0) |
| Mean humidity lag 1 | 0.92 | 0 | (0, 0) |
| Mean humidity lag 2 | 0.9 | 0 | (0, 0) |
| Mean humidity lag 3 | 0.9 | 0 | (0, 0) |
| Mean humidity lag 4 | 1 | 0 | (0, 0) |
| Mean humidity lag 5 | 1 | 0 | (0, 0) |
| Telco | 1 | 0.09 | (0.07, 0.11) |

Supplementary Table 3. Summary of parameters included in LASSO forecast model for 1-week ahead forecast. Inclusion probabilities, average estimated parameter value, 95% confidence intervals are calculated based on 1000 bootstrap samples.

| Variable | Inclusion probability (over bootstrap samples) | Estimated beta | Bootstrap confidence interval |
| --- | --- | --- | --- |
| Cases lag 1 | 0.81 | 0.29 | (-0.02, 0.51) |
| Cases lag 2 | 0.91 | 0.25 | (0, 0.34) |
| Cases lag 3 | 1 | 0.18 | (0.08, 0.28) |
| Cases lag 4 | 1 | 0.09 | (0.05, 0.19) |
| Cases lag 5 | 1 | 0.08 | (0.04, 0.19) |
| Cases lag 6 | 0.98 | 0.04 | (0, 0.09) |
| Cases lag 7 | 0.72 | 0.01 | (-0.03, 0.05) |
| Cases lag 8 | 0.97 | 0.03 | (0, 0.07) |
| Cases square lag 1 | 1 | 0.07 | (-0.02, 0.21) |
| Cases square lag 2 | 1 | 0.08 | (0.04, 0.19) |
| Cases square lag 3 | 0.88 | 0.03 | (0, 0.07) |
| Cases square lag 4 | 0.8 | -0.01 | (-0.05, 0) |
| Cases square lag 5 | 0.84 | -0.01 | (-0.05, 0.02) |
| Cases square lag 6 | 0.95 | -0.01 | (-0.04, 0.01) |
| Cases square lag 7 | 0.88 | -0.01 | (-0.03, 0) |
| Cases square lag 8 | 0.7 | 0 | (-0.02, 0.02) |
| Cases cubic lag 1 | 0.95 | 0.01 | (-0.01, 0.03) |
| Cases cubic lag 2 | 1 | -0.02 | (-0.04, -0.01) |
| Cases cubic lag 3 | 1 | -0.02 | (-0.03, -0.01) |
| Cases cubic lag 4 | 0.92 | 0 | (-0.01, 0) |
| Cases cubic lag 5 | 0.94 | -0.01 | (-0.01, 0) |
| Cases cubic lag 6 | 0.83 | 0 | (-0.01, 0.01) |
| Cases cubic lag 7 | 0.8 | 0 | (0, 0.01) |
| Cases cubic lag 8 | 0.93 | 0 | (-0.01, 0) |
| Cases square root lag 1 | 1 | -0.23 | (-0.38, -0.03) |
| Cases square root lag 2 | 1 | -0.18 | (-0.25, -0.02) |
| Cases square root lag 3 | 0.99 | -0.11 | (-0.18, -0.04) |
| Cases square root lag 4 | 0.91 | -0.03 | (-0.11, 0) |
| Cases square root lag 5 | 0.88 | -0.03 | (-0.1, 0) |
| Cases square root lag 6 | 0.71 | 0.01 | (-0.03, 0.04) |
| Cases square root lag 7 | 0.87 | 0.03 | (0, 0.06) |
| Cases square root lag 8 | 0.71 | 0 | (-0.03, 0.03) |
| 1-km neighbour lag 1 | 1 | 0.03 | (0.03, 0.04) |
| 1-km neighbour lag 2 | 1 | 0.02 | (0.02, 0.03) |
| 1-km neighbour lag 3 | 1 | 0.01 | (0, 0.01) |
| 1-km neighbour lag 4 | 0.98 | 0 | (0, 0.01) |
| 1-km neighbour lag 5 | 0.93 | 0 | (-0.01, 0) |
| 1-km neighbour lag 6 | 0.96 | 0 | (-0.01, 0) |
| 1-km neighbour lag 7 | 0.92 | 0 | (-0.01, 0) |
| 1-km neighbour lag 8 | 0.91 | 0 | (0, 0.01) |
| 2-km neighbour lag 1 | 1 | 0.01 | (0.01, 0.02) |
| 2-km neighbour lag 2 | 1 | 0.01 | (0, 0.01) |
| 2-km neighbour lag 3 | 0.89 | 0 | (0, 0) |
| 2-km neighbour lag 4 | 0.97 | 0 | (-0.01, 0) |
| 2-km neighbour lag 5 | 0.88 | 0 | (-0.01, 0) |
| 2-km neighbour lag 6 | 0.96 | 0 | (-0.01, 0) |
| 2-km neighbour lag 7 | 0.91 | 0 | (-0.01, 0) |
| 2-km neighbour lag 8 | 0.91 | 0 | (0, 0) |
| National level cases | 1 | 0.02 | (0.01, 0.02) |
| Vegetation | 1 | -0.25 | (-0.28, -0.22) |
| Building age | 1 | 0 | (0, 0) |
| Connectivity | 1 | 0 | (0, 0) |
| Mean temperature lag 1 | 1 | 0.02 | (0.01, 0.03) |
| Mean temperature lag 2 | 0.78 | 0 | (-0.01, 0) |
| Mean temperature lag 3 | 0.95 | -0.01 | (-0.02, 0) |
| Mean temperature lag 4 | 0.84 | 0 | (0, 0.02) |
| Mean temperature lag 5 | 1 | -0.02 | (-0.03, -0.01) |
| Max temperature lag 1 | 0.92 | 0 | (-0.01, 0) |
| Max temperature lag 2 | 0.86 | 0 | (0, 0) |
| Max temperature lag 3 | 0.99 | 0.01 | (0, 0.01) |
| Max temperature lag 4 | 0.89 | 0 | (0, 0.01) |
| Max temperature lag 5 | 1 | 0.01 | (0.01, 0.01) |
| Min temperature lag 1 | 0.9 | 0 | (-0.01, 0) |
| Min temperature lag 2 | 0.9 | 0 | (-0.01, 0) |
| Min temperature lag 3 | 0.98 | 0.01 | (0, 0.01) |
| Min temperature lag 4 | 0.85 | 0 | (-0.01, 0.01) |
| Min temperature lag 5 | 0.86 | 0 | (0, 0.01) |
| Mean humidity lag 1 | 0.88 | 0 | (0, 0) |
| Mean humidity lag 2 | 0.85 | 0 | (0, 0) |
| Mean humidity lag 3 | 0.97 | 0 | (0, 0) |
| Mean humidity lag 4 | 1 | 0 | (0, 0) |
| Mean humidity lag 5 | 0.92 | 0 | (0, 0) |
| Telco | 1 | 0.1 | (0.08, 0.12) |

Supplementary Table 4. Summary of parameters included in LASSO forecast model for 2-week ahead forecast. Inclusion probabilities, average estimated parameter value, 95% confidence intervals are calculated based on 1000 bootstrap samples.

| Variable | Inclusion probability (over bootstrap samples) | Estimated beta | Bootstrap confidence interval |
| --- | --- | --- | --- |
| Cases lag 1 | 0.94 | 0.28 | (-0.05, 0.48) |
| Cases lag 2 | 0.93 | 0.24 | (0, 0.33) |
| Cases lag 3 | 1 | 0.19 | (0.13, 0.33) |
| Cases lag 4 | 1 | 0.16 | (0.08, 0.31) |
| Cases lag 5 | 0.99 | 0.06 | (0, 0.17) |
| Cases lag 6 | 0.81 | 0.02 | (-0.02, 0.06) |
| Cases lag 7 | 0.92 | 0.03 | (0, 0.08) |
| Cases lag 8 | 0.92 | 0.03 | (0, 0.09) |
| Cases square lag 1 | 1 | 0.07 | (-0.01, 0.22) |
| Cases square lag 2 | 1 | 0.07 | (0.02, 0.18) |
| Cases square lag 3 | 0.83 | 0 | (-0.06, 0.03) |
| Cases square lag 4 | 0.9 | -0.01 | (-0.07, 0.03) |
| Cases square lag 5 | 0.98 | -0.02 | (-0.06, 0) |
| Cases square lag 6 | 0.9 | -0.01 | (-0.04, 0.01) |
| Cases square lag 7 | 0.85 | 0 | (-0.03, 0.02) |
| Cases square lag 8 | 0.88 | -0.01 | (-0.04, 0.01) |
| Cases cubic lag 1 | 1 | 0.01 | (-0.02, 0.03) |
| Cases cubic lag 2 | 1 | -0.03 | (-0.05, -0.01) |
| Cases cubic lag 3 | 0.99 | -0.01 | (-0.02, 0) |
| Cases cubic lag 4 | 0.98 | -0.01 | (-0.02, 0) |
| Cases cubic lag 5 | 0.83 | 0 | (-0.01, 0.01) |
| Cases cubic lag 6 | 0.84 | 0 | (-0.01, 0.01) |
| Cases cubic lag 7 | 0.85 | 0 | (-0.01, 0.01) |
| Cases cubic lag 8 | 0.88 | 0 | (-0.01, 0) |
| Cases square root lag 1 | 0.98 | -0.21 | (-0.35, 0) |
| Cases square root lag 2 | 1 | -0.18 | (-0.25, -0.03) |
| Cases square root lag 3 | 1 | -0.12 | (-0.21, -0.06) |
| Cases square root lag 4 | 1 | -0.09 | (-0.19, -0.02) |
| Cases square root lag 5 | 0.84 | 0 | (-0.08, 0.04) |
| Cases square root lag 6 | 0.81 | 0.02 | (-0.01, 0.06) |
| Cases square root lag 7 | 0.77 | 0.01 | (-0.03, 0.04) |
| Cases square root lag 8 | 0.82 | 0.01 | (-0.03, 0.04) |
| 1-km neighbour lag 1 | 1 | 0.04 | (0.03, 0.04) |
| 1-km neighbour lag 2 | 1 | 0.02 | (0.01, 0.03) |
| 1-km neighbour lag 3 | 1 | 0.01 | (0.01, 0.02) |
| 1-km neighbour lag 4 | 0.93 | 0 | (0, 0.01) |
| 1-km neighbour lag 5 | 0.96 | 0 | (-0.01, 0) |
| 1-km neighbour lag 6 | 0.94 | 0 | (-0.01, 0) |
| 1-km neighbour lag 7 | 0.93 | 0 | (-0.01, 0) |
| 1-km neighbour lag 8 | 0.93 | 0 | (0, 0.01) |
| 2-km neighbour lag 1 | 1 | 0.02 | (0.01, 0.02) |
| 2-km neighbour lag 2 | 1 | 0.01 | (0, 0.01) |
| 2-km neighbour lag 3 | 0.92 | 0 | (-0.01, 0) |
| 2-km neighbour lag 4 | 0.91 | 0 | (0, 0) |
| 2-km neighbour lag 5 | 0.98 | 0 | (-0.01, 0) |
| 2-km neighbour lag 6 | 0.9 | 0 | (0, 0) |
| 2-km neighbour lag 7 | 0.96 | 0 | (-0.01, 0) |
| 2-km neighbour lag 8 | 0.93 | 0 | (-0.01, 0) |
| National level cases | 1 | 0.02 | (0.02, 0.03) |
| Vegetation | 1 | -0.3 | (-0.33, -0.26) |
| Building age | 1 | 0 | (0, 0) |
| Connectivity | 1 | 0 | (0, 0) |
| Mean temperature lag 1 | 1 | 0.02 | (0.01, 0.03) |
| Mean temperature lag 2 | 0.76 | 0 | (-0.01, 0.01) |
| Mean temperature lag 3 | 0.75 | 0 | (-0.01, 0.01) |
| Mean temperature lag 4 | 0.91 | -0.01 | (-0.02, 0) |
| Mean temperature lag 5 | 1 | -0.02 | (-0.03, -0.01) |
| Max temperature lag 1 | 0.91 | 0 | (-0.01, 0) |
| Max temperature lag 2 | 0.97 | 0 | (0, 0.01) |
| Max temperature lag 3 | 0.99 | 0.01 | (0, 0.01) |
| Max temperature lag 4 | 1 | 0.01 | (0, 0.01) |
| Max temperature lag 5 | 0.97 | 0 | (0, 0.01) |
| Min temperature lag 1 | 0.99 | -0.01 | (-0.01, 0) |
| Min temperature lag 2 | 0.97 | 0 | (0, 0.01) |
| Min temperature lag 3 | 0.87 | 0 | (-0.01, 0.01) |
| Min temperature lag 4 | 0.92 | 0 | (0, 0.01) |
| Min temperature lag 5 | 0.97 | 0.01 | (0, 0.01) |
| Mean humidity lag 1 | 0.89 | 0 | (0, 0) |
| Mean humidity lag 2 | 1 | 0 | (0, 0) |
| Mean humidity lag 3 | 1 | 0 | (0, 0) |
| Mean humidity lag 4 | 1 | 0 | (0, 0) |
| Mean humidity lag 5 | 1 | 0 | (0, 0) |
| Telco | 1 | 0.09 | (0.07, 0.11) |

Supplementary Table 5. Summary of parameters included in LASSO forecast model for 3-week ahead forecast. Inclusion probabilities, average estimated parameter value, 95% confidence intervals are calculated based on 1000 bootstrap samples.

| Variable | Inclusion probability (over bootstrap samples) | Estimated beta | Bootstrap confidence interval |
| --- | --- | --- | --- |
| Cases lag 1 | 0.93 | 0.25 | (0, 0.44) |
| Cases lag 2 | 1 | 0.3 | (0.23, 0.41) |
| Cases lag 3 | 1 | 0.23 | (0.16, 0.38) |
| Cases lag 4 | 1 | 0.11 | (0.05, 0.25) |
| Cases lag 5 | 0.93 | 0.03 | (-0.01, 0.1) |
| Cases lag 6 | 0.98 | 0.04 | (0, 0.12) |
| Cases lag 7 | 0.95 | 0.03 | (-0.01, 0.11) |
| Cases lag 8 | 0.83 | 0.02 | (-0.02, 0.08) |
| Cases square lag 1 | 1 | 0.08 | (-0.01, 0.21) |
| Cases square lag 2 | 0.9 | 0.02 | (-0.01, 0.06) |
| Cases square lag 3 | 0.84 | 0.01 | (-0.05, 0.06) |
| Cases square lag 4 | 0.9 | -0.02 | (-0.06, 0.01) |
| Cases square lag 5 | 0.96 | -0.01 | (-0.05, 0) |
| Cases square lag 6 | 0.86 | 0 | (-0.03, 0.02) |
| Cases square lag 7 | 0.96 | -0.02 | (-0.06, 0) |
| Cases square lag 8 | 0.92 | -0.01 | (-0.04, 0.01) |
| Cases cubic lag 1 | 1 | 0 | (-0.02, 0.02) |
| Cases cubic lag 2 | 1 | -0.02 | (-0.03, -0.01) |
| Cases cubic lag 3 | 1 | -0.02 | (-0.03, -0.01) |
| Cases cubic lag 4 | 0.93 | -0.01 | (-0.01, 0) |
| Cases cubic lag 5 | 0.84 | 0 | (0, 0.01) |
| Cases cubic lag 6 | 0.88 | 0 | (-0.01, 0) |
| Cases cubic lag 7 | 0.84 | 0 | (-0.01, 0.01) |
| Cases cubic lag 8 | 0.86 | 0 | (-0.01, 0.01) |
| Cases square root lag 1 | 1 | -0.19 | (-0.33, -0.02) |
| Cases square root lag 2 | 1 | -0.21 | (-0.29, -0.15) |
| Cases square root lag 3 | 1 | -0.15 | (-0.25, -0.09) |
| Cases square root lag 4 | 0.94 | -0.04 | (-0.14, 0) |
| Cases square root lag 5 | 0.82 | 0.01 | (-0.03, 0.06) |
| Cases square root lag 6 | 0.8 | 0 | (-0.06, 0.03) |
| Cases square root lag 7 | 0.84 | 0.01 | (-0.03, 0.05) |
| Cases square root lag 8 | 0.89 | 0.03 | (0, 0.07) |
| 1-km neighbour lag 1 | 1 | 0.04 | (0.03, 0.04) |
| 1-km neighbour lag 2 | 1 | 0.02 | (0.02, 0.03) |
| 1-km neighbour lag 3 | 1 | 0.01 | (0, 0.01) |
| 1-km neighbour lag 4 | 0.92 | 0 | (-0.01, 0.01) |
| 1-km neighbour lag 5 | 0.95 | 0 | (-0.01, 0) |
| 1-km neighbour lag 6 | 0.93 | 0 | (-0.01, 0) |
| 1-km neighbour lag 7 | 0.93 | 0 | (-0.01, 0.01) |
| 1-km neighbour lag 8 | 0.94 | 0 | (-0.01, 0) |
| 2-km neighbour lag 1 | 1 | 0.02 | (0.01, 0.02) |
| 2-km neighbour lag 2 | 1 | 0.01 | (0, 0.01) |
| 2-km neighbour lag 3 | 0.96 | 0 | (0, 0.01) |
| 2-km neighbour lag 4 | 0.96 | 0 | (-0.01, 0) |
| 2-km neighbour lag 5 | 0.92 | 0 | (-0.01, 0) |
| 2-km neighbour lag 6 | 0.91 | 0 | (-0.01, 0) |
| 2-km neighbour lag 7 | 0.96 | 0 | (-0.01, 0) |
| 2-km neighbour lag 8 | 0.92 | 0 | (0, 0) |
| National level cases | 1 | 0.02 | (0.02, 0.03) |
| Vegetation | 1 | -0.33 | (-0.36, -0.3) |
| Building age | 1 | 0 | (0, 0) |
| Connectivity | 1 | 0 | (0, 0) |
| Mean temperature lag 1 | 1 | 0.02 | (0.01, 0.03) |
| Mean temperature lag 2 | 0.98 | 0.01 | (0, 0.02) |
| Mean temperature lag 3 | 0.9 | -0.01 | (-0.02, 0) |
| Mean temperature lag 4 | 0.8 | 0 | (-0.01, 0.01) |
| Mean temperature lag 5 | 1 | -0.03 | (-0.05, -0.02) |
| Max temperature lag 1 | 0.9 | 0 | (0, 0) |
| Max temperature lag 2 | 0.89 | 0 | (0, 0.01) |
| Max temperature lag 3 | 1 | 0.01 | (0, 0.01) |
| Max temperature lag 4 | 0.94 | 0 | (-0.01, 0) |
| Max temperature lag 5 | 1 | 0.02 | (0.01, 0.02) |
| Min temperature lag 1 | 0.9 | 0 | (-0.01, 0) |
| Min temperature lag 2 | 0.91 | 0 | (-0.01, 0) |
| Min temperature lag 3 | 0.9 | 0 | (0, 0.01) |
| Min temperature lag 4 | 0.98 | 0.01 | (0, 0.01) |
| Min temperature lag 5 | 0.98 | 0.01 | (0, 0.01) |
| Mean humidity lag 1 | 1 | 0 | (0, 0) |
| Mean humidity lag 2 | 1 | 0 | (0, 0.01) |
| Mean humidity lag 3 | 1 | 0 | (0, 0) |
| Mean humidity lag 4 | 0.99 | 0 | (0, 0) |
| Mean humidity lag 5 | 1 | 0 | (0, 0) |
| Telco | 1 | 0.09 | (0.07, 0.12) |

Supplementary Table 6. Summary of parameters included in LASSO forecast model for 4-week ahead forecast. Inclusion probabilities, average estimated parameter value, 95% confidence intervals are calculated based on 1000 bootstrap samples.

| Variable | Inclusion probability (over bootstrap samples) | Estimated beta | Bootstrap confidence interval |
| --- | --- | --- | --- |
| Cases lag 1 | 0.97 | 0.36 | (0, 0.44) |
| Cases lag 2 | 1 | 0.29 | (0.24, 0.37) |
| Cases lag 3 | 1 | 0.15 | (0.1, 0.21) |
| Cases lag 4 | 1 | 0.05 | (0.01, 0.1) |
| Cases lag 5 | 1 | 0.05 | (0.01, 0.1) |
| Cases lag 6 | 0.98 | 0.03 | (0, 0.08) |
| Cases lag 7 | 0.8 | 0.01 | (-0.02, 0.06) |
| Cases lag 8 | 0.9 | 0.02 | (-0.01, 0.05) |
| Cases square lag 1 | 0.97 | 0.01 | (-0.03, 0.17) |
| Cases square lag 2 | 0.99 | 0.05 | (0.01, 0.08) |
| Cases square lag 3 | 0.89 | 0.01 | (-0.01, 0.03) |
| Cases square lag 4 | 0.89 | 0 | (-0.02, 0.02) |
| Cases square lag 5 | 0.92 | 0 | (-0.02, 0.02) |
| Cases square lag 6 | 0.96 | -0.01 | (-0.04, 0.01) |
| Cases square lag 7 | 0.96 | -0.01 | (-0.04, 0) |
| Cases square lag 8 | 0.79 | 0 | (-0.02, 0.01) |
| Cases cubic lag 1 | 0.99 | 0.01 | (-0.02, 0.02) |
| Cases cubic lag 2 | 1 | -0.03 | (-0.04, -0.02) |
| Cases cubic lag 3 | 1 | -0.01 | (-0.02, 0) |
| Cases cubic lag 4 | 0.92 | 0 | (-0.01, 0.01) |
| Cases cubic lag 5 | 0.93 | 0 | (-0.01, 0) |
| Cases cubic lag 6 | 0.91 | 0 | (-0.01, 0.01) |
| Cases cubic lag 7 | 0.88 | 0 | (-0.01, 0.01) |
| Cases cubic lag 8 | 0.91 | 0 | (-0.01, 0) |
| Cases square root lag 1 | 1 | -0.26 | (-0.33, -0.03) |
| Cases square root lag 2 | 1 | -0.21 | (-0.27, -0.16) |
| Cases square root lag 3 | 1 | -0.08 | (-0.14, -0.04) |
| Cases square root lag 4 | 0.84 | -0.01 | (-0.05, 0.03) |
| Cases square root lag 5 | 0.84 | -0.01 | (-0.06, 0.03) |
| Cases square root lag 6 | 0.91 | 0.01 | (-0.02, 0.05) |
| Cases square root lag 7 | 0.97 | 0.04 | (0, 0.07) |
| Cases square root lag 8 | 0.93 | 0.02 | (-0.01, 0.05) |
| 1-km neighbour lag 1 | 1 | 0.04 | (0.04, 0.05) |
| 1-km neighbour lag 2 | 1 | 0.02 | (0.01, 0.03) |
| 1-km neighbour lag 3 | 0.99 | 0.01 | (0, 0.01) |
| 1-km neighbour lag 4 | 0.93 | 0 | (0, 0.01) |
| 1-km neighbour lag 5 | 0.94 | 0 | (-0.01, 0) |
| 1-km neighbour lag 6 | 0.93 | 0 | (-0.01, 0) |
| 1-km neighbour lag 7 | 0.98 | 0 | (-0.01, 0) |
| 1-km neighbour lag 8 | 0.99 | 0.01 | (0, 0.01) |
| 2-km neighbour lag 1 | 1 | 0.02 | (0.01, 0.02) |
| 2-km neighbour lag 2 | 1 | 0.01 | (0.01, 0.02) |
| 2-km neighbour lag 3 | 0.9 | 0 | (0, 0.01) |
| 2-km neighbour lag 4 | 0.9 | 0 | (0, 0.01) |
| 2-km neighbour lag 5 | 0.93 | 0 | (-0.01, 0) |
| 2-km neighbour lag 6 | 0.93 | 0 | (-0.01, 0) |
| 2-km neighbour lag 7 | 0.93 | 0 | (-0.01, 0) |
| 2-km neighbour lag 8 | 0.92 | 0 | (-0.01, 0) |
| National level cases | 1 | 0.03 | (0.02, 0.03) |
| Vegetation | 1 | -0.36 | (-0.39, -0.33) |
| Building age | 1 | 0 | (0, 0) |
| Connectivity | 1 | 0 | (0, 0) |
| Mean temperature lag 1 | 1 | 0.04 | (0.03, 0.05) |
| Mean temperature lag 2 | 0.85 | 0 | (-0.01, 0.02) |
| Mean temperature lag 3 | 0.82 | 0 | (-0.01, 0.01) |
| Mean temperature lag 4 | 0.96 | -0.01 | (-0.02, 0) |
| Mean temperature lag 5 | 1 | -0.05 | (-0.06, -0.04) |
| Max temperature lag 1 | 0.9 | 0 | (-0.01, 0) |
| Max temperature lag 2 | 1 | 0.01 | (0, 0.01) |
| Max temperature lag 3 | 0.91 | 0 | (-0.01, 0) |
| Max temperature lag 4 | 1 | 0.01 | (0.01, 0.02) |
| Max temperature lag 5 | 1 | 0.01 | (0.01, 0.02) |
| Min temperature lag 1 | 1 | -0.01 | (-0.02, 0) |
| Min temperature lag 2 | 0.88 | 0 | (-0.01, 0.01) |
| Min temperature lag 3 | 0.92 | 0 | (0, 0.01) |
| Min temperature lag 4 | 0.94 | 0 | (0, 0.01) |
| Min temperature lag 5 | 1 | 0.02 | (0.01, 0.03) |
| Mean humidity lag 1 | 1 | 0.01 | (0, 0.01) |
| Mean humidity lag 2 | 1 | 0 | (0, 0.01) |
| Mean humidity lag 3 | 1 | 0 | (0, 0) |
| Mean humidity lag 4 | 0.93 | 0 | (0, 0) |
| Mean humidity lag 5 | 1 | 0 | (0, 0) |
| Telco | 1 | 0.09 | (0.07, 0.11) |

Supplementary Table 7. Summary of parameters included in LASSO forecast model for 5-week ahead forecast. Inclusion probabilities, average estimated parameter value, 95% confidence intervals are calculated based on 1000 bootstrap samples.

| Variable | Inclusion probability (over bootstrap samples) | Estimated beta | Bootstrap confidence interval |
| --- | --- | --- | --- |
| Cases lag 1 | 1 | 0.41 | (0.35, 0.47) |
| Cases lag 2 | 1 | 0.24 | (0.19, 0.29) |
| Cases lag 3 | 1 | 0.1 | (0.06, 0.14) |
| Cases lag 4 | 1 | 0.08 | (0.04, 0.12) |
| Cases lag 5 | 1 | 0.04 | (0, 0.06) |
| Cases lag 6 | 0.92 | 0.01 | (-0.02, 0.05) |
| Cases lag 7 | 0.91 | 0.01 | (-0.02, 0.04) |
| Cases lag 8 | 0.98 | 0.03 | (0, 0.07) |
| Cases square lag 1 | 0.93 | 0.01 | (-0.02, 0.04) |
| Cases square lag 2 | 0.99 | 0.03 | (0, 0.05) |
| Cases square lag 3 | 0.94 | 0.01 | (0, 0.03) |
| Cases square lag 4 | 0.92 | 0 | (-0.01, 0.02) |
| Cases square lag 5 | 0.97 | -0.01 | (-0.03, 0) |
| Cases square lag 6 | 0.94 | -0.01 | (-0.03, 0.01) |
| Cases square lag 7 | 0.91 | -0.01 | (-0.03, 0) |
| Cases square lag 8 | 0.76 | 0 | (-0.02, 0.01) |
| Cases cubic lag 1 | 0.94 | 0 | (-0.01, 0.01) |
| Cases cubic lag 2 | 1 | -0.02 | (-0.03, -0.01) |
| Cases cubic lag 3 | 0.99 | -0.01 | (-0.02, 0) |
| Cases cubic lag 4 | 0.99 | -0.01 | (-0.02, 0) |
| Cases cubic lag 5 | 0.93 | 0 | (-0.01, 0.01) |
| Cases cubic lag 6 | 0.9 | 0 | (-0.01, 0.01) |
| Cases cubic lag 7 | 0.9 | 0 | (-0.01, 0.01) |
| Cases cubic lag 8 | 0.97 | 0 | (-0.01, 0) |
| Cases square root lag 1 | 1 | -0.3 | (-0.35, -0.25) |
| Cases square root lag 2 | 1 | -0.16 | (-0.21, -0.12) |
| Cases square root lag 3 | 0.99 | -0.05 | (-0.09, 0) |
| Cases square root lag 4 | 0.95 | -0.03 | (-0.07, 0) |
| Cases square root lag 5 | 0.9 | 0.01 | (-0.02, 0.04) |
| Cases square root lag 6 | 1 | 0.04 | (0.01, 0.07) |
| Cases square root lag 7 | 0.98 | 0.03 | (0, 0.05) |
| Cases square root lag 8 | 0.97 | 0.02 | (-0.02, 0.05) |
| 1-km neighbour lag 1 | 1 | 0.04 | (0.03, 0.04) |
| 1-km neighbour lag 2 | 1 | 0.02 | (0.01, 0.02) |
| 1-km neighbour lag 3 | 1 | 0.01 | (0, 0.01) |
| 1-km neighbour lag 4 | 0.96 | 0 | (0, 0.01) |
| 1-km neighbour lag 5 | 0.93 | 0 | (-0.01, 0.01) |
| 1-km neighbour lag 6 | 0.99 | -0.01 | (-0.01, 0) |
| 1-km neighbour lag 7 | 0.97 | 0 | (0, 0.01) |
| 1-km neighbour lag 8 | 0.97 | 0 | (0, 0.01) |
| 2-km neighbour lag 1 | 1 | 0.02 | (0.02, 0.03) |
| 2-km neighbour lag 2 | 1 | 0.01 | (0, 0.02) |
| 2-km neighbour lag 3 | 0.99 | 0.01 | (0, 0.01) |
| 2-km neighbour lag 4 | 0.93 | 0 | (-0.01, 0) |
| 2-km neighbour lag 5 | 0.94 | 0 | (-0.01, 0) |
| 2-km neighbour lag 6 | 0.93 | 0 | (-0.01, 0) |
| 2-km neighbour lag 7 | 0.94 | 0 | (-0.01, 0) |
| 2-km neighbour lag 8 | 0.93 | 0 | (-0.01, 0) |
| National level cases | 1 | 0.03 | (0.02, 0.04) |
| Vegetation | 1 | -0.39 | (-0.42, -0.35) |
| Building age | 1 | 0 | (0, 0) |
| Connectivity | 1 | 0 | (0, 0) |
| Mean temperature lag 1 | 1 | 0.04 | (0.03, 0.05) |
| Mean temperature lag 2 | 1 | 0.02 | (0.01, 0.03) |
| Mean temperature lag 3 | 0.79 | 0 | (-0.01, 0.01) |
| Mean temperature lag 4 | 0.99 | -0.02 | (-0.03, 0) |
| Mean temperature lag 5 | 1 | -0.06 | (-0.08, -0.05) |
| Max temperature lag 1 | 0.98 | 0 | (0, 0.01) |
| Max temperature lag 2 | 0.96 | 0 | (-0.01, 0) |
| Max temperature lag 3 | 1 | 0.01 | (0.01, 0.02) |
| Max temperature lag 4 | 0.96 | 0 | (0, 0.01) |
| Max temperature lag 5 | 1 | 0.02 | (0.02, 0.03) |
| Min temperature lag 1 | 1 | -0.01 | (-0.02, -0.01) |
| Min temperature lag 2 | 0.9 | 0 | (-0.01, 0) |
| Min temperature lag 3 | 0.91 | 0 | (-0.01, 0) |
| Min temperature lag 4 | 1 | 0.02 | (0.01, 0.02) |
| Min temperature lag 5 | 1 | 0.02 | (0.02, 0.03) |
| Mean humidity lag 1 | 1 | 0.01 | (0.01, 0.01) |
| Mean humidity lag 2 | 1 | 0 | (0, 0.01) |
| Mean humidity lag 3 | 1 | 0 | (0, 0) |
| Mean humidity lag 4 | 0.95 | 0 | (0, 0) |
| Mean humidity lag 5 | 1 | 0 | (-0.01, 0) |
| Telco | 1 | 0.08 | (0.06, 0.1) |

Supplementary Table 8. Summary of parameters included in LASSO forecast model for 6-week ahead forecast. Inclusion probabilities, average estimated parameter value, 95% confidence intervals are calculated based on 1000 bootstrap samples.

| Variable | Inclusion probability (over bootstrap samples) | Estimated beta | Bootstrap confidence interval |
| --- | --- | --- | --- |
| Cases lag 1 | 1 | 0.38 | (0.33, 0.44) |
| Cases lag 2 | 1 | 0.2 | (0.15, 0.25) |
| Cases lag 3 | 1 | 0.13 | (0.08, 0.18) |
| Cases lag 4 | 1 | 0.06 | (0.03, 0.1) |
| Cases lag 5 | 0.96 | 0.02 | (0, 0.06) |
| Cases lag 6 | 0.94 | 0.02 | (0, 0.05) |
| Cases lag 7 | 0.97 | 0.02 | (0, 0.06) |
| Cases lag 8 | 0.87 | 0.01 | (-0.01, 0.04) |
| Cases square lag 1 | 0.88 | 0 | (-0.02, 0.02) |
| Cases square lag 2 | 0.98 | 0.02 | (0, 0.05) |
| Cases square lag 3 | 0.97 | 0.01 | (0, 0.03) |
| Cases square lag 4 | 0.94 | -0.01 | (-0.03, 0.01) |
| Cases square lag 5 | 0.97 | -0.01 | (-0.03, 0) |
| Cases square lag 6 | 0.9 | -0.01 | (-0.03, 0.01) |
| Cases square lag 7 | 0.89 | -0.01 | (-0.03, 0.01) |
| Cases square lag 8 | 0.89 | -0.01 | (-0.03, 0.01) |
| Cases cubic lag 1 | 0.96 | 0 | (-0.01, 0.01) |
| Cases cubic lag 2 | 1 | -0.01 | (-0.02, 0) |
| Cases cubic lag 3 | 1 | -0.01 | (-0.02, 0) |
| Cases cubic lag 4 | 0.98 | -0.01 | (-0.01, 0) |
| Cases cubic lag 5 | 0.93 | 0 | (-0.01, 0.01) |
| Cases cubic lag 6 | 0.91 | 0 | (-0.01, 0.01) |
| Cases cubic lag 7 | 0.9 | 0 | (-0.01, 0.01) |
| Cases cubic lag 8 | 0.95 | 0 | (-0.01, 0) |
| Cases square root lag 1 | 1 | -0.27 | (-0.31, -0.22) |
| Cases square root lag 2 | 1 | -0.13 | (-0.17, -0.08) |
| Cases square root lag 3 | 1 | -0.07 | (-0.11, -0.02) |
| Cases square root lag 4 | 0.88 | -0.01 | (-0.05, 0.02) |
| Cases square root lag 5 | 0.99 | 0.03 | (0, 0.06) |
| Cases square root lag 6 | 0.96 | 0.02 | (-0.01, 0.05) |
| Cases square root lag 7 | 0.97 | 0.02 | (-0.01, 0.06) |
| Cases square root lag 8 | 0.99 | 0.03 | (0, 0.06) |
| 1-km neighbour lag 1 | 1 | 0.03 | (0.03, 0.04) |
| 1-km neighbour lag 2 | 1 | 0.02 | (0.01, 0.02) |
| 1-km neighbour lag 3 | 1 | 0.01 | (0, 0.01) |
| 1-km neighbour lag 4 | 0.93 | 0 | (0, 0.01) |
| 1-km neighbour lag 5 | 0.98 | 0 | (-0.01, 0) |
| 1-km neighbour lag 6 | 0.96 | 0 | (0, 0.01) |
| 1-km neighbour lag 7 | 0.95 | 0 | (0, 0.01) |
| 1-km neighbour lag 8 | 0.95 | 0 | (0, 0.01) |
| 2-km neighbour lag 1 | 1 | 0.02 | (0.02, 0.03) |
| 2-km neighbour lag 2 | 1 | 0.01 | (0.01, 0.02) |
| 2-km neighbour lag 3 | 0.99 | 0 | (0, 0.01) |
| 2-km neighbour lag 4 | 0.93 | 0 | (-0.01, 0) |
| 2-km neighbour lag 5 | 0.92 | 0 | (-0.01, 0) |
| 2-km neighbour lag 6 | 0.93 | 0 | (-0.01, 0) |
| 2-km neighbour lag 7 | 0.95 | 0 | (-0.01, 0) |
| 2-km neighbour lag 8 | 0.96 | 0 | (-0.01, 0) |
| National level cases | 1 | 0.03 | (0.02, 0.04) |
| Vegetation | 1 | -0.42 | (-0.45, -0.39) |
| Building age | 1 | 0 | (0, 0) |
| Connectivity | 1 | 0 | (0, 0) |
| Mean temperature lag 1 | 1 | 0.05 | (0.04, 0.06) |
| Mean temperature lag 2 | 0.99 | 0.02 | (0, 0.03) |
| Mean temperature lag 3 | 0.83 | 0 | (-0.01, 0.01) |
| Mean temperature lag 4 | 1 | -0.03 | (-0.04, -0.01) |
| Mean temperature lag 5 | 1 | -0.07 | (-0.08, -0.05) |
| Max temperature lag 1 | 0.94 | 0 | (-0.01, 0.03) |
| Max temperature lag 2 | 1 | 0.01 | (0, 0.01) |
| Max temperature lag 3 | 0.97 | 0 | (0, 0.01) |
| Max temperature lag 4 | 1 | 0.01 | (0, 0.02) |
| Max temperature lag 5 | 1 | 0.03 | (0.02, 0.03) |
| Min temperature lag 1 | 1 | -0.01 | (-0.02, -0.01) |
| Min temperature lag 2 | 0.98 | -0.01 | (-0.02, 0.03) |
| Min temperature lag 3 | 1 | 0.01 | (0, 0.02) |
| Min temperature lag 4 | 1 | 0.02 | (0.01, 0.03) |
| Min temperature lag 5 | 1 | 0.02 | (0.01, 0.02) |
| Mean humidity lag 1 | 1 | 0.01 | (0.01, 0.01) |
| Mean humidity lag 2 | 1 | 0 | (0, 0.01) |
| Mean humidity lag 3 | 1 | 0 | (0, 0.03) |
| Mean humidity lag 4 | 1 | 0 | (0, 0.03) |
| Mean humidity lag 5 | 1 | -0.01 | (-0.01, -0.01) |
| Telco | 1 | 0.08 | (0.06, 0.11) |

Supplementary Table 9. Summary of parameters included in LASSO forecast model for 7-week ahead forecast. Inclusion probabilities, average estimated parameter value, 95% confidence intervals are calculated based on 1000 bootstrap samples.

| Variable | Inclusion probability (over bootstrap samples) | Estimated beta | Bootstrap confidence interval |
| --- | --- | --- | --- |
| Cases lag 1 | 1 | 0.33 | (0.28, 0.38) |
| Cases lag 2 | 1 | 0.22 | (0.17, 0.31) |
| Cases lag 3 | 1 | 0.1 | (0.06, 0.15) |
| Cases lag 4 | 1 | 0.04 | (0.01, 0.08) |
| Cases lag 5 | 0.97 | 0.03 | (0, 0.06) |
| Cases lag 6 | 0.99 | 0.03 | (0, 0.07) |
| Cases lag 7 | 0.84 | 0.01 | (-0.02, 0.03) |
| Cases lag 8 | 0.8 | 0 | (-0.02, 0.04) |
| Cases square lag 1 | 0.84 | 0 | (-0.02, 0.02) |
| Cases square lag 2 | 0.95 | 0.02 | (0, 0.04) |
| Cases square lag 3 | 0.92 | 0 | (-0.02, 0.01) |
| Cases square lag 4 | 0.96 | -0.01 | (-0.03, 0.01) |
| Cases square lag 5 | 0.94 | -0.01 | (-0.03, 0.01) |
| Cases square lag 6 | 0.92 | -0.01 | (-0.03, 0.01) |
| Cases square lag 7 | 0.95 | -0.01 | (-0.04, 0) |
| Cases square lag 8 | 0.81 | 0 | (-0.02, 0.01) |
| Cases cubic lag 1 | 0.91 | 0 | (-0.01, 0.01) |
| Cases cubic lag 2 | 1 | -0.02 | (-0.03, -0.01) |
| Cases cubic lag 3 | 1 | -0.01 | (-0.02, 0) |
| Cases cubic lag 4 | 0.98 | -0.01 | (-0.01, 0) |
| Cases cubic lag 5 | 0.9 | 0 | (-0.01, 0.01) |
| Cases cubic lag 6 | 0.91 | 0 | (-0.01, 0.01) |
| Cases cubic lag 7 | 0.87 | 0 | (-0.01, 0.01) |
| Cases cubic lag 8 | 0.93 | 0 | (-0.01, 0) |
| Cases square root lag 1 | 1 | -0.23 | (-0.27, -0.18) |
| Cases square root lag 2 | 1 | -0.15 | (-0.21, -0.1) |
| Cases square root lag 3 | 0.97 | -0.04 | (-0.09, 0) |
| Cases square root lag 4 | 0.92 | 0.02 | (-0.02, 0.05) |
| Cases square root lag 5 | 0.94 | 0.02 | (-0.01, 0.05) |
| Cases square root lag 6 | 0.96 | 0.02 | (-0.02, 0.05) |
| Cases square root lag 7 | 0.99 | 0.04 | (0, 0.07) |
| Cases square root lag 8 | 0.99 | 0.03 | (0, 0.06) |
| 1-km neighbour lag 1 | 1 | 0.03 | (0.03, 0.04) |
| 1-km neighbour lag 2 | 1 | 0.02 | (0.01, 0.03) |
| 1-km neighbour lag 3 | 1 | 0.01 | (0, 0.01) |
| 1-km neighbour lag 4 | 0.95 | 0 | (-0.01, 0) |
| 1-km neighbour lag 5 | 0.98 | 0 | (0, 0.01) |
| 1-km neighbour lag 6 | 0.94 | 0 | (0, 0.01) |
| 1-km neighbour lag 7 | 0.95 | 0 | (0, 0.01) |
| 1-km neighbour lag 8 | 0.94 | 0 | (-0.01, 0) |
| 2-km neighbour lag 1 | 1 | 0.02 | (0.02, 0.03) |
| 2-km neighbour lag 2 | 1 | 0.01 | (0.01, 0.02) |
| 2-km neighbour lag 3 | 0.97 | 0 | (0, 0.01) |
| 2-km neighbour lag 4 | 0.94 | 0 | (0, 0.01) |
| 2-km neighbour lag 5 | 0.93 | 0 | (-0.01, 0) |
| 2-km neighbour lag 6 | 0.92 | 0 | (-0.01, 0) |
| 2-km neighbour lag 7 | 0.98 | 0 | (-0.01, 0) |
| 2-km neighbour lag 8 | 0.92 | 0 | (0, 0.01) |
| National level cases | 1 | 0.03 | (0.02, 0.04) |
| Vegetation | 1 | -0.44 | (-0.47, -0.4) |
| Building age | 1 | 0 | (0, 0) |
| Connectivity | 1 | 0 | (0, 0) |
| Mean temperature lag 1 | 1 | 0.06 | (0.04, 0.07) |
| Mean temperature lag 2 | 1 | 0.02 | (0, 0.03) |
| Mean temperature lag 3 | 0.95 | -0.01 | (-0.02, 0) |
| Mean temperature lag 4 | 1 | -0.03 | (-0.04, -0.01) |
| Mean temperature lag 5 | 1 | -0.07 | (-0.08, -0.06) |
| Max temperature lag 1 | 0.98 | 0.01 | (0, 0.01) |
| Max temperature lag 2 | 0.91 | 0 | (-0.01, 0.01) |
| Max temperature lag 3 | 1 | 0.01 | (0, 0.02) |
| Max temperature lag 4 | 1 | 0.02 | (0.01, 0.02) |
| Max temperature lag 5 | 1 | 0.02 | (0.02, 0.03) |
| Min temperature lag 1 | 1 | -0.02 | (-0.03, -0.01) |
| Min temperature lag 2 | 0.9 | 0 | (0, 0.01) |
| Min temperature lag 3 | 1 | 0.01 | (0.01, 0.02) |
| Min temperature lag 4 | 1 | 0.01 | (0.01, 0.02) |
| Min temperature lag 5 | 1 | 0.02 | (0.01, 0.03) |
| Mean humidity lag 1 | 1 | 0.01 | (0.01, 0.01) |
| Mean humidity lag 2 | 1 | 0 | (0, 0.01) |
| Mean humidity lag 3 | 1 | 0 | (0, 0) |
| Mean humidity lag 4 | 0.98 | 0 | (0, 0) |
| Mean humidity lag 5 | 1 | -0.01 | (-0.01, -0.01) |
| Telco | 1 | 0.08 | (0.06, 0.1) |

Supplementary Table 10. Summary of parameters included in LASSO forecast model for 8-week ahead forecast. Inclusion probabilities, average estimated parameter value, 95% confidence intervals are calculated based on 1000 bootstrap samples.

| Variable | Inclusion probability (over bootstrap samples) | Estimated beta | Bootstrap confidence interval |
| --- | --- | --- | --- |
| Cases lag 1 | 1 | 0.34 | (0.28, 0.39) |
| Cases lag 2 | 1 | 0.18 | (0.13, 0.24) |
| Cases lag 3 | 1 | 0.07 | (0.03, 0.12) |
| Cases lag 4 | 1 | 0.05 | (0.01, 0.08) |
| Cases lag 5 | 1 | 0.04 | (0, 0.08) |
| Cases lag 6 | 0.87 | 0.01 | (-0.02, 0.04) |
| Cases lag 7 | 0.79 | 0 | (-0.03, 0.03) |
| Cases lag 8 | 0.82 | 0.01 | (-0.02, 0.04) |
| Cases square lag 1 | 0.87 | 0 | (-0.02, 0.02) |
| Cases square lag 2 | 0.96 | 0 | (-0.01, 0.02) |
| Cases square lag 3 | 0.93 | 0 | (-0.02, 0.02) |
| Cases square lag 4 | 0.94 | 0 | (-0.02, 0.02) |
| Cases square lag 5 | 0.95 | 0 | (-0.02, 0.01) |
| Cases square lag 6 | 0.95 | -0.01 | (-0.03, 0.01) |
| Cases square lag 7 | 0.92 | -0.01 | (-0.04, 0) |
| Cases square lag 8 | 0.83 | 0 | (-0.02, 0.01) |
| Cases cubic lag 1 | 0.96 | 0 | (-0.01, 0) |
| Cases cubic lag 2 | 1 | -0.01 | (-0.02, 0) |
| Cases cubic lag 3 | 1 | -0.01 | (-0.02, 0) |
| Cases cubic lag 4 | 0.96 | 0 | (-0.01, 0) |
| Cases cubic lag 5 | 0.95 | 0 | (-0.01, 0) |
| Cases cubic lag 6 | 0.94 | 0 | (-0.01, 0.01) |
| Cases cubic lag 7 | 0.9 | 0 | (-0.01, 0.01) |
| Cases cubic lag 8 | 0.96 | 0 | (-0.01, 0) |
| Cases square root lag 1 | 1 | -0.23 | (-0.27, -0.18) |
| Cases square root lag 2 | 1 | -0.1 | (-0.15, -0.06) |
| Cases square root lag 3 | 0.86 | 0 | (-0.04, 0.04) |
| Cases square root lag 4 | 0.9 | 0 | (-0.04, 0.04) |
| Cases square root lag 5 | 0.94 | 0.01 | (-0.03, 0.05) |
| Cases square root lag 6 | 0.99 | 0.04 | (0, 0.07) |
| Cases square root lag 7 | 1 | 0.04 | (0.01, 0.07) |
| Cases square root lag 8 | 0.98 | 0.03 | (0, 0.06) |
| 1-km neighbour lag 1 | 1 | 0.03 | (0.03, 0.04) |
| 1-km neighbour lag 2 | 1 | 0.02 | (0.01, 0.02) |
| 1-km neighbour lag 3 | 0.97 | 0 | (0, 0.01) |
| 1-km neighbour lag 4 | 0.99 | 0.01 | (0, 0.01) |
| 1-km neighbour lag 5 | 0.96 | 0 | (0, 0.01) |
| 1-km neighbour lag 6 | 0.93 | 0 | (-0.01, 0.01) |
| 1-km neighbour lag 7 | 0.96 | 0 | (-0.01,0) |
| 1-km neighbour lag 8 | 0.99 | 0.01 | (0, 0.01) |
| 2-km neighbour lag 1 | 1 | 0.02 | (0.02, 0.03) |
| 2-km neighbour lag 2 | 1 | 0.01 | (0.01, 0.02) |
| 2-km neighbour lag 3 | 0.99 | 0.01 | (0, 0.01) |
| 2-km neighbour lag 4 | 0.92 | 0 | (0, 0.01) |
| 2-km neighbour lag 5 | 0.93 | 0 | (-0.01, 0.01) |
| 2-km neighbour lag 6 | 0.96 | 0 | (-0.01, 0) |
| 2-km neighbour lag 7 | 0.93 | 0 | (0, 0.01) |
| 2-km neighbour lag 8 | 0.99 | 0 | (-0.01, 0) |
| National level cases | 1 | 0.02 | (0.02, 0.03) |
| Vegetation | 1 | -0.46 | (-0.5, -0.42) |
| Building age | 1 | 0 | (0, 0) |
| Connectivity | 1 | 0 | (0, 0) |
| Mean temperature lag 1 | 1 | 0.06 | (0.05, 0.07) |
| Mean temperature lag 2 | 0.93 | 0.01 | (0, 0.02) |
| Mean temperature lag 3 | 0.94 | -0.01 | (-0.02, 0) |
| Mean temperature lag 4 | 1 | -0.03 | (-0.04, -0.02) |
| Mean temperature lag 5 | 1 | -0.07 | (-0.09, -0.06) |
| Max temperature lag 1 | 0.91 | 0 | (-0.01, 0.01) |
| Max temperature lag 2 | 0.99 | 0.01 | (0, 0.01) |
| Max temperature lag 3 | 1 | 0.02 | (0.01, 0.02) |
| Max temperature lag 4 | 1 | 0.01 | (0, 0.02) |
| Max temperature lag 5 | 1 | 0.02 | (0.02, 0.03) |
| Min temperature lag 1 | 1 | -0.01 | (-0.02, 0) |
| Min temperature lag 2 | 0.99 | 0.01 | (0, 0.02) |
| Min temperature lag 3 | 0.92 | 0 | (-0.01, 0.01) |
| Min temperature lag 4 | 1 | 0.02 | (0.01, 0.03) |
| Min temperature lag 5 | 1 | 0.02 | (0.01, 0.03) |
| Mean humidity lag 1 | 1 | 0.01 | (0.01, 0.01) |
| Mean humidity lag 2 | 1 | 0.01 | (0.01, 0.01) |
| Mean humidity lag 3 | 0.92 | 0 | (0, 0) |
| Mean humidity lag 4 | 0.97 | 0 | (0, 0) |
| Mean humidity lag 5 | 1 | -0.01 | (-0.01, -0.01) |
| Telco | 1 | 0.08 | (0.06, 0.1) |

Supplementary Table 11. Summary of parameters included in LASSO forecast model for 9-week ahead forecast. Inclusion probabilities, average estimated parameter value, 95% confidence intervals are calculated based on 1000 bootstrap samples.

| Variable | Inclusion probability (over bootstrap samples) | Estimated beta | Bootstrap confidence interval |
| --- | --- | --- | --- |
| Cases lag 1 | 1 | 0.3 | (0.23, 0.35) |
| Cases lag 2 | 1 | 0.15 | (0.11, 0.22) |
| Cases lag 3 | 1 | 0.08 | (0.04, 0.12) |
| Cases lag 4 | 1 | 0.06 | (0.02, 0.1) |
| Cases lag 5 | 0.96 | 0.01 | (-0.02, 0.05) |
| Cases lag 6 | 0.86 | 0 | (-0.03, 0.03) |
| Cases lag 7 | 0.81 | 0 | (-0.03, 0.03) |
| Cases lag 8 | 0.86 | 0.01 | (-0.02, 0.05) |
| Cases square lag 1 | 0.97 | -0.01 | (-0.03, 0.01) |
| Cases square lag 2 | 0.97 | 0 | (-0.02, 0.02) |
| Cases square lag 3 | 0.95 | 0 | (-0.01, 0.02) |
| Cases square lag 4 | 0.94 | 0 | (-0.02, 0.02) |
| Cases square lag 5 | 0.96 | -0.01 | (-0.04, 0) |
| Cases square lag 6 | 0.95 | -0.01 | (-0.03, 0.01) |
| Cases square lag 7 | 0.94 | -0.01 | (-0.03, 0.01) |
| Cases square lag 8 | 0.85 | 0 | (-0.01, 0.02) |
| Cases cubic lag 1 | 0.97 | 0 | (-0.01, 0) |
| Cases cubic lag 2 | 1 | -0.01 | (-0.02, 0) |
| Cases cubic lag 3 | 0.99 | -0.01 | (-0.02, 0) |
| Cases cubic lag 4 | 0.97 | -0.01 | (-0.02, 0) |
| Cases cubic lag 5 | 0.94 | 0 | (-0.01, 0.01) |
| Cases cubic lag 6 | 0.93 | 0 | (-0.01, 0.01) |
| Cases cubic lag 7 | 0.9 | 0 | (-0.01, 0.01) |
| Cases cubic lag 8 | 0.98 | 0 | (-0.01, 0) |
| Cases square root lag 1 | 1 | -0.19 | (-0.24, -0.14) |
| Cases square root lag 2 | 1 | -0.07 | (-0.12, -0.02) |
| Cases square root lag 3 | 0.94 | -0.02 | (-0.07, 0.01) |
| Cases square root lag 4 | 0.94 | 0 | (-0.05, 0.04) |
| Cases square root lag 5 | 0.98 | 0.03 | (0, 0.07) |
| Cases square root lag 6 | 1 | 0.04 | (0.01, 0.07) |
| Cases square root lag 7 | 1 | 0.04 | (0.01, 0.07) |
| Cases square root lag 8 | 0.98 | 0.03 | (0, 0.06) |
| 1-km neighbour lag 1 | 1 | 0.03 | (0.02, 0.03) |
| 1-km neighbour lag 2 | 1 | 0.01 | (0.01, 0.02) |
| 1-km neighbour lag 3 | 1 | 0.01 | (0.01, 0.02) |
| 1-km neighbour lag 4 | 0.98 | 0.01 | (0, 0.01) |
| 1-km neighbour lag 5 | 0.95 | 0 | (-0.01, 0.01) |
| 1-km neighbour lag 6 | 0.99 | -0.01 | (-0.01, 0) |
| 1-km neighbour lag 7 | 0.97 | 0 | (0, 0.01) |
| 1-km neighbour lag 8 | 1 | 0.01 | (0, 0.01) |
| 2-km neighbour lag 1 | 1 | 0.02 | (0.02, 0.03) |
| 2-km neighbour lag 2 | 1 | 0.01 | (0.01, 0.02) |
| 2-km neighbour lag 3 | 0.99 | 0.01 | (0, 0.01) |
| 2-km neighbour lag 4 | 0.95 | 0 | (0, 0.01) |
| 2-km neighbour lag 5 | 0.96 | 0 | (-0.01, 0) |
| 2-km neighbour lag 6 | 0.95 | 0 | (0, 0.01) |
| 2-km neighbour lag 7 | 1 | -0.01 | (-0.01, 0) |
| 2-km neighbour lag 8 | 0.93 | 0 | (0, 0.01) |
| National level cases | 1 | 0.02 | (0.01, 0.03) |
| Vegetation | 1 | -0.48 | (-0.51, -0.44) |
| Building age | 1 | 0 | (0, 0) |
| Connectivity | 1 | 0 | (0, 0) |
| Mean temperature lag 1 | 1 | 0.05 | (0.04, 0.07) |
| Mean temperature lag 2 | 0.94 | 0.01 | (0, 0.02) |
| Mean temperature lag 3 | 0.93 | -0.01 | (-0.02, 0.01) |
| Mean temperature lag 4 | 1 | -0.03 | (-0.05, -0.02) |
| Mean temperature lag 5 | 1 | -0.08 | (-0.09, -0.07) |
| Max temperature lag 1 | 0.99 | 0.01 | (0, 0.01) |
| Max temperature lag 2 | 1 | 0.02 | (0.01, 0.02) |
| Max temperature lag 3 | 1 | 0.01 | (0.01, 0.02) |
| Max temperature lag 4 | 1 | 0.01 | (0.01, 0.02) |
| Max temperature lag 5 | 1 | 0.02 | (0.01, 0.02) |
| Min temperature lag 1 | 0.98 | -0.01 | (-0.01, 0) |
| Min temperature lag 2 | 0.97 | -0.01 | (-0.01, 0) |
| Min temperature lag 3 | 1 | 0.01 | (0, 0.02) |
| Min temperature lag 4 | 1 | 0.01 | (0.01, 0.02) |
| Min temperature lag 5 | 1 | 0.03 | (0.02, 0.04) |
| Mean humidity lag 1 | 1 | 0.01 | (0.01, 0.01) |
| Mean humidity lag 2 | 1 | 0 | (0, 0) |
| Mean humidity lag 3 | 0.96 | 0 | (0, 0) |
| Mean humidity lag 4 | 0.98 | 0 | (0, 0) |
| Mean humidity lag 5 | 1 | -0.01 | (-0.01, -0.01) |
| Telco | 1 | 0.1 | (0.08, 0.12) |

Supplementary Table 12. Summary of parameters included in LASSO forecast model for 10-week ahead forecast. Inclusion probabilities, average estimated parameter value, 95% confidence intervals are calculated based on 1000 bootstrap samples.

| Variable | Inclusion probability (over bootstrap samples) | Estimated beta | Bootstrap confidence interval |
| --- | --- | --- | --- |
| Cases lag 1 | 1 | 0.27 | (0.22, 0.33) |
| Cases lag 2 | 1 | 0.15 | (0.1, 0.2) |
| Cases lag 3 | 1 | 0.09 | (0.05, 0.14) |
| Cases lag 4 | 0.99 | 0.03 | (0, 0.07) |
| Cases lag 5 | 0.89 | 0.01 | (-0.02, 0.04) |
| Cases lag 6 | 0.83 | 0 | (-0.04, 0.03) |
| Cases lag 7 | 0.75 | 0 | (-0.03, 0.03) |
| Cases lag 8 | 0.98 | 0.04 | (0, 0.08) |
| Cases square lag 1 | 0.98 | -0.02 | (-0.04, 0) |
| Cases square lag 2 | 0.98 | 0 | (-0.02, 0.02) |
| Cases square lag 3 | 0.96 | 0 | (-0.02, 0.02) |
| Cases square lag 4 | 0.94 | -0.01 | (-0.03, 0.01) |
| Cases square lag 5 | 0.94 | -0.01 | (-0.03, 0.01) |
| Cases square lag 6 | 0.95 | -0.01 | (-0.03, 0) |
| Cases square lag 7 | 0.92 | -0.01 | (-0.03, 0) |
| Cases square lag 8 | 0.84 | 0.01 | (-0.01, 0.03) |
| Cases cubic lag 1 | 0.98 | 0 | (-0.01, 0) |
| Cases cubic lag 2 | 0.99 | -0.01 | (-0.02, 0) |
| Cases cubic lag 3 | 0.99 | -0.01 | (-0.02, 0) |
| Cases cubic lag 4 | 0.97 | 0 | (-0.01, 0) |
| Cases cubic lag 5 | 0.93 | 0 | (-0.01, 0.01) |
| Cases cubic lag 6 | 0.92 | 0 | (-0.01, 0.01) |
| Cases cubic lag 7 | 0.9 | 0 | (0, 0.01) |
| Cases cubic lag 8 | 1 | -0.01 | (-0.02, 0) |
| Cases square root lag 1 | 1 | -0.15 | (-0.2, -0.11) |
| Cases square root lag 2 | 1 | -0.08 | (-0.12, -0.03) |
| Cases square root lag 3 | 0.96 | -0.02 | (-0.07, 0.01) |
| Cases square root lag 4 | 0.96 | 0.02 | (-0.01, 0.06) |
| Cases square root lag 5 | 0.99 | 0.03 | (0, 0.07) |
| Cases square root lag 6 | 1 | 0.04 | (0.01, 0.07) |
| Cases square root lag 7 | 1 | 0.05 | (0.01, 0.08) |
| Cases square root lag 8 | 0.93 | 0.01 | (-0.03, 0.05) |
| 1-km neighbour lag 1 | 1 | 0.02 | (0.02, 0.03) |
| 1-km neighbour lag 2 | 1 | 0.02 | (0.02, 0.03) |
| 1-km neighbour lag 3 | 1 | 0.01 | (0, 0.02) |
| 1-km neighbour lag 4 | 0.98 | 0 | (0, 0.01) |
| 1-km neighbour lag 5 | 0.97 | 0 | (-0.01, 0) |
| 1-km neighbour lag 6 | 0.94 | 0 | (0, 0.01) |
| 1-km neighbour lag 7 | 0.98 | 0 | (0, 0.01) |
| 1-km neighbour lag 8 | 1 | 0.01 | (0, 0.01) |
| 2-km neighbour lag 1 | 1 | 0.02 | (0.02, 0.03) |
| 2-km neighbour lag 2 | 1 | 0.01 | (0.01, 0.03) |
| 2-km neighbour lag 3 | 1 | 0.01 | (0, 0.01) |
| 2-km neighbour lag 4 | 0.92 | 0 | (-0.01, 0.01) |
| 2-km neighbour lag 5 | 0.96 | 0 | (0, 0.01) |
| 2-km neighbour lag 6 | 1 | -0.01 | (-0.01, 0) |
| 2-km neighbour lag 7 | 0.94 | 0 | (-0.01, 0) |
| 2-km neighbour lag 8 | 0.94 | 0 | (-0.01, 0.01) |
| National level cases | 1 | 0.01 | (0.01, 0.02) |
| Vegetation | 1 | -0.49 | (-0.53, -0.46) |
| Building age | 1 | 0 | (0, 0) |
| Connectivity | 1 | 0 | (0, 0) |
| Mean temperature lag 1 | 1 | 0.06 | (0.04, 0.07) |
| Mean temperature lag 2 | 0.98 | 0.01 | (0, 0.03) |
| Mean temperature lag 3 | 0.92 | -0.01 | (-0.02, 0) |
| Mean temperature lag 4 | 1 | -0.03 | (-0.05, -0.02) |
| Mean temperature lag 5 | 1 | -0.09 | (-0.11, -0.08) |
| Max temperature lag 1 | 1 | 0.02 | (0.01, 0.02) |
| Max temperature lag 2 | 1 | 0.01 | (0, 0.02) |
| Max temperature lag 3 | 1 | 0.01 | (0.01, 0.02) |
| Max temperature lag 4 | 1 | 0.01 | (0, 0.02) |
| Max temperature lag 5 | 1 | 0.02 | (0.02, 0.03) |
| Min temperature lag 1 | 1 | -0.02 | (-0.03, -0.01) |
| Min temperature lag 2 | 0.93 | 0 | (-0.01, 0.01) |
| Min temperature lag 3 | 0.93 | 0 | (-0.01, 0.01) |
| Min temperature lag 4 | 1 | 0.03 | (0.02, 0.04) |
| Min temperature lag 5 | 1 | 0.04 | (0.03, 0.04) |
| Mean humidity lag 1 | 1 | 0.01 | (0.01, 0.01) |
| Mean humidity lag 2 | 1 | 0 | (0, 0.01) |
| Mean humidity lag 3 | 0.97 | 0 | (0, 0) |
| Mean humidity lag 4 | 0.96 | 0 | (0, 0) |
| Mean humidity lag 5 | 1 | -0.01 | (-0.01, -0.01) |
| Telco | 1 | 0.1 | (0.07, 0.12) |

Supplementary Table 13. Summary of parameters included in LASSO forecast model for 11-week ahead forecast. Inclusion probabilities, average estimated parameter value, 95% confidence intervals are calculated based on 1000 bootstrap samples.

| Variable | Inclusion probability (over bootstrap samples) | Estimated beta | Bootstrap confidence interval |
| --- | --- | --- | --- |
| Cases lag 1 | 1 | 0.26 | (0.2, 0.32) |
| Cases lag 2 | 1 | 0.15 | (0.09, 0.21) |
| Cases lag 3 | 1 | 0.05 | (0.01, 0.09) |
| Cases lag 4 | 0.95 | 0.02 | (-0.01, 0.05) |
| Cases lag 5 | 0.84 | 0 | (-0.03, 0.03) |
| Cases lag 6 | 0.79 | 0 | (-0.03, 0.03) |
| Cases lag 7 | 0.93 | 0.02 | (0, 0.06) |
| Cases lag 8 | 0.87 | 0.02 | (-0.01, 0.05) |
| Cases square lag 1 | 0.98 | -0.02 | (-0.04, 0) |
| Cases square lag 2 | 0.98 | 0 | (-0.02, 0.02) |
| Cases square lag 3 | 0.96 | -0.01 | (-0.02, 0.01) |
| Cases square lag 4 | 0.89 | 0 | (-0.02, 0.01) |
| Cases square lag 5 | 0.95 | -0.01 | (-0.03, 0.01) |
| Cases square lag 6 | 0.94 | -0.01 | (-0.03, 0) |
| Cases square lag 7 | 0.74 | 0 | (-0.02, 0.02) |
| Cases square lag 8 | 0.82 | 0 | (-0.01, 0.02) |
| Cases cubic lag 1 | 0.98 | 0 | (-0.01, 0) |
| Cases cubic lag 2 | 1 | -0.01 | (-0.02, 0) |
| Cases cubic lag 3 | 0.98 | -0.01 | (-0.01, 0) |
| Cases cubic lag 4 | 0.95 | 0 | (-0.01, 0.01) |
| Cases cubic lag 5 | 0.95 | 0 | (-0.01, 0.01) |
| Cases cubic lag 6 | 0.91 | 0 | (-0.01, 0.01) |
| Cases cubic lag 7 | 0.97 | -0.01 | (-0.01, 0) |
| Cases cubic lag 8 | 0.99 | -0.01 | (-0.01, 0) |
| Cases square root lag 1 | 1 | -0.16 | (-0.21, -0.11) |
| Cases square root lag 2 | 1 | -0.07 | (-0.12, -0.02) |
| Cases square root lag 3 | 0.94 | 0.01 | (-0.03, 0.04) |
| Cases square root lag 4 | 0.98 | 0.02 | (-0.01, 0.06) |
| Cases square root lag 5 | 1 | 0.04 | (0, 0.07) |
| Cases square root lag 6 | 1 | 0.05 | (0.02, 0.08) |
| Cases square root lag 7 | 0.97 | 0.02 | (-0.01, 0.06) |
| Cases square root lag 8 | 0.98 | 0.03 | (0, 0.06) |
| 1-km neighbour lag 1 | 1 | 0.03 | (0.02, 0.04) |
| 1-km neighbour lag 2 | 1 | 0.02 | (0.01, 0.02) |
| 1-km neighbour lag 3 | 1 | 0.01 | (0, 0.01) |
| 1-km neighbour lag 4 | 0.96 | 0 | (-0.01, 0) |
| 1-km neighbour lag 5 | 0.97 | 0 | (0, 0.01) |
| 1-km neighbour lag 6 | 0.97 | 0 | (0, 0.01) |
| 1-km neighbour lag 7 | 0.98 | 0 | (0, 0.01) |
| 1-km neighbour lag 8 | 0.96 | 0 | (0, 0.01) |
| 2-km neighbour lag 1 | 1 | 0.02 | (0.02, 0.03) |
| 2-km neighbour lag 2 | 1 | 0.01 | (0.01, 0.02) |
| 2-km neighbour lag 3 | 0.98 | 0 | (0, 0.01) |
| 2-km neighbour lag 4 | 0.99 | 0.01 | (0, 0.01) |
| 2-km neighbour lag 5 | 0.98 | 0 | (-0.01, 0) |
| 2-km neighbour lag 6 | 0.95 | 0 | (-0.01, 0) |
| 2-km neighbour lag 7 | 0.95 | 0 | (-0.01, 0) |
| 2-km neighbour lag 8 | 0.94 | 0 | (-0.01, 0) |
| National level cases | 1 | 0.01 | (0.01, 0.02) |
| Vegetation | 1 | -0.5 | (-0.53, -0.47) |
| Building age | 1 | 0 | (0, 0) |
| Connectivity | 1 | 0 | (0, 0) |
| Mean temperature lag 1 | 1 | 0.06 | (0.05, 0.07) |
| Mean temperature lag 2 | 0.98 | 0.02 | (0, 0.03) |
| Mean temperature lag 3 | 0.92 | -0.01 | (-0.02, 0.01) |
| Mean temperature lag 4 | 1 | -0.04 | (-0.06, -0.03) |
| Mean temperature lag 5 | 1 | -0.1 | (-0.11, -0.09) |
| Max temperature lag 1 | 1 | 0.01 | (0.01, 0.02) |
| Max temperature lag 2 | 1 | 0.02 | (0.01, 0.02) |
| Max temperature lag 3 | 1 | 0.01 | (0.01, 0.02) |
| Max temperature lag 4 | 1 | 0.01 | (0, 0.02) |
| Max temperature lag 5 | 1 | 0.02 | (0.01, 0.03) |
| Min temperature lag 1 | 1 | -0.02 | (-0.03, -0.01) |
| Min temperature lag 2 | 0.98 | -0.01 | (-0.02, 0) |
| Min temperature lag 3 | 1 | 0.01 | (0, 0.02) |
| Min temperature lag 4 | 1 | 0.03 | (0.02, 0.04) |
| Min temperature lag 5 | 1 | 0.04 | (0.03, 0.05) |
| Mean humidity lag 1 | 1 | 0.01 | (0.01, 0.01) |
| Mean humidity lag 2 | 1 | 0 | (0, 0.01) |
| Mean humidity lag 3 | 0.98 | 0 | (0, 0) |
| Mean humidity lag 4 | 0.96 | 0 | (0, 0) |
| Mean humidity lag 5 | 1 | -0.01 | (-0.01, -0.01) |
| Telco | 1 | 0.08 | (0.06, 0.11) |

Supplementary Table 14. Summary of parameters included in LASSO forecast model for 12-week ahead forecast. Inclusion probabilities, average estimated parameter value, 95% confidence intervals are calculated based on 1000 bootstrap samples.
